# PICASSO allows ultra-multiplexed fluorescence imaging of spatially overlapping proteins without reference spectra measurements

**DOI:** 10.1038/s41467-022-30168-z

**Published:** 2022-05-05

**Authors:** Junyoung Seo, Yeonbo Sim, Jeewon Kim, Hyunwoo Kim, In Cho, Hoyeon Nam, Young-Gyu Yoon, Jae-Byum Chang

**Affiliations:** 1 Department of Materials Science and Engineering, Korea Advanced Institute of Science and Technology, Daejeon, Korea; 2 Department of Biomedical Engineering, Sungkyunkwan University, Suwon, Korea; 3 School of Electrical Engineering, Korea Advanced Institute of Science and Technology, Daejeon, Korea

**Keywords:** Fluorescence imaging, Confocal microscopy, Immunohistochemistry

## Abstract

Ultra-multiplexed fluorescence imaging requires the use of spectrally overlapping fluorophores to label proteins and then to unmix the images of the fluorophores. However, doing this remains a challenge, especially in highly heterogeneous specimens, such as the brain, owing to the high degree of variation in the emission spectra of fluorophores in such specimens. Here, we propose PICASSO, which enables more than 15-color imaging of spatially overlapping proteins in a single imaging round without using any reference emission spectra. PICASSO requires an equal number of images and fluorophores, which enables such advanced multiplexed imaging, even with bandpass filter-based microscopy. We show that PICASSO can be used to achieve strong multiplexing capability in diverse applications. By combining PICASSO with cyclic immunofluorescence staining, we achieve 45-color imaging of the mouse brain in three cycles. PICASSO provides a tool for multiplexed imaging with high accessibility and accuracy for a broad range of researchers.

## Abstract (web summary)

Ultra-multiplexed fluorescence imaging is currently difficult. Here the authors report PICASSO which enables 15-colour imaging of spatially overlapping proteins in a single-round of imaging; they combine it with cyclic immunofluorescence to achieve 45-colour imaging of the mouse brain in 3 cycles.

## Introduction

Multiplexed fluorescence imaging has been extensively used in a wide range of biological and medical applications. However, the number of fluorophores that can be simultaneously used is limited to four due to spectral overlap^1^. To overcome this limitation, various forms of spectral imaging and linear unmixing (in short linear unmixing) have been developed^1^. Mathematically, linear unmixing is formulated as an inverse problem: IMG = *M* × *F*, where IMG and *F* are the acquired images (mixed images) and unmixed images, respectively, and *M* is the mixing matrix^2^. Linear unmixing can precisely unmix mixed images and has been successfully used in several studies^3–5^. The accuracy of linear unmixing depends on how precisely the mixing matrix M can be measured^6^. The mixing matrix can be measured from either single-fluorophore areas of the target specimen or from additional specimens that have been prepared identically but with only one fluorophore each^1,7^. However, we found that such reference spectra measurement could be complicated to perform in highly heterogeneous specimens, such as the brain, due to the high level of variation of the emission spectra of fluorophores depending on the subregions from which the spectra were measured (see Supplementary Fig. 1 for the effects of spectral variation on unmixing performance and Supplementary Fig. 2 for emission spectra measured from different subregions of the brain). Such variation requires that the reference spectra need to be measured from all target subregions of the brain and then used specifically for the unmixing of those subregions.

To address this problem, a different approach has been developed that does not require reference spectra measurement. This approach, termed blind unmixing, compensates for the lack of prior knowledge of the emission spectra through unsupervised learning, either by finding a low-rank representation of mixed images (e.g., via non-negative matrix factorization (NMF))^8–10^ or by clustering^11^. The former approach accurately unmixes images when a sufficiently large number of input images are provided through fluorescence lifetime imaging^10^. However, only partial success has been demonstrated in unmixing spatially overlapping proteins via conventional microscopy using a spectral detector (see Supplementary Fig. 3 for our NMF results)^8,9^. The latter approach uses unsupervised machine learning to classify pixels to the nearest cluster^11^. However, in this approach, pixels expressing more than one protein are classified into another cluster, and the ratio of the expression levels of the proteins is not measured^11^. In addition to these two approaches, an alternative approach has also been demonstrated that uses fluorophores with low cross-channel bleed-through and then unmixing their signals via orthogonalization^12^. However, the use of fluorophores with low cross-channel bleed-through limits the number of fluorophores that can be simultaneously used with one excitation laser; it would be challenging to achieve higher-level multiplexing with this approach.

Therefore, we propose a non-reference-based unmixing technique called PICASSO (Process of ultra-multiplexed Imaging of biomoleCules viA the unmixing of the Signals of Spectrally Overlapping fluorophores), which can blindly unmix images without reference emission spectra, enabling multiplexed imaging of 15 proteins in the brain in a single staining and imaging round. We devised a strategy based on information theory; unmixing is performed by iteratively minimizing the mutual information between mixed images. This allows us to get away with the assumption that the spatial distribution of different proteins is mutually exclusive, therefore enabling accurate information unmixing. By combining PICASSO with an antibody complex formation technique, we demonstrate 15-color multiplexed imaging of a mouse brain in a single staining and imaging round. We also show that PICASSO can be used for multiplexed 3D imaging, large-area imaging, mRNA imaging, super-resolution imaging through tissue expansion, tissue clearing, and the multiplexed imaging of clinical specimens. Since PICASSO can improve the multiplexing capability of cyclic immunofluorescence techniques by letting them use more fluorophores in one cycle, we can achieve 45-color multiplexed imaging of the mouse brain in only three staining and imaging cycles through Cyclic-PICASSO. Lastly, we show that PICASSO can be implemented with bandpass filter-based microscopy because it only requires the number of image acquisitions equal to the number of fluorophores.

## Results

### General working principle of PICASSO

**Fig. 1:**
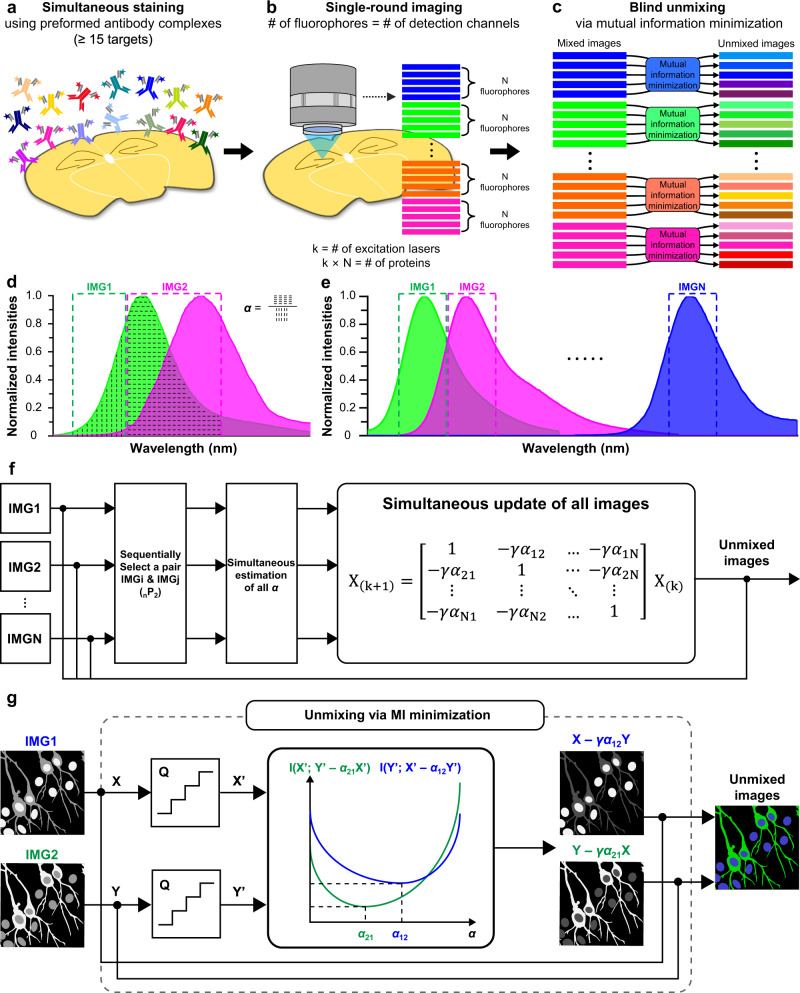
Schematic of the PICASSO process. **a**–**c** Experimental process of multiplexed immunofluorescence imaging using PICASSO. **a** Simultaneous immunostaining of more than 15 proteins with primary antibody–Fab complexes conjugated with spectrally overlapping fluorophores. **b** Acquisition of images at different detection channels. The number of required images equals the number of fluorophores to be unmixed. For each of the *k* excitation lasers, *N* spectrally overlapping fluorophores are used, making the total number of fluorophores *k* × *N*. **c** Blind source separation of *N* mixed images into *N* images, each of which contains the signal from only one fluorophore through progressive mutual information (MI) minimization. **d** Emission spectra of two spectrally overlapping fluorophores (green and magenta solid lines and colored regions) and detection channels (green and magenta dotted boxes). Here *α* is the ratio of the area with horizontal dotted lines to the area with vertical dotted lines. IMG1, 2 are images acquired at the first (green dotted box) and second (magenta dotted box) detection channels. **e** Emission spectra of *N* spectrally overlapping fluorophores and detection channels. **f**, **g** Schematic of the PICASSO unmixing algorithm. The images are unmixed by progressively minimizing the MI between mixed images. **f** *N*-color unmixing via MI minimization. **g** Two-color unmixing via MI minimization. *Q* = quantization, *X*’ and *Y*’ = quantized IMG1, 2. *I*(*X*’; *Y*’ − *α*_21_X’) = MI between *X*’ and *Y*’ − *α*_21_*X*’. *I*(*Y*’; *X*’ − *α*_12_*Y*’) = MI between *Y*’ and *X*’ − *α*_21_*Y*’. *α*_12_ and *α*_21_: optimized *α* that enables the minimization of MI.

In the experimental implementation of PICASSO, spectrally overlapping fluorophores that can be strongly excited by the same source were selected from various commercial fluorophores. If *N* fluorophores were chosen for each of the *k* excitation lasers, the total number of proteins that can be simultaneously imaged was *k* × *N*. Higher multiplexing can be achieved by adding large Stokes shift fluorophores to these fluorophores. To stain such a multitude of proteins with antibodies unlimited by the availability of host species, we used the primary antibody–Fab complex preformation technique, which enables the use of multiple primary antibodies from the same host species^13^. Each primary antibody was assembled with a Fab fragment of a secondary antibody bearing a fluorophore; all the assembled antibody complexes were then applied to specimens together (Fig. 1a). Images of specimens were then acquired at different spectral ranges, each containing the emission peak of each fluorophore (Fig. 1b). Finally, the images were unmixed via PICASSO and separated into single protein images (Fig. 1c).

The PICASSO unmixing algorithm takes *N* channel mixed images of *N* fluorophores as the input and obtains the unmixed images by iteratively subtracting scaled images from one another to minimize the mutual information (MI) between them. The main assumption underlying PICASSO is that spectral mixing results in an increase of the MI between multiple channels, meaning that the unmixed images can therefore be recovered through MI minimization. We first confirmed this assumption in a simple setting by using two spectrally overlapping fluorophores and two specific detection channels. We set the two detection channels such that the first contained the signal of only the first fluorophore while the second contained the signals of both fluorophores (Fig. 1d). We can therefore express the relationship between the images as follows:

1 $$\left[\begin{array}{c}{{{{{{\rm{IMG}}}}}}}_{1}\\ {{{{{{\rm{IMG}}}}}}}_{2}\end{array}\right]=\left[\begin{array}{cc}1 & 0\\ \alpha & 1\end{array}\right]\left[\begin{array}{c}{F}_{1}\\ {F}_{2}\end{array}\right],$$

Where IMG_1_ and IMG_2_ are the first and second images acquired in the first and the second detection channels, respectively; *F*_1_ and *F*_2_ are the images of the first and second fluorophores, respectively; and *α* is the ratio of the fluorescence intensity of the first fluorophore in the second detection channel to the fluorescence intensity of the first fluorophore in the first detection channel (Fig. 1d). The ground-truth image of the second fluorophore (*F*_2_) can then be obtained by simply subtracting the first image (IMG_1_) from the second image (IMG_2_) after scaling the first image by *α* (i.e., *F*_2_ = IMG_2_ − *α*IMG_1_) (see Supplementary Fig. 4 for details). An estimate of *α*, denoted as $\widehat{\alpha }$, can be obtained by solving a simple optimization problem as follows:

2 $$\widehat{\alpha }={{{{{\rm{arg }}}}}}\mathop{{{{{{\rm{min }}}}}}}\limits_{\alpha }{{{{{\rm{I}}}}}}\left({{{{{{\rm{IMG}}}}}}}_{1}{{;}}{{{{{{\rm{IMG}}}}}}}_{2}-\alpha {{{{{{\rm{IMG}}}}}}}_{1}\right)\;{{{{{{\rm{subject}}}}}}}\;{{{{{{\rm{to}}}}}}}\;\alpha \ge 0.$$

Through simulation, we confirmed that this simple approach was able to unmix two mixed images. Even when two proteins spatially overlapped, this approach unmixed two mixed images with an accuracy of more than 99% (Supplementary Fig. 5). We then showed that this approach was applicable to four standard lasers by performing two- or three-color multiplexed imaging on single lasers with wavelengths of 405, 488, 557, and 640 nm (Supplementary Fig. 6).

PICASSO is a generalized version of this unmixing algorithm that can handle an arbitrary number of channels and has relaxed constraints on the acquired images (Fig. 1e–g). For *N*-channel unmixing, we can express the relationships among the images as follows:

3 $$\left[\begin{array}{c}{{{{{{\rm{IMG}}}}}}}_{1}\\ {{{{{{\rm{IMG}}}}}}}_{2}\\ \vdots \\ {{{{{{\rm{IMG}}}}}}}_{N}\end{array}\right]=\left[\begin{array}{cccc}1 & {\alpha }_{1,2} & \ldots & {\alpha }_{1,N}\\ {\alpha }_{2,1} & 1 & \ddots & \vdots \\ \vdots & \ddots & \ddots & {\alpha }_{N,N-1} \\ {\alpha }_{N,1} & \ldots & {\alpha }_{N,N-1} & 1\end{array}\right]\left[\begin{array}{c}{F}_{1}\\ {F}_{2}\\ \vdots \\ {F}_{N}\end{array}\right],$$

where *α*_*i*, *j*_ refers to the relative leakage from the *j*_th_ fluorophore to the *i*_th_ image. To undo the mixing, PICASSO starts by initializing the *i*_th_ channel solution *X*_*i*(0)_ as IMG_*i*_ and progressively updates the solution based on the following equation:

4 $$\left[\begin{array}{c}{X}_{1(k+1)}\\ {X}_{2(k+1)}\\ \vdots \\ {X}_{N(k+1)}\end{array}\right]=\left[\begin{array}{cccc}1 & -\gamma {\widehat{{\alpha }_{1,2(k)}}} & \ldots & -\gamma {\widehat{{\alpha }_{1,N(k)}}}\\ -\gamma {\widehat{{\alpha }_{2,1(k)}}} & 1 & \ddots & \vdots \\ \vdots & \ddots & \ddots & -\gamma {\widehat{{\alpha }_{N-1,N(k)}}} \\ -\gamma {\widehat{{\alpha }_{N,1(k)}}} & \ldots & -\gamma {\widehat{{\alpha }_{N,N-1(k)}}} & 1\end{array}\right]\left[\begin{array}{c}{X}_{1(k)}\\ {X}_{2(k)}\\ \vdots \\ {X}_{N(k)}\end{array}\right],$$

where the *k*, *γ*, and *X*_*i*(*k*)_ denote the iteration number, update step size, and *i*_th_ channel image after *k* iterations, respectively, and $\widehat{{\alpha }_{i,j(k)}}$ is calculated as $\widehat{{\alpha }_{i,j(k)}}={{{{{\rm{arg }}}}}}\mathop{{{{{{\rm{min }}}}}}}\limits_{\alpha }I({X}_{j(k)};{X}_{i(k)}-\alpha {X}_{j(k)}).$ Conceptually, these iterative updates can be explained as follows. PICASSO starts by selecting a pair from multi-channel images; it then estimates the level of mixing between the two channels by measuring the MI. Based on the level of mixing, we subtract the scaled images from other images and repeat this process for all possible pairs. This procedure is iteratively performed to progressively minimize the MI. The algorithm for cases when *N* = 2 is illustrated in Fig. 1g. A sufficient condition for PICASSO is that first, each fluorophore appears brightest in one channel compared with other channels (not compared to other fluorophores), and second, the determinants of the sub 2 × 2 matrices of the mixing matrix that include diagonal entities are positive (see Supplementary Note 2 for a detailed description).

### Unmixing performance of PICASSO as compared to linear unmixing

**Fig. 2:**
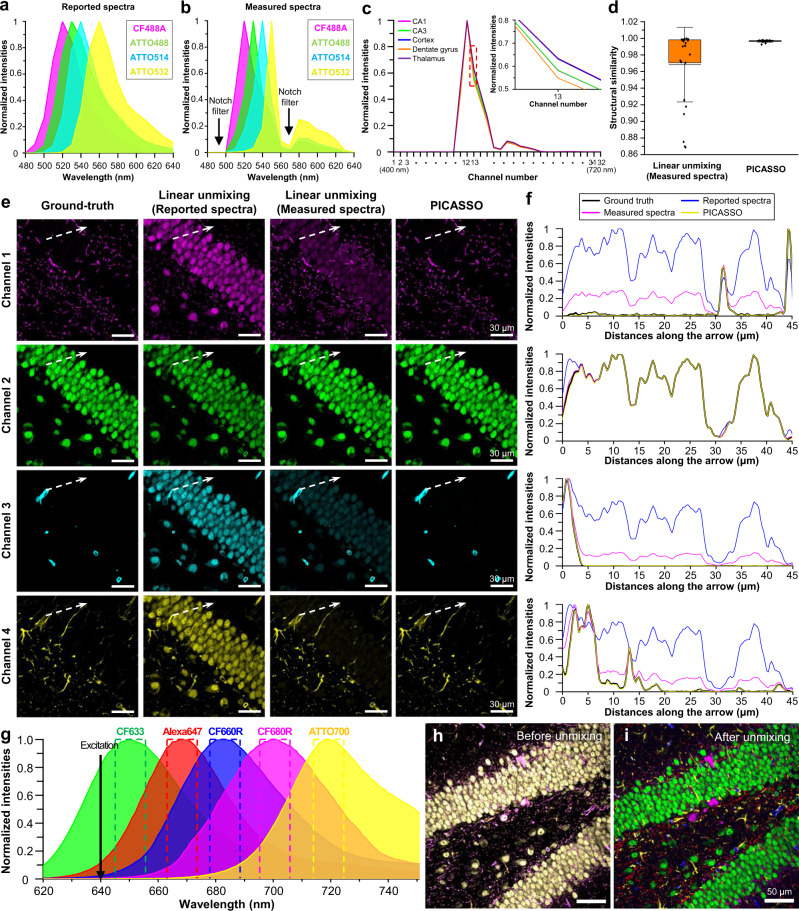
Simulation results of linear unmixing vs. PICASSO and five-color unmixing via PICASSO. **a**, **b** Emission spectra of CF488A (magenta), ATTO488 (green), ATTO514 (cyan), and ATTO532 (yellow). **a** Reported spectra. **b** Measured spectra. **c** Spectra variation of CF488A for each mouse brain subregion; the upper right inset shows a magnified view of the red dotted box. **d** Structural similarity (SSIM) between ground-truth and unmixed images. SSIM indexes were calculated for each channel and their averages were plotted on the graph. Linear unmixing: average = 0.968, lowest value = 0.869, and S.D. = 0.045. PICASSO: average = 0.997, lowest value = 0.993, and S.D. = 0.001. The center lines show the mean values and the lower and upper boundaries of the boxes correspond to 25 and 75%; the whiskers extend to show standard deviation. To compare the SSIM, 25 mixed images were synthesized by combining 25 spectra of each fluorophore (CF488A, ATTO488, ATTO514, and ATTO532) measured from *n* = 5 biologically independent subregions. **e** Ground-truth and unmixed images after linear unmixing and PICASSO. The reported spectra shown in **a**, as well as 25 measured spectra, were used to generate the 32-channel synthetic images. Averages of the 25 measurements for each fluorophore shown in **b** were used as an unmixing matrix. **f** Normalized intensities of each channel along the arrow shown in **e**. Ground-truth (black), linear unmixing (blue and magenta), and PICASSO (yellow). **g** Emission spectra of five fluorophores and detection channels used in the simulation. Solid line: emission spectrum of each fluorophore. Dotted box: detection channel defined from −5 to +5 nm of the emission peak of each fluorophore. **h**, **i** Result of five-color unmixing simulation. **h** Overlay of the five synthetic mixed images. **i** Overlay of five images after unmixing via PICASSO. Source data are provided as a Source data file.

We first studied how the unmixing accuracy of PICASSO differs from that of linear unmixing in a highly heterogeneous specimen, such as the mouse brain, through simulation. For the simulation, we chose four spectrally overlapping fluorophores (CF488A, ATTO488, ATTO514, and ATTO532) that can be simultaneously excited using a 488-nm laser (Fig. 2a). Four mouse brain slices were stained with anti-neuronal nuclei (NeuN) antibodies conjugated with one of the four fluorophores, and the emission spectrum of each fluorophore was then measured using a 32-channel spectral detector from five brain subregions, including the CA1, CA3, the cortex, the dentate gyrus, and the thalamus. The average of the measured spectra showed a different spectral shape from the reported spectra due in part to the characteristics of the instrument, including notch filters in the detection system (Fig. 2b). Furthermore, the measured spectra exhibited various characteristics depending on the brain subregions from where the spectra were acquired (Fig. 2c).

Next, we artificially synthesized 25 mixed images using the measured reference spectra and a four-color source image of a mouse brain slice in which each channel corresponded to 2′, 3′-cyclic-nucleotide 3′-phosphodiesterase (CNP1), NeuN, glucose transporter 1 (GluT1), and glial fibrillary acidic protein (GFAP). These 25 mixed images simulated the mixed images acquired in different subregions of the brain. The 25 artificially mixed images were then unmixed using both linear unmixing and PICASSO; their unmixing accuracies were then compared. For linear unmixing, the 25 synthetic mixed images, each of which contained 32 channels, were unmixed using the average of the emission spectra, as measured from various brain subregions, as the reference spectra. For PICASSO, four channels containing the emission peak of each fluorophore were chosen from the 32-channel synthetic images and unmixed, as only an equal number of channels to the number of fluorophores was required. We estimated the structural similarity (SSIM) between the unmixed images and ground-truth images to quantitatively compare the unmixing accuracy. As shown in Fig. 2d, SSIM of linear unmixing showed a large variation. When the emission spectra used to generate synthetic mixed images were similar to the average spectra, SSIM was close to 1.00. However, when the emission spectra used to generate synthetic mixed images were different from the average spectra, SSIM dropped to 0.869. This result indicates that, to achieve accurate unmixing, reference spectra should be used that are measured from the same subregion where mixed images are acquired. PICASSO, on the other hand, showed over 99% SSIM in all 25 mixed images (Fig. 2d), indicating that PICASSO can accurately unmix images acquired from diverse subregions of the brain. The variation of the emission spectra of fluorophores depending on the subregions of the brain could be attributed to the different material compositions of the subregions^14^.

The poor performance of linear unmixing was also confirmed in images. Figure 2e shows ground-truth images and both the linear unmixing and PICASSO unmixing results. We performed the linear unmixing twice with different reference spectra, the reported emission spectra shown in Fig. 2a and the average of the measured emission spectra for each fluorophore. We measured transverse intensity profiles along the white arrows in Fig. 2e and compared the profiles between ground-truth, linear unmixing with reported spectra, linear unmixing with measured spectra, and PICASSO. As shown in Fig. 2f, PICASSO (yellow line) exhibited a high level of agreement with ground-truth (black line) while linear unmixing (blue and magenta lines) showed a lower level of agreement due to the variations in the emission spectra.

We then verified whether PICASSO can unmix the fluorescence signals of five spectrally mixed fluorophores through simulation. Five spectrally overlapping fluorophores (CF633, Alexa Fluor 647, CF660R, CF680R, and ATTO700) that can be simultaneously excited by a 640-nm laser were selected. A mixing matrix was then generated by calculating the contribution of the fluorophores in five detection channels, each of which was defined from −5 to +5 nm of the emission maximum of each fluorophore (Fig. 2g). The synthetic mixed images were generated by mixing a five-color source image of a mouse brain slice with one channel each to correspond to NeuN, CNP1, GluT1, parvalbumin (PV), and GFAP with Gaussian additive noise (Fig. 2h). The noise level was determined by imaging a droplet of fluorophore solution with the same microscopy setup and imaging conditions. The synthetic images were fed to PICASSO to obtain the unmixed images (Fig. 2i). The unmixed images showed a high level of agreement with the source ground-truth images with an average SSIM of 0.951, despite the high level of spectral overlap, verifying the blind unmixing capability of the PICASSO algorithm (see Supplementary Fig. 7 for single-channel images).

We also found that PICASSO can unmix various fluorophore combinations without changing the detection bandwidth of each channel. We used the five detection channels shown in Fig. 2g and unmixed 32 different fluorophore combinations of 10 spectrally overlapping fluorophores. For all 32 tested combinations, PICASSO reliably unmixed the mixed images, showing a SSIM of unmixing of above 0.98 (Supplementary Table 1). This result indicates that PICASSO can unmix multiple different fluorophore combinations through fixed detection channels without the optimization of detection channels tailored to each fluorophore combination. This result also suggests that the same detection channel could be used to unmix given fluorophores under diverse conditions; in other words, if the emission spectra of the fluorophores shift a few nanometers due to the environmental or chemical factors described in Supplementary Note 1. Additionally, the determinants of their 2 × 2 matrices were positive for all 32 combinations (Supplementary Table 1). We further validated the performance of PICASSO by unmixing five highly overlapping fluorophores (CF488A, ATTO488, ATTO514, Alexa Fluor 514, and CF532) with only an 8–10-nm resulting difference in their emission maxima; PICASSO successfully unmixed these highly overlapping fluorophores with an SSIM of 0.99 (Supplementary Fig. 8).

### Experimental validation of PICASSO

**Fig. 3:**
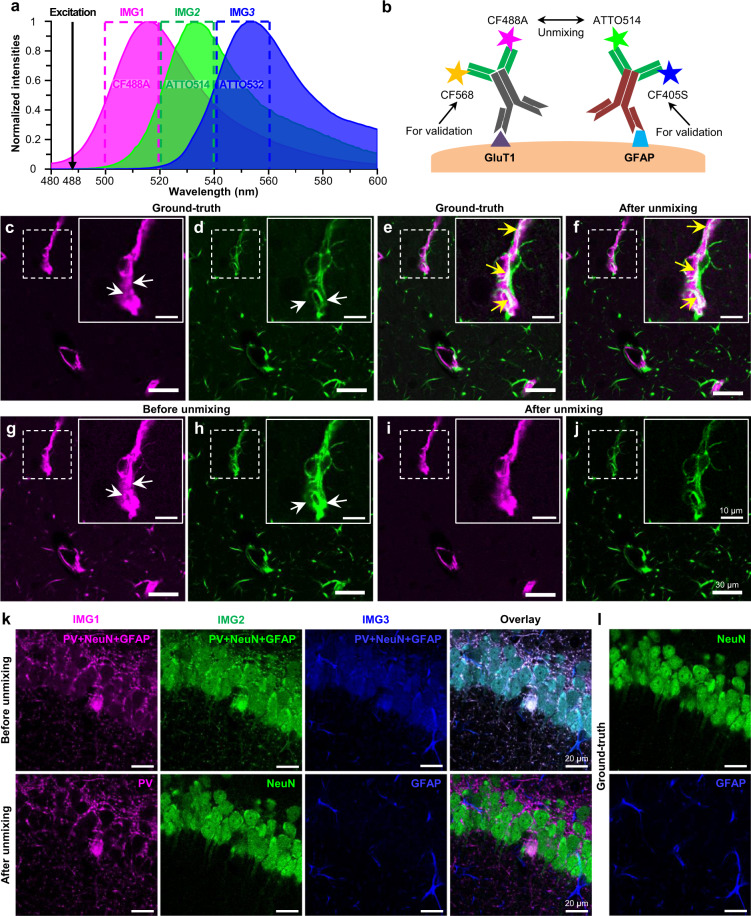
Experimental validation of unmixing of spatially overlapping proteins using PICASSO. **a** Emission spectra of the three fluorophores and three detection channels used in this experiment. The solid line and shaded area: emission spectrum of each fluorophore. Dotted box: detection channel from where images were acquired. In the two-channel validation experiment shown in **c**–**j**, only CF488A and ATTO514 were used and images were acquired from the first two detection channels. In the three-channel validation experiment shown in **k**, **l**, all three fluorophores and detection channels were used. **b** Schematic of two-channel validation experiment. **c**–**j** Ground-truth, before unmixing, and after unmixing images of the two-channel validation experiment. **c** First channel of the ground-truth image, showing GluT1. **d** Second channel of the ground-truth image, showing GFAP. **e** Overlay of the two channels of the ground-truth image. **f** Overlay of the two channels of the after-unmixing image. **g** First channel of the mixed image, acquired from the first detection range in **a**. **h** Second channel of the mixed image, acquired from the second detection range shown in **a**. **i** First channel of the unmixed image, showing GluT1. **j** Second channel of the unmixed image, showing GFAP. **k**, **l** Ground-truth, before unmixing, and after unmixing images of the three-channel validation experiment. **k** Before and after unmixing images. **l** Ground-truth images stained with separate antibodies against NeuN and GFAP.

We then experimentally validated the unmixing performance of PICASSO. Before starting the experimental validation process, we first validated the antibody complex formation technique required for the subsequent validation. We validated various aspects of this technique: the staining pattern (Supplementary Fig. 9), the crosstalks between antibodies (Supplementary Fig. 10), the compatibility of antibodies (Supplementary Fig. 11), the compatibility of fluorophores (Supplementary Table 2), and the staining depth (Supplementary Fig. 12) of the antibody preformation technique. After validating the antibody complex formation technique, we attempted to validate the unmixing performance of PICASSO in imaging spatially overlapping proteins labeled with spectrally overlapping fluorophores (Fig. 3a). We first stained a mouse brain slice with two antibody complexes, one against GluT1 conjugated with CF488A and CF568 and the other against GFAP conjugated with ATTO514 and CF405S (Fig. 3b). Two mixed images (*IMG*1 and *IMG*2 in Fig. 3a) composed of signals of both GluT1 and GFAP were obtained from two detection channels using a 488-nm excitation laser; they were then unmixed via PICASSO. For validation, ground-truth images of GluT1 and GFAP were acquired using a 561-nm and 405-nm excitation laser (Fig. 3c, d), respectively. The overlay of the two channels of the ground-truth image shown in Fig. 3e clearly showed the spatial overlap of the two proteins (yellow arrows). The unmixed images shown in Fig. 3f matched with the ground-truth image, indicating that PICASSO successfully unmixed two mixed images of spatially overlapping proteins. We measured SSIM between the ground-truth and unmixed images, and it was 0.98 (Supplementary Fig. 13). Figure 3g–j show single-channel images of both mixed and unmixed images. When the first channels of the ground-truth (Fig. 3c) and mixed (Fig. 3g) images were compared, higher fluorescence signals were observed in multiple regions in the mixed images due to the bleed-through signals of ATTO514 (white arrows in Fig. 3g). Also, in the second channel images, such bleed-through of CF488A was observed in the mixed images (white arrows in Fig. 3h). Such bleed-throughs were removed after unmixing, as shown in Fig. 3i, j. Additionally, we carried out unmixing of two spectrally overlapping fluorophores (CF633 and CF660R) by adjusting the fluorophore concentration to quantitatively analyze the performance of PICASSO (Supplementary Fig. 14). The measured signal intensities of the unmixed images were well recovered to those before unmixing, showing linearity based on the fluorophore concentration. In other words, PICASSO might not be affected by the ratio of the protein expression level, which could be varied by tissue regions or imaging depth, and fluorophore brightness.

We also validated the unmixing performance of PICASSO in imaging three spatially overlapping proteins. A mouse brain slice was stained with three preformed antibody complexes against PV, NeuN, and GFAP, conjugated with CF488A, ATTO514, and ATTO532, respectively. The slice was simultaneously stained with guinea pig anti-NeuN antibody and mouse anti-GFAP antibody, and then with CF660R-conjugated secondary antibody against guinea pig and CF405S-conjugated secondary antibody against mouse. By using a 488-nm excitation laser, we acquired three mixed images of PV, NeuN, and GFAP at three detection ranges as shown in IMG1, IMG2, and IMG3 (first raw of Fig. 3k; see Fig. 3a for the detection channels used). We then unmixed the mixed images via PICASSO (second raw of Fig. 3k). We also acquired ground-truth images of NeuN and GFAP by using 405 and 640-nm lasers (Fig. 3l). The unmixed images matched with the ground-truth images.

In all forms of unmixing techniques, including both reference-based and non-reference-based techniques, Poisson noises contained in the mixed images are not properly unmixed, thereby limiting the perceptual quality of the unmixed images^15^. Fortunately, we confirmed that the noise in the unmixed images can be reduced by averaging mixed images (Supplementary Fig. 15). Acquiring images of the same field of view twice and averaging the two images resulted in images with reduced noise.

### Demonstration of PICASSO with multiple excitation lasers

**Fig. 4:**
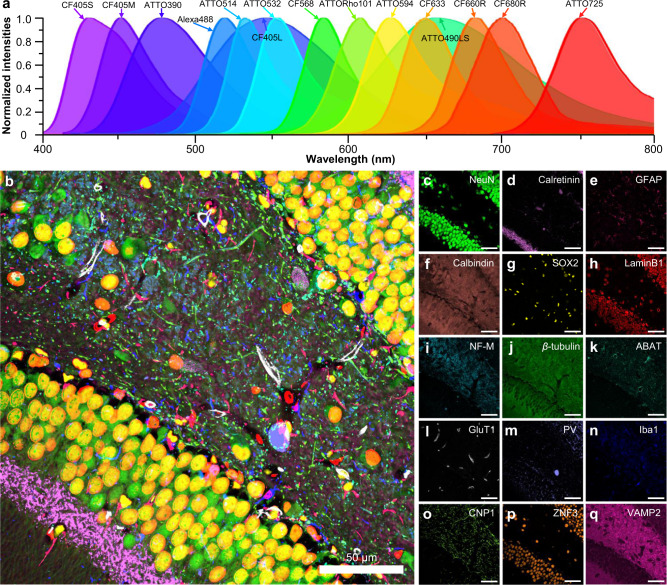
Fifteen-color multiplexed imaging of the mouse brain via PICASSO. **a** Emission spectra of the 15 fluorophores used. **b**–**q** Fifteen-color multiplexed imaging. Fifteen images were acquired at 15 detection channels and blindly unmixed via PICASSO. **b** Fifteen-color multiplexed imaging of the dentate gyrus of the mouse hippocampus. Target proteins are listed in **c**–**q**. **c**–**q** Single-channel images of **b**. The contrast of each channel was adjusted to clearly show all channels in **b**. In **c**–**q**, images were displayed without any contrast adjustments or thresholding. All scale bars; 50 μm.

We then implemented PICASSO with multiple excitation lasers to demonstrate 15-color multiplexed imaging. To achieve this, three spectrally overlapping fluorophores were used for the 405-, 488-, 561-, and 640-nm excitation lasers. The fluorophores used were CF405S, CF405M, and ATTO390 for a 405-nm laser, Alexa Fluor 488, ATTO514, and ATTO532 for a 488-nm laser, CF568, ATTORho101, and ATTO594 for a 561-nm laser, and CF633, CF660R, and CF680R for a 640-nm laser. For the 405- and 488-nm lasers, large Stokes shift fluorophores (CF405L and ATTO490LS) were used alongside the spectrally overlapping fluorophores. One more infrared fluorophore (ATTO725) was used for a 730-nm excitation laser; the total number of fluorophores was 15 (see Fig. 4a for their emission spectra; see Supplementary Fig. 16 for their excitation spectra; and see Supplementary Data 2 for the complete list of the fluorophores). Using these 15 fluorophores, we demonstrated 15-color multiplexed imaging of the mouse hippocampus in which the expressions of these 15 proteins were spatially overlapped, including cell-type markers, subcellular structures, and synaptic proteins, as shown in Fig. 4b–q (see Supplementary Fig. 17 for more 15-color imaging demonstrations). As PICASSO can reliably unmix more than five fluorophores, as shown in Fig. 2h, i and Supplementary Fig. 7, the simultaneous imaging of more than 27 fluorophores, including large Stokes shift fluorophores, would be achievable with five excitation lasers.

### Multiplexed large-area imaging via PICASSO

**Fig. 5:**
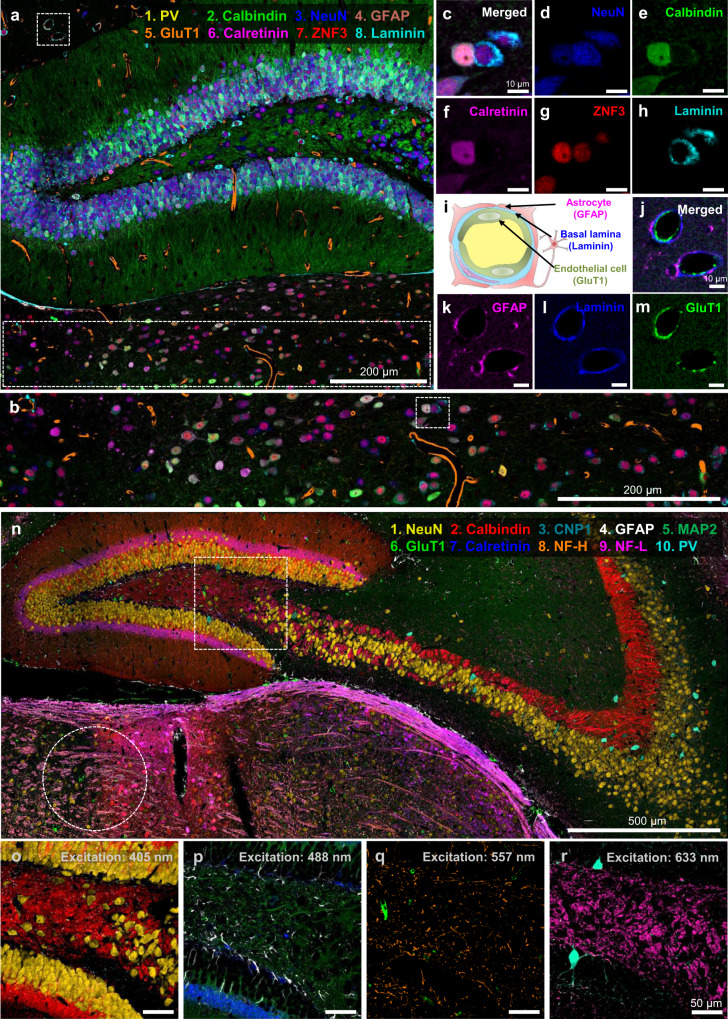
Multiplexed imaging of the mouse brain via PICASSO with multiple excitation lasers. **a** Eight-color multiplexed imaging of the dentate gyrus of the mouse hippocampus. **b** Magnified view of the dotted boxed region at the bottom of **a**. **c**–**h** Magnified view of the dotted boxed region in **b** for individual labeled proteins. **i** Structure of the blood–brain barrier (BBB). **j**–**m** Magnified view of the dotted boxed region at the top left of **a**, clearly showing the cellular structures of the BBB. **n** Ten-color multiplexed imaging of the mouse hippocampus. **o**–**r** Magnified view of the dotted boxed region of **n**. Overlays of two or three channels acquired with a single excitation laser.

We then tested PICASSO’s performance in terms of mosaic imaging, which is required for large-volume imaging. A mouse brain slice was stained with eight preformed antibody complexes against PV, calbindin, NeuN, GFAP, GluT1, zinc finger protein 3 (ZNF3), laminin, and calretinin (see Supplementary Movie 1 for the sample preparation procedure). Mixed images were then acquired at eight detection channels and then unmixed via PICASSO. An eight-color mosaic image was acquired over a millimeter with a lateral resolution of 250 nm and then unmixed via PICASSO (Fig. 5a and see Supplementary Fig. 18 for an enlarged image). The resulting image showed heterogeneous protein expression in the thalamus, as shown in Fig. 5b. For example, when the protein expression levels of the two cells in the dotted box in Fig. 5b were compared, the left cell highly expressed calbindin, calretinin, and ZNF3; conversely, the right cell highly expressed NeuN and laminin (Fig. 5c–h). The eight-color imaging also showed the cellular organization of the blood-brain barrier (BBB) from the same specimen (Fig. 5i–m). As previously reported^16^, endothelial cells were surrounded by a basal lamina and then wrapped by the end-feet of astrocytes in the BBB (Fig. 5i). We compared the spatial expression patterns of five proteins (calbindin, calretinin, ZNF3, NeuN, and PV) with literature reports^17–20^ and databases (rows 1–4 of Supplementary Table 3), and the protein expression patterns we observed matched these reports (see Supplementary Note 3 for details and Supplementary Fig. 19 for individual images of these five proteins).

We then used PICASSO to acquire 10-color multiplexed images of the brain over a large area. A mouse brain slice was stained with ten preformed antibody complexes against NeuN, CNP1, calbindin, calretinin, GFAP, microtubule-associated protein 2 (MAP2), neurofilament-H (NF-H), GluT1, neurofilament-L (NF-L), and PV. Ten images were then acquired at ten detection channels and unmixed using PICASSO. As a result, the ten proteins were successfully visualized over a few millimeters (Fig. 5n–r and see Supplementary Fig. 20 for an enlarged image). The lateral posterior nucleus of the thalamus (the region in the right half of the dotted circle in Fig. 5n) showed high calbindin and calretinin expression (see Supplementary Fig. 21 for details), matching the results reported in the various databases (row 1 of Supplementary Table 3) and the literature^18^. The use of PICASSO is therefore not limited to multiplexed immunostaining; we showed that PICASSO can be applied to multiplexed mRNA fluorescence in-situ hybridization (FISH) (Supplementary Fig. 22a–r), the simultaneous imaging of proteins and mRNA (Supplementary Fig. 22s–v), the super-resolution imaging of tissue via physical expansion^21^ (Supplementary Fig. 23a, b), and tissue clearing^22^ (Supplementary Fig. 23c, d) to improve multiplexing capabilities.

In the following step, we investigated whether PICASSO could be used to uncover the molecular heterogeneity of the mouse brain. We imaged 11 proteins and DAPI in the thalamus of a mouse brain slice (corresponding to slide 74 of the Allen Brain Reference Atlas P56 mouse coronal sections). The 11 proteins included three cell-type markers, which were NeuN (neuron marker), S100B (astrocyte marker), and G protein-coupled receptor 17 (GPR17, oligodendrocyte marker). The remaining eight proteins were PV, necab2, sox2, rap1gap, calretinin, calbindin, ZNF3, and 4-aminobutyrate aminotransferase (ABAT). The DAPI signal identified a total of 7759 thalamus cells in the thalamus, of which 3846 were neurons (NeuN positive), 2567 were astrocytes (S100B positive), and 274 were oligodendrocytes (GPR17 positive) (Supplementary Fig. 24a). Oligodendrocytes were found mostly in the medial habenula (MH) and paraventricular nucleus of the thalamus (PVT). Astrocytes and neurons were found throughout the thalamus. Of the 3846 neurons, 241 expressed PV, indicating that they were GABAergic interneurons. These cells were found in the reticular nucleus of the thalamus (RT), which is consistent with the previous reports^23^. Thereafter, we looked at the expression of seven proteins (excluding PV) in the 3846 neurons. Neurons expressing necab2, sox2, rap1gap, ZNF3, and ABAT were found in the greatest number throughout the thalamus; however, calretinin and calbindin, which are both calcium-binding proteins, were expressed in half of the neurons in the MH, PVT, intermediodorsal nucleus of the thalamus, and medial part of the mediodorsal nucleus of the thalamus. In the RT, the majority of cells did not express sox2, rap1gap, calretinin, and calbindin; instead, they expressed only ZNF3 and ABAT (Supplementary Fig. 24b−i).

### Multiplexed 3D imaging and 10-color imaging with a bandpass filter-based microscope

**Fig. 6:**
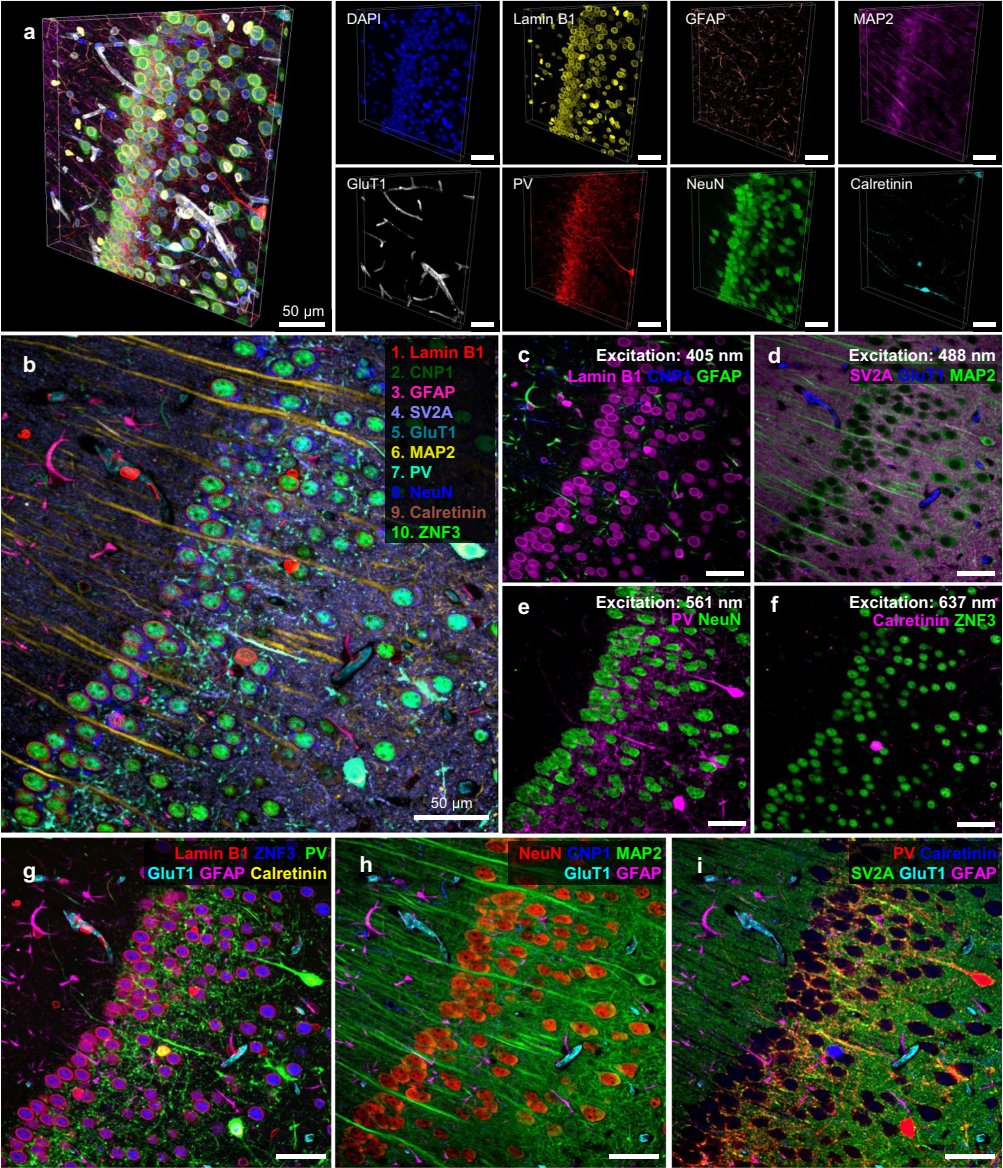
Three-dimensional multiplexed imaging via PICASSO and the use of PICASSO with microscopy equipped with bandpass filters. **a** Three-dimensional view of a *z*-stack image acquired from a mouse brain slice that was stained, imaged, and unmixed by PICASSO. Left: Overlay of the eight channels. Right: Single-channel images. **b** Demonstration of the 10-color multiplexed imaging of the mouse hippocampus CA1 via PICASSO with a microscope equipped with bandpass filters. The images were acquired using a confocal microscope equipped with eight bandpass filters and four excitation lasers. **c**–**f** Two or three channels of the image shown in **b** acquired by one of the four excitation lasers. The wavelengths of the excitation lasers were **c** 405 nm, **d** 488 nm, **e** 561 nm, and **f** 637 nm. **g**–**i** Overlay of five or six channels chosen from the image shown in **b** to clearly visualize different sets of biologically relevant channels together. Different display colors were used for proteins in each image to maximize visibility. **a**–**i** All primary antibodies were rabbit antibodies. All scale bars; 50 μm.

We attempted three demonstrations of PICASSO in order to maximize the demonstration of its potential. First, PICASSO enabled the 3D multiplexed imaging of a mouse brain slice. We found that the emission spectra of fluorophores inside brain slices depended on the imaging depth, in addition to the subregions of the brain (Supplementary Fig. 25). Such imaging depth-dependent emission spectra could be attributed to the wavelength-dependent absorption of light by the specimens^14,24^. We applied the PICASSO unmixing algorithm to each image in a z-stack image set and enabled 3D multiplexed imaging as shown in Fig. 6a (see Supplementary Movie 2 for 3D video; see Supplementary Fig. 26a for individual channels; and see Supplementary Fig. 26b, c for more 3D imaging demonstrations). Second, PICASSO successfully unmixed the signals of spectrally overlapping fluorescent proteins and organic fluorophores (Supplementary Fig. 27). Lastly, 10-color multiplexed imaging was achieved via PICASSO with a microscope equipped with emission bandpass filters that did not have a spectral imaging capability (Fig. 6b). Microscopy systems equipped with spectral detectors are still not commonly available in most research laboratories and hospitals; instead, most microscopy systems use bandpass filters to detect fluorescence signals within specific spectral ranges. Like most bandpass filter-based microscopes, our equipment was able to accommodate up to eight filters, and so we used two filters twice for 10-color imaging (see Supplementary Table 4 for details regarding the filters and imaging conditions). A mouse brain slice was stained with ten antibody complexes and imaged with a microscope equipped with eight bandpass filters; the signals were unmixed by PICASSO (Fig. 6b–f). By using this instrumental setting, a 10-color multiplexed image with a lateral resolution of 250 nm over a field of view of 320 × 320 μm was acquired in less than six seconds (with a ×40 NA1.15 objective; see Supplementary Movie 3). At this speed, the imaging of a 1-mm^2^ field of view would take approximately one minute. Five or six channels were selected and overlaid to better visualize the biological contexts (Fig. 6g–i).

We also tested whether PICASSO works on diverse specimens, especially in clinical samples, by using a tissue microarray containing FFPE specimens from 12 human organs. We chose keratin 19, histone H3, vimentin, and cytochrome c oxidase subunit 4 (COX IV) as target proteins, as they are related to cancers and used as cancer markers^25–28^. We first attempted to image keratin 19 and histone H3 with two spectrally overlapping fluorophores. Keratin 19 and histone H3 are localized in different cellular compartments (the cytoplasm and nucleoplasm, respectively; see rows 5 and 6 of Supplementary Table 3); their expression patterns should therefore be spatially separated if their signals are successfully unmixed. The obtained images clearly show the spatial separation of the two proteins (Supplementary Fig. 28a–l), indicating that PICASSO successfully separated the signals of two spectrally overlapping fluorophores in clinical specimens. We then attempted the simultaneous imaging of keratin 19, histone H3, vimentin, and COX IV in clinical specimens. The simultaneous visualization of keratin and vimentin in a single specimen is clinically significant because the vimentin/keratin ratio is related to epithelial-mesenchymal transition status^29^. The four proteins were successfully visualized using two pairs of spectrally overlapping fluorophores and two excitation lasers in four different organs (Supplementary Fig. 28m–p). Autofluorescence is sometimes a severe issue in the imagining of clinical specimens. To remove this autofluorescence from immunostaining signals, its emission profiles need to be measured in each specimen before the staining and then used as a reference profile for unmixing. PICASSO was able to separate autofluorescence from true staining signals without any autofluorescence measurement (Supplementary Fig. 29).

### Cyclic-PICASSO

**Fig. 7:**
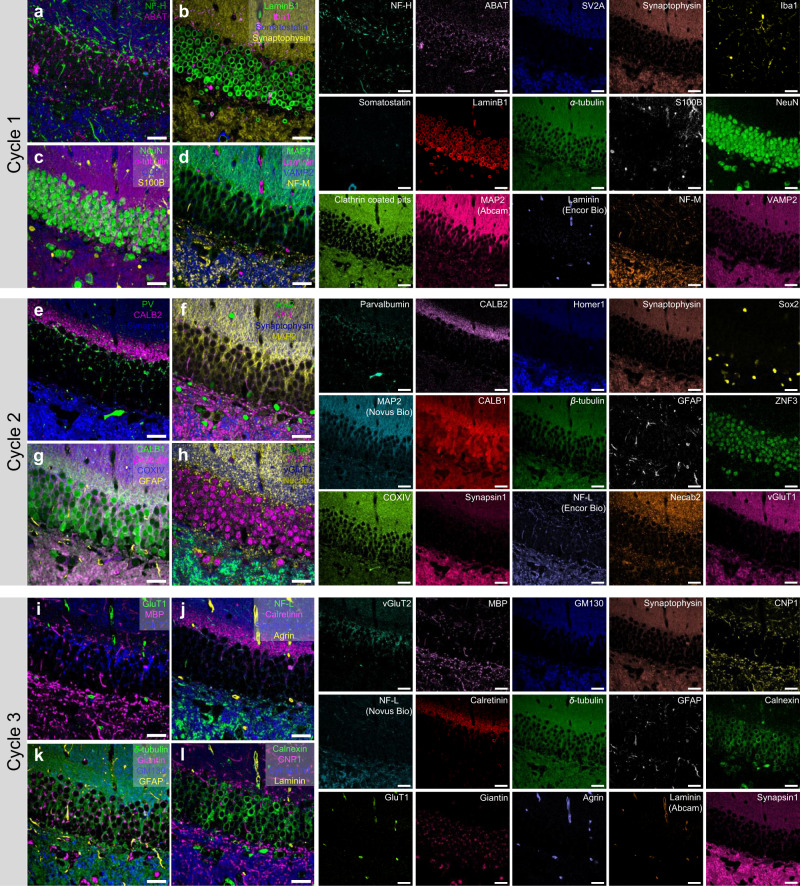
Forty-five-color multiplexed imaging via Cyclic-PICASSO. **a**–**d** Overlay of three or four channels chosen from 15-color images of cycle 1 imaging to visualize different sets of biologically relevant channels together. Individual images were presented on the right side of **a**–**d**. **e**–**h** Overlay of the three or four channels chosen from the 15-color images of cycle 2 imaging. The individual images were presented on the right side of **e**–**h**. **i**–**l** Overlay of the three or four channels chosen from 15-color images of cycle 3 imaging. The individual images were presented on the right side of **i**–**l**. Synaptophysin in each cycle was used as a fiducial marker. All scale bars; 30 μm.

PICASSO can also be combined with cyclic immunofluorescence techniques to image an unlimited number of proteins with a reduced number of cycles. For each staining and imaging round, a brain slice was stained with 15 different preformed antibody complexes that were labeled with 15 spectrally overlapping fluorophores and then it was imaged and bleached using four lasers (405, 488, 561, and 640 nm). By so doing, we achieved 45-color multiplexed imaging of the dentate gyrus of a mouse brain. PICASSO unmixed the signals of 15 spectrally overlapping fluorophores in multiple cycles, indicating that it can be combined with cyclic immunofluorescence techniques to increase the number of fluorophores used simultaneously in a single cycle (Fig. 7).

## Discussion

We herein proposed PICASSO, which enables the simultaneous imaging of spatially overlapping proteins labeled with spectrally overlapping fluorophores without any reference spectra. Through simulation, we showed that PICASSO can reliably unmix five spectrally overlapping fluorophores that can be simultaneously excited by a single excitation laser, even with <10-nm of spectral separation; it is also not limited to only five fluorophores. We successfully demonstrated 15-color multiplexed imaging of proteins whose expression patterns overlapped using multiple excitation lasers. PICASSO does not need reference spectra; hence, 15 or more proteins can be simultaneously imaged without the time-consuming, complicated, and sometimes impossible reference measurement processes. PICASSO only needs one image per fluorophore, minimizing the number of required detection channels. As PICASSO needs only a small number of images and its requirements regarding detection channels are not strict, it can be implemented with bandpass filter-based microscopy, which is currently available to most biology laboratories and hospitals. The use of a small number of images has additional advantages in terms of signal-to-noise ratio, as the number of photons collected per channel can be maximized for the simultaneous imaging of a given number of fluorophores within a given spectral range^2^. As PICASSO does not require repeated staining and imaging processes up to 15 colors, multiplexed 3D imaging can be easily performed without a complicated 3D registration process. We showed that PICASSO can increase the multiplexing capability of mRNA imaging, the co-imaging of mRNA and proteins, expansion microscopy, tissue clearing, and even cyclic staining. PICASSO shares the practical limitations of spectral unmixing, including susceptibility to noise and difficulty in distinguishing spectrally identical fluorophores; such limitations could be resolved by combining PICASSO with excitation unmixing.

PICASSO is a versatile tool for the multiplexed biomolecule imaging of cultured cells, tissue slices, and clinical specimens. It is suitable for high-throughput protein imaging, as antibodies can be simultaneously applied to specimens during the staining process and a minimal number of collected images are required. In addition, PICASSO does not require complicated optics or spectral detectors; it can instead be implemented with a simple imaging system consisting of objectives, light sources such as a lamp or LED, a camera, and excitation/emission bandpass filters (Supplementary Fig. 30). For more than 10-color imaging, custom multi-band pass filters could be used for such microscopy setups. PICASSO can be combined with live imaging^3^, tissue clearing^22,30^, or tissue expansion^21,31–33^ techniques to achieve multiplexed 3D super-resolution imaging or multiplexed whole-organ imaging. PICASSO can also be combined with cyclic immunofluorescence imaging techniques based on various mechanisms, such as cyclic DNA hybridization, photo-bleaching of fluorophores, chemical bleaching of fluorophores, and antibody stripping, without any significant change^34–42^. Once combined, fewer cycles are then required to image a given number of proteins, greatly reducing the time and complexity of the whole imaging process. As PICASSO is a strategy for distinguishing fluorophores, it could also be used to improve the multiplexing capabilities of mRNA imaging^43–46^, bioassays^47^, and cell tracing^48^. It could also be combined with fluorescent barcoding techniques to increase the amount of information that a single barcode can encode^49–52^.

We anticipate that PICASSO will be useful for a broad range of applications for which biomolecules’ spatial information is important. PICASSO would be useful for revealing the cellular heterogeneities of tumor microenvironments, especially the heterogeneous populations of immune cells, which are closely related to cancer prognoses^53^ and the efficacy of cancer therapies^54^. In addition, the multi-scale 3D atlases of biomolecules^55–58^ have changed our understanding of biology^59^. In constructing these atlases, multiplexed fluorescence imaging is one of the major tools to map the spatial distribution of biomolecules. PICASSO is expected to enable the simultaneous 3D visualization of larger groups of biomolecules, providing information about the co-localization of either different proteins or mRNA molecules or how their expressions are correlated.

## Methods

### Ethical statement

All of the following procedures involving animals were approved by the Sungkyunkwan University Institutional Animal Care and Use Committee (SKKU-IACUC, approved protocol number, SKKUIACUC-17-10-8-1), and the Korea Advanced Institute of Science and Technology Institutional Animal Care and Use Committee (KAIST-IACUC, approved protocol number, KA2020-48).

### Antibodies and chemicals

All the antibodies and chemicals used in this study are listed in Supplementary Data 1 and 3.

### Cell culture and fixation

BS-C-1, HeLa, and NIH-3T3 cells were purchased from the Korean Cell Line Bank and cultured in a Nunc Lab-Tek II chambered #1.5 coverglass. The BS-C-1 cells were cultured in Minimum Essential Medium (MEM) supplemented with 10% fetal bovine serum (FBS), 1% penicillin–streptomycin, and 1% sodium pyruvate. The HeLa cells were cultured in Dulbecco’s modified Eagles’ medium (DMEM) supplemented with 10% FBS and 1% penicillin–streptomycin. The NIH-3T3 cells were cultured in DMEM supplemented with 10% bovine calf serum (BCS) and 1% penicillin–streptomycin. All cells were incubated at 37 °C in 5% CO_2_. For the fixation, the cells were washed with 1× phosphate-buffered saline (PBS) three times and fixed with 4% paraformaldehyde (PFA) in 1× PBS for 10 min, then washed with 1× PBS three times. The cells were then incubated with 0.1 M glycine in 1× PBS for 10 min and washed with 1× PBS three times.

### Mouse brain perfusion and slicing

All of the following procedures involving animals were approved by the Sungkyunkwan University Institutional Animal Care and Use Committee (SKKU-IACUC, approved protocol number, SKKUIACUC-17-10-8-1), and the Korea Advanced Institute of Science and Technology Institutional Animal Care and Use Committee (KAIST-IACUC, approved protocol number, KA2020-48). C57BL/6 J and Thy1-YFP male mice aged 8–14 weeks were used. The mice were raised in ventilated cages under a 12-h light/dark cycle at 20–24 °C with 40–60% humidity. The mice were anesthetized with isoflurane and transcardially perfused with ice-cold 4% PFA in 1× PBS. Brains were harvested and stored in the same solution at 4 °C for 2 h before being sliced into 150 μm-thick slices on a vibratome (Leica VT1000S). The slices were stored in 0.1 M glycine and 0.01% sodium azide in 1× PBS at 4 °C until use.

### Conjugation of Fab fragment antibodies with fluorophores

For the Alexa and CF fluorophores, 10 μL of 1 M sodium bicarbonate (pH 8.3) and a 9-fold molar excess of succinimidyl ester-fluorophore stock in dimethyl sulfoxide (DMSO) were added to 90 μL of unconjugated antibody solutions. For the ATTO fluorophores, 10 μL of 1 M sodium bicarbonate (pH 8.3) and a threefold molar excess of succinimidyl ester-fluorophore stock in dimethyl sulfoxide (DMSO) were added to 90 μL of unconjugated antibody solutions. The fluorophore-antibody solutions were incubated at RT for 1 h in darkness. To purify the labeled antibodies, NAP-5 gel filtration columns were used. The columns were equilibrated with 10 mL of 1× PBS. 100 μL of the reacted solutions were loaded into the columns. The eluates containing fluorophore-conjugated antibodies were collected after loading 900 μL of 1× PBS into the columns. Then, the eluates were concentrated using centrifugal filters with a molecular weight cut-off (MWCO) of 30,000.

### Preparation of preformed antibody complexes

Preformed antibody complexes were prepared according to the original primary antibody-Fab complex formation protocol^13^. Briefly, 1× PBS, a solution containing a fluorophore-conjugated Fab fragment antibody, and a solution containing a primary antibody were mixed at a volume ratio of 10:2:1 and then incubated for 10 min at RT in darkness. A fivefold excess volume of a blocking buffer (10% normal rabbit serum, 0.2% Triton X-100, 1× PBS) was then added to the solution and incubated for 10 min at RT in darkness with gentle shaking. Then, the preformed antibody complexes against different targets were mixed together, diluted in the blocking buffer, and then used for staining. The antibody concentration was adjusted according to the manufacturer’s instructions for each primary antibody. When the fluorescence intensity of the specific antibody was not high enough, we doubled the concentration to the recommended antibody concentration, as provided by the vendors of the primary antibodies. The 28 fluorophores tested with the preformed antibody complex are listed in Supplementary Table 2.

### Staining of cells and mouse brain slices with preformed antibody complexes

All of the following steps were performed at RT. For permeabilization and blocking, cells were incubated in a blocking buffer (10% normal rabbit serum, 0.2% Triton X-100, 1× PBS) for 30 min. The cells were then stained with a preformed antibody complex mixture for 30 min and then washed three times with the blocking buffer. For the staining of mouse brain slices, blocking, staining, and washing steps were identical to the cultured cell protocol except for the incubation time (blocking: 1.5 h, staining: overnight, washing: 30 min).

### Validation of the staining using a preformed antibody complex

For the validation study shown in Supplementary Fig. 9, mouse brain slices were blocked in a blocking buffer (10% normal rabbit serum, 0.2% Triton X-100, 1× PBS). The slice was then incubated overnight in a preformed rabbit antibody solution diluted at 1:500 at 4 °C and then washed three times with a blocking buffer. The slice was then stained overnight with a 1:500 chicken primary antibody in an NGS buffer (5% normal goat serum, 0.2% Triton X-100, 1× PBS) at 4 °C and then washed three times with the NGS buffer. The slice was then stained overnight with an anti-chicken secondary antibody diluted to 1:250 in the NGS buffer at 4 °C and then washed with the NGS buffer three times. For the staining of BS-C-1 cell, blocking, staining, and washing steps were identical to the cultured cell protocol except for the incubation time (blocking: 30 min, staining: 30 min, washing: immediately).

### Study on the crosstalk among 13 preformed antibody complexes

For the study shown in Supplementary Fig. 10b, a mouse brain slice was stained with an anti-GFAP primary antibody and then labeled with CF405S conjugated Fab fragments secondary antibody. Anti-GFAP preformed antibody complex bearing CF488A and 12 different preformed antibody complexes (unlabeled) against NeuN, ABAT, CALB1, CALB2, CCP, CNP1, laminB1, MBP, PV, SV2A, VAMP2, and ZNF3 were simultaneously treated to the sample. The experiment process of Supplementary Fig. 10c–n was the same as Supplementary Fig. 10b, except for the target proteins.

### Validation of unmixing via MI minimization

To compare the unmixing accuracies of linear unmixing and PICASSO, as shown in Fig. 2d–f, 25 mixed images containing 32 channels were synthesized using four source images of a mouse brain slice (i.e., CNP1, NeuN, GluT1, and GFAP) and the 25 measured reference spectra. In this process, 32-channel spectral imaging (400–720 nm) was performed in five subregions (i.e., CA1, CA3, DG, the thalamus, and the cortex) of four mouse brains, each of which was stained with a preformed antibody complex against NeuN and visualized by CF488A, ATTO488, ATTO514, and ATTO532, respectively. Then, emission spectra were extracted from the top 1% of pixels (in terms of brightness) of each 32-channel spectral image to obtain the measured reference spectra. The 25 synthetic images were unmixed using the average of the measured reference spectra for linear unmixing. For PICASSO, four channels (channel 1: 510–520 nm; channel 2: 520–530 nm; channel 3: 530–540 nm; channel 4: 540–550 nm) were selected from the 32-channel synthetic images.

For the five-color unmixing simulation shown in Fig. 2h, i and Supplementary Fig. 7, a five-channel mixed image was synthesized using five source images of a mouse brain slice (i.e., NeuN, CNP1, GluT1, PV, and GFAP) and a mixing matrix. The mixing matrix was generated by considering emission spectra in five detection channels (channel 1: 645–655 nm; channel 2: 663–673 nm; channel 3: 677.8–687.8 nm; channel 4: 695.9–705.9 nm; channel 5: 714–724 nm) and the absorption spectra of five fluorophores (i.e., CF633, Alexa Fluor 647, CF660R, CF680R, and ATTO700) at 640 nm excitation. Then, Gaussian additive noise was added to the five-channel synthetic image. The level of the added noise was determined by measuring the pixel intensity profiles of the 0.1 mM CF633 fluorophore in our microscope system (Nikon C2 Plus), followed by calculating the Gaussian distribution of the noise. Lastly, the five-channel synthetic image with Gaussian noise was fed into the PICASSO algorithm.

For the experimental validation of unmixing via MI minimization shown in Fig. 3, mouse brain slices were blocked in a blocking buffer (10% normal rabbit serum, 0.2% Triton X-100, 1× PBS). For Fig. 3c–j, a mouse brain slice was incubated overnight with a mixture of preformed rabbit anti-GluT1 antibody (labeled with CF488A and CF568) and preformed rabbit anti-GFAP antibody (labeled with ATTO514 and CF405S) diluted to 1:1000 in the blocking buffer. For Fig. 3k, l, a mouse brain slice was incubated overnight with a mixture of preformed rabbit anti-PV antibody (labeled with CF488A), preformed rabbit anti-NeuN antibody (labeled with ATTO514), preformed rabbit anti-GFAP antibody (labeled with ATTO532), guinea pig anti-NeuN antibody, and mouse anti-GFAP antibody diluted to 1:1000 in the blocking buffer. The sample was washed with the blocking buffer three times and stained overnight with a CF405S-labeled anti-mouse secondary antibody, and CF660R-labeled anti-guinea pig secondary antibody diluted to 1:500 in the blocking buffer.

### FFPE samples preparation and staining

The following experiments using human samples were all approved by the Korea Advanced Institute of Science and Technology Institutional Review Board (KAIST IRB). The normal human multi-organ tissue microarray (TMA) used in Supplementary Figs. 28 and 29 were purchased from Novus Biologicals (NBP2-30189). For the FFPE clinical samples, the sample slides were dried for 1 h in an oven at 60 °C. The slides were then deparaffinized in xylene twice for 5 min each time. For hydration, the slides were placed in a series of solutions—namely 100% ethanol (EtOH) twice, 95% EtOH, 80% EtOH, and deionized water at RT—for 3 min each. The slides used in this study were processed with a heat-induced epitope retrieval (HIER) procedure before the staining^60^. Briefly, the slides were placed in a retrieval solution (10 mM sodium citrate, 0.05% Tween 20, pH 6.0) for 30 min at 95–100 °C and allowed to cool for 20 min in 1× PBS. The slides were blocked with a blocking buffer (10% normal rabbit serum, 0.2% Triton X-100, 1× PBS) for 3 h at RT, followed by overnight incubation with preformed antibody complexes at RT, before being washed three times with the blocking buffer at RT for 30 min each. If the autofluorescence in the specimens is non-negligible, it can be quenched by using commercially available autofluorescence quencher kits (e.g., Vectrolab TrueVIEW or Biotium TrueBlack).

### Hybridization chain reaction (HCR)

For the PICASSO imaging of two mRNA molecules shown in Supplementary Fig. 22a–r, HCR (Molecular Instruments) was conducted on NIH-3T3 cells as instructed by the manufacturer’s user manual. Briefly, fixed NIH-3T3 cells were permeabilized overnight with 70% EtOH and stored at –20 °C until their use. The permeabilized cells were rinsed with 2× saline sodium citrate (SSC) and incubated with an HCR probe hybridization buffer (Molecular Instruments) for 30 min at 37 °C. Then, the cells were incubated with a probe solution (HCR probes diluted to 4 nM in the HCR probe hybridization buffer) for 12–16 h at 37 °C. After the probe hybridization, the cells were washed four times with an HCR probe wash buffer (Molecular Instruments) for 5 min at 37 °C and then washed two more times with 5× SSCT (5× SSC, 0.1% Tween 20) for 5 min at RT. The cells were incubated with an HCR amplification buffer (Molecular Instruments) for 30 min at RT and then with an amplification solution for 12–16 h at RT in darkness. The amplification solution was prepared by the snap-cooling of HCR hairpin amplifiers H1 and H2, heating the amplifier hairpins at 95 °C for 90 s and then cooling them down to RT in darkness for 30 min. The snap-cooled hairpins were then diluted to 6 nM in the HCR amplification buffer to make the amplification solution. After the amplification, the cells were washed five times with 5× SSCT for 5 min at RT. The cells were then stained with 4′,6-diamidino-2-phenylindole (DAPI), and rinsed with 1× PBS.

### Custom dye conjugation to HCR hairpin amplifiers

The unconjugated hairpin amplifier was purchased from Molecular Instruments. First, 75 μL of the labeling buffer (0.1 M sodium tetraborate, pH 8.5) and 5 μL of deionized water were added to a reaction vial. Then, 10 μL of 100 μM unlabeled hairpins (H1 and H2 were conjugated separately) and 10 μL of 10 mg/mL ATTORho101-NHS-ester were added to the reaction vial and allowed to react for 6 h. After the reaction, 10 μL of 3 M sodium chloride (NaCl) and 250 μL of –20 °C pure ethyl alcohol were added to the reaction vial and then stored at –20 °C for 30 min. The reaction vial was then centrifuged at 12,000 g for 30 min. After the centrifugation, the supernatant was carefully removed, and the DNA pallet on the bottom of the vial was rinsed with ice-cold 70% ethyl alcohol twice. The pellet was then dried in darkness overnight and re-dissolved in 1× PBS or storage buffer (150 mM NaCl and 100 mM sodium phosphate dibasic).

### Simultaneous imaging of mRNA and protein

For the simultaneous imaging of the mRNA and protein shown in Supplementary Fig. 22s–v, RNAscope (Advanced Cell Diagnostics) was conducted on NIH-3T3 cells as instructed by the manufacturer’s user manual. After the RNAscope labeling of the *Gapdh* mRNA of the cultured cells, the cells were stained with a preformed rabbit anti-vimentin complex as described in ‘Staining of cells and mouse brain slices with preformed antibody complexes.’

### Single-cell protein expression analysis

Supplementary Fig. 24 shows single-cell protein expression analysis in a mouse brain slice through 12-color imaging. A 150-μm-thick mouse brain slice was stained with 11 preformed antibody complexes: four antibodies (anti-NeuN, PV, S100B, and GPR17) as the cell type markers, seven antibodies (anti-Necab2, Sox2, Rap1gap, CALB2, CALB1, ZNF3, and ABAT) for quantification of protein expression, and DAPI for cell segmentation. Next, images of the sample were acquired using a point-scanning confocal microscope (Nikon C2 Plus), and unmixing was performed using the PICASSO algorithm. Laplacian of Gaussian filter and image dilation to the DAPI channel were applied to detect the cells in the image. Then, a watershed algorithm was used to segment the cells. Neurons, astrocytes, and oligodendrocytes were classified based on the expression of cell-type markers (NeuN, PV, S100B, and GPR17). Thereafter, the neurons were classified into each phenotype based on the expression of seven target proteins (Necab2, Sox2, Rap1gap, CALB2, CALB1, ZNF3, and ABAT) within each cell area.

### Cyclic-PICASSO

A 150-μm-thick mouse brain slice was incubated in a blocking buffer (10% normal rabbit serum, 0.2% Triton X-100, 1× PBS) for 1.5 h. For the first staining round, the slice was stained with fifteen preformed antibody complexes against neurofilament-H, ABAT, SV2A, synaptophysin, Iba1, somatostatin, laminB1, *α*-tubulin, S100B, NeuN, clathrin-coated pits, MAP2, laminin (Encor biotechnology), neurofilament-M and VAMP2 overnight at RT, and then washed three times with the blocking buffer for 30 min. For the second staining round, the slice was stained with 13 preformed antibody complexes (i.e., anti-parvalbumin, CALB2, synapsin1, synaptophysin, neurofilament-L, *β*-tubulin, COXIV, GFAP, CALB1, homer1, ZNF3, necap2, and vGluT1) and two fluorophore-conjugated primary antibodies (i.e., anti-SOX2 and MAP2) overnight at RT, and then washed three times with the blocking buffer for 30 min. For the third staining round, the sample was stained with 11 preformed antibody complexes (i.e., anti-vGluT2, SLC2A1, synaptophysin, calretinin, *δ*-tubulin, GFAP, GM130, CNP1, laminin (Abcam), calnexin and synapsin1), two fluorophore-conjugated primary antibodies (i.e., anti-agrin and neurofilament-L), chicken anti-MBP and guinea pig anti-giantin overnight at RT, and washed three times with the blocking buffer for 30 min. Then, anti-chicken and anti-guinea pig secondary antibodies were treated to the sample overnight at RT, and washed three times with the blocking buffer for 30 min. After every round of imaging, the fluorophores of the stained sample were inactivated by LU-N4 laser units equipped with four standard lasers (405, 488, 561, and 640 nm) with pixel dwell time 0.5 μs and laser power 15 mW. Synaptophysin was applied to the slice for each cycle as a fiducial marker. Detailed information about the imaging is listed in Supplementary Data 2.

### Protein-retention expansion microscopy

Stained brain slices were incubated in acryloyl-X, SE ((6-((acryloyl)amino)hexanoic acid, succinimidyl ester, AcX) diluted to 0.1 mg/ml in 1× PBS overnight at RT and then washed three times for 30 min with 1× PBS. The samples were then incubated twice with a monomer solution (7.5% (w/w) sodium acrylate, 2.5% (w/w) acrylamide, 0.15% (w/w) *N*,*N*′-methylenebisacrylamide (BIS), 1× PBS, 2 M NaCl) at 4 °C for 30 min each time. After incubation, the samples were placed between two cover glasses filled with a gelation buffer (monomer solution, 0.2% (w/w) ammonium persulfate (APS), 0.2% (w/w) tetramethylethylenediamine (TEMED), 0.01% (w/w) 4-hydroxy-2,2,6,6-tetramethylpiperidin-1-oxyl (H-TEMPO)) and incubated at 37 °C for 1.5 h. The gels were treated with proteinase K diluted at 1:100 in a digestion buffer (25 mM ethylendiaminetetraacetic acid (EDTA), 50 mM Tris-HCl (pH 8), 0.5% Triton X-100, 1 M NaCl) at 37 °C overnight with gentle shaking. After digestion, the digested gels were placed in deionized water (DI) with gentle shaking.

### SHIELD

SHIELD (LifeCanvas Technologies) was conducted as instructed by the manufacturer’s user manual. Briefly, 500 μm-thick mouse brain slices were incubated in a SHIELD-OFF solution (a mixture of DI water, SHIELD-Buffer solution, and SHIELD-Epoxy solution at a ratio of 1:1:2) at 4 °C with gentle shaking for 1 day. The samples were transferred to a mixture of SHIELD-ON buffer and SHIELD-Epoxy solution with a ratio of 7:1 and then incubated at 37 °C with gentle shaking for 6 h. Samples were incubated in the SHIELD-ON buffer at 37 °C with gentle shaking overnight. For tissue clearing, samples were incubated in a clearing solution (300 mM sodium dodecyl sulfate, 10 mM boric acid, 100 mM sodium sulfite, pH 9.0) at 37 °C with gentle shaking for 1 day. Cleared samples were imaged in 1× PBS.

### Imaging

In this work, three microscopy systems were used. The first two were point-scanning confocal microscopy systems: Leica SP8 equipped with a white light laser and Nikon C2 Plus equipped with four excitation lasers (405, 488, 561, 640 nm). Even though the Leica microscope had a white light laser and its excitation wavelength was adjustable, we used the same excitation wavelengths as the Nikon system to test the multiplexed imaging ability of PICASSO with two different typical microscopy systems. For the images shown in Fig. 5, 633 or 660 nm was used as an excitation wavelength instead of 640 nm to image red fluorophores with a higher SNR; however, 640 nm can be used to image three proteins labeled with spectrally overlapping fluorophores, as shown in Fig. 4b–q. For spectral imaging, Nikon C2 plus was used again. Nikon C2 plus was used in Fig. 2 and Supplementary Figs. 1, 3, and 25. The third system was a spinning-disk confocal microscopy system, which was Andor Dragonfly equipped with five excitation lasers (405, 488, 561, 637, and 730 nm). All the images were acquired by using a ×40 1.15 NA water-immersion objective. Detailed information about the imaging is listed in Supplementary Data 2. For Supplementary Fig. 14, to minimize the effect of the signal intensity variation in a single field of view (FOV), each of the two droplets was imaged at the center of the FOV and then stitched to generate a mixed image.

### Pre-processing of images for unmixing

We performed pre-processing of images before unmixing the images. We first resized input images to 50% of their original sizes by using the ‘resize’ function of the Fiji image-processing software, with the option ‘average when downsizing’ checked, to reduce the shift of structures contained in different channels caused by chromatic aberration. If the chromatic aberration correction process is introduced, such resizing would not be needed. For mosaic images, the shading and background variations were corrected using BaSiC^61^ and then stitched. The images from the spinning-disk confocal microscope were further processed. First, vignetting in the images was corrected using the image of a fluorescent slide. Second, ring-shaped artifacts in the images, possibly due to the interference of the excitation laser with internal optics of the spinning-disk microscopy system (e.g., two rotating disks with an array of microlenses), were removed by subtracting an image taken without specimens. Third, the pixel shift due to the chromatic aberration was handled via image registration.

### Unmixing

For PICASSO unmixing, we first initialized the solution *X*_(0)_ as the acquired images IMG. Then, we construct a progressive unmixing matrix as follows:

$${P}_{(k)}=\left[\begin{array}{c}\begin{array}{c}\begin{array}{ccc}1 & -\gamma {\alpha }_{1,2(k)} & \ldots \\ -\gamma {\alpha }_{2,1(k)} & 1 & \ddots \\ \begin{array}{c}\vdots \\ -\gamma {\alpha }_{N,1(k)}\end{array} & \begin{array}{c}\ddots \\ \ldots \end{array} & \begin{array}{c}\ddots \\ -\gamma {\alpha }_{N,N-1(k)}\end{array}\end{array}\begin{array}{c}-\gamma {\alpha }_{1,N(k)}\\ \vdots \\ \begin{array}{c}-\gamma {\alpha }_{N-1,N(k)}\\ 1\end{array}\end{array}\end{array}\end{array}\right]$$

where the *k* and *γ* denote the iteration number and the update step size, respectively; *α*_*i*, *j*(*k*)_ was calculated as ${\alpha }_{i,j(k)}={\arg }\mathop{{{{{{\rm{min }}}}}}}\nolimits_{\alpha }{{{{{\rm{I}}}}}}(q({bin}({X}_{j(k)}));q({bin}({X}_{i(k)}-\alpha {X}_{j(k)})))$ using the Nelder–Mead simplex algorithm where q(∙) and *bin*(∙) denote the quantization and pixel binning functions. The parameters *α*_*i*, *j*(*k*)_ were calculated using binned and quantized images, to improve the robustness against noise and the speed of the joint histogram calculation, whereas the progressive unmixing, ${X}_{(k+1)}={P}_{(k)}{X}_{(k)}$, was performed on the full-resolution images without quantization (see Supplementary Note 2 for details).

For linear unmixing with reference spectra, the linear matrix equation IMG = M × *F* was directly set using the acquired 32-channel image IMG and the reference spectra matrix *M*. The equation was solved using QR factorization. For unmixing with phasor analysis, each pixel in the acquired 32-channel image was normalized in the spectral domain and then 32-point discrete Fourier transform was applied in a pixel-wise manner. We used the sixth harmonic (*n* = 6) to map the image to the phasor plane. The contribution from each fluorophore to each pixel was estimated by measuring the relative area of the triangle formed by each pixel and the reference spectra in the phasor plane. For non-reference-based unmixing with NMF, the acquired 32-channel image ${{{{{\rm{IMG}}}}}}\in {{\mathbb{R}}}^{32\times m}$ was unmixed by directly factorizing as IMG = *W* × *H* (${{{{{\rm{W}}}}}}\in {{\mathbb{R}}}^{32\times k}$, ${{{{{\rm{H}}}}}}\in {{\mathbb{R}}}^{k\times m}$), where *m* is the number of pixels of the image and *k* is the number of fluorophores, using NMF with alternating least squares method. All image processing was conducted using custom-written MATLAB codes.

### Statistics and reproducibility

All imaging results (i.e., Fig. 2e, h, i; 3c–l; 4b–q; 5a–h, j–r; 6a–i; 7a–l and Supplementary Fig. 1c–f; 3a, b; 5a–e, h–m; 6a, f–i; 7a–c; 8b–f; 9a–e; 10b–n; 11; 12c, d; 13; 15a–d; 17a–j; 18; 19a–f; 20; 21; 22a–v; 23a–d; 24a–f; 26a–c; 27a–j; 28a–p; 29a, b; 30a, b) were repeated more than three times, respectively, with similar results.

### Reporting summary

Further information on research design is available in the Nature Research Reporting Summary linked to this article.

## Supplementary information

Supplementary Information

Reporting Summary

Description of Additional Supplementary Files

Supplementary Movie 1

Supplementary Movie 2

Supplementary Movie 3

Supplementary Data 1

Supplementary Data 2

Supplementary Data 3

Supplementary Software

## Data Availability

The unmixing data generated in this study have been deposited in the Figshare database 10.6084/m9.figshare.19596682.v1. Source data are provided with this paper.

## Source data

Source Data

## Code availability

The unmixing code written in MATLAB is contained in Supplementary Software along with a demo image and instruction.
