# Supplementary material for: PICASSO allows ultra-multiplexed fluorescence imaging of spatially overlapping proteins without reference spectra measurements
Source: Nat Commun. 2022 May 5;13:2475. doi: 10.1038/s41467-022-30168-z (PMC9072354; doi:10.1038/s41467-022-30168-z)
Supplement: Supplementary file 1 — Supplementary Information [file 41467_2022_30168_MOESM1_ESM.pdf]

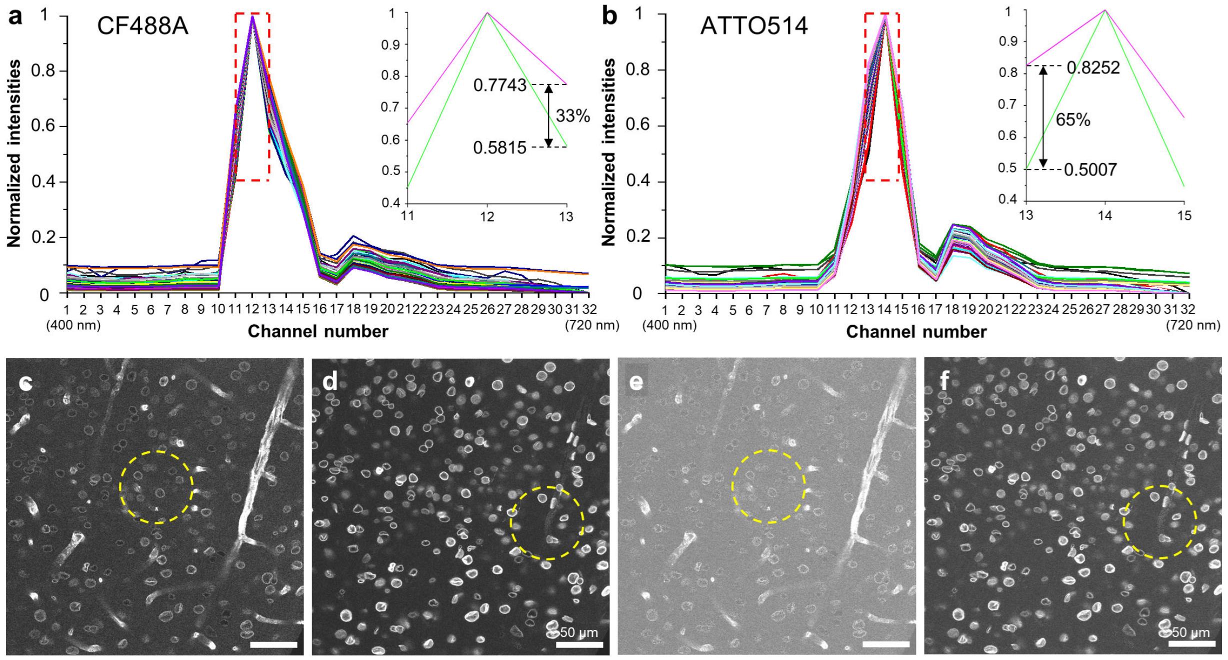

Supplementary Figure 1. **Variations in the emission spectra of fluorophores and their effects on the unmixing performance of linear unmixing and phasor analysis**. (**a**, **b**) Normalized emission spectra of CF488A and ATTO514 measured across multiple subregions of the mouse brain slices. Images of mouse brain slices that were stained either with a CF488A-conjugated antibody or ATTO514-conjugated antibody were acquired using a 32-channel spectral detector and emission spectra were measured from the images (64 spectra for CF488A and 71 spectra for ATTO514). To exclude the effect of autofluorescence or background signals, pixels with the top 1% brightness were selected from each image set, after which the emission spectra were measured. Insets: magnified views of the red boxed regions showing the uppermost (magenta) and lowermost spectrum (green). Note that the CF488A and ATTO514 spectra show a high level of variation between the measurements. (**a**) Normalized emission spectra of CF488A. (**b**) Normalized emission spectra of ATTO514. (**c**–**f**) Effects of the variations in the emission spectra on the performance of linear unmixing and phasor analysis. A mouse brain slice was stained with a CF488A-conjugated antibody against GluT1 and an ATTO514-conjugated antibody against lamin B1 and then imaged using a 32-ch spectral detector. For unmixing, one of the reference spectra shown in **a** and **b** were used. (**c**, **d**) Unmixed via linear unmixing. (**e**, **f**) Unmixed via phasor analysis. Due to variations in the spectra, two fluorophore signals were not completely unmixed. In **c** and **e**, only GluT1 should be visible, but lamin B1 is also visible (yellow dotted circle). In **d** and **f**, only lamin B1 should be visible, but GluT1 is also visible (yellow dotted circle).

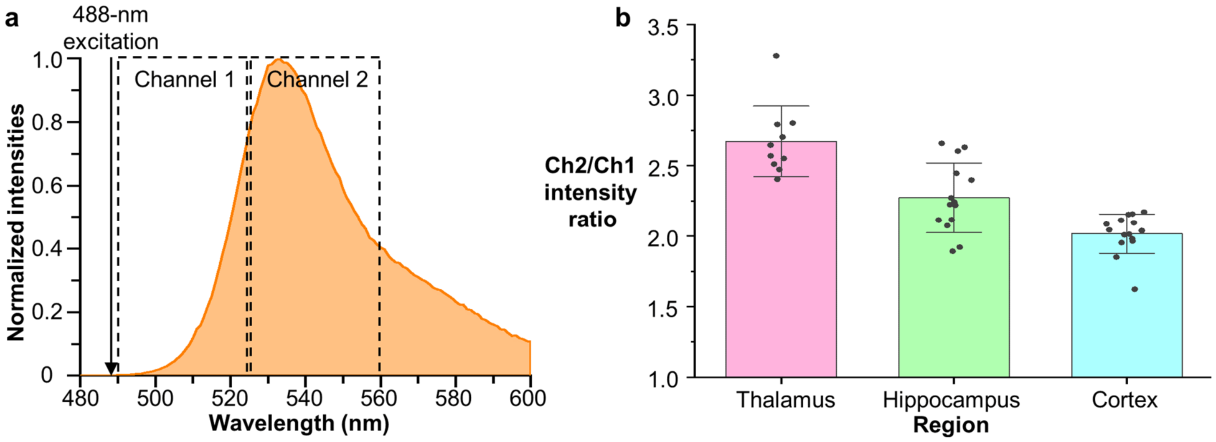

Supplementary Figure 2. **Variations in the emission spectra of fluorophores depend on the subregions where the spectra were acquired**. Images of mouse brain slices stained with an ATTO514-conjugated antibody against lamin B1 were acquired from three subregions (the hippocampus, the thalamus, and the cortex) of the brain at two detection channels. Then, the fluorescence intensity ratios between the two channels were calculated. As with **Supplementary Fig**. **1**, pixels with the top 1% brightness were used for the calculation. Given that large spectral variation was observed around the peak of the fluorophores (**Supplementary Fig**. **1a**, **b**), we measured the fluorescence intensity ratio between two channels to estimate the subregion-specific spectral characteristics of ATTO514 in this experiment. (**a**) The emission spectra of ATTO514 and two detection channels (channel 1: 490–525 nm and channel 2: 525–560 nm) used in this study. (**b**) The fluorescence intensity ratios between the two channels measured from three subregions: the thalamus: 2.67 ± 0.25 (mean ± standard deviation), the hippocampus: 2.27 ± 0.25 and the cortex: 2.02 ± 0.14. The number of intensity ratio analysed was: thalamus, 10 from one specimen; hippocampus, 14 from one specimen; cortex, 15 from one specimen. Note that the fluorescence intensity ratio varied depending on the subregion of the brain. It also showed a significant level of variation within the same subregion.

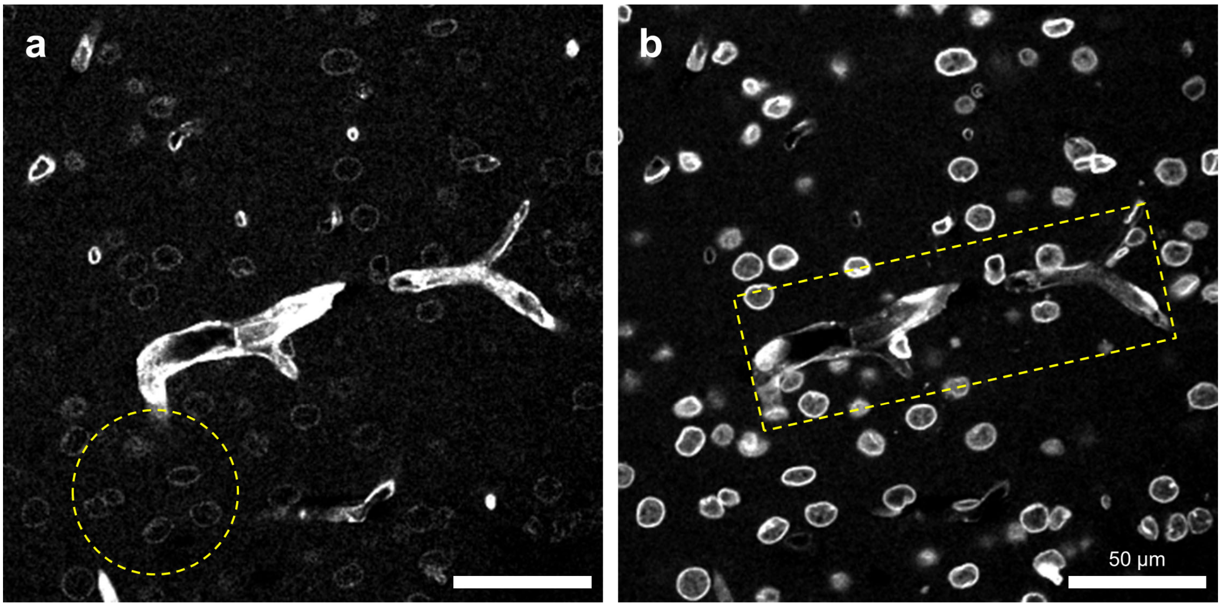

Supplementary Figure 3. **Unmixing via NMF**. A mouse brain slice was stained with two preformed antibody complexes against GluT1 (CF488A) and lamin B1 (ATTO514), then imaged at 32 detection channels with a 10-nm bandwidth from 400 nm to 720 nm. The 32-channel images were unmixed via NMF. (**a**) Channel 1 of the unmixed images. Only GluT1 signals should be visible, but the lamin B1 signal (yellow circled region) is visible. (**b**) Channel 2 of the unmixed images. Only the lamin B1 signal should be visible, but the GluT1 signal (yellow boxed region) is visible.

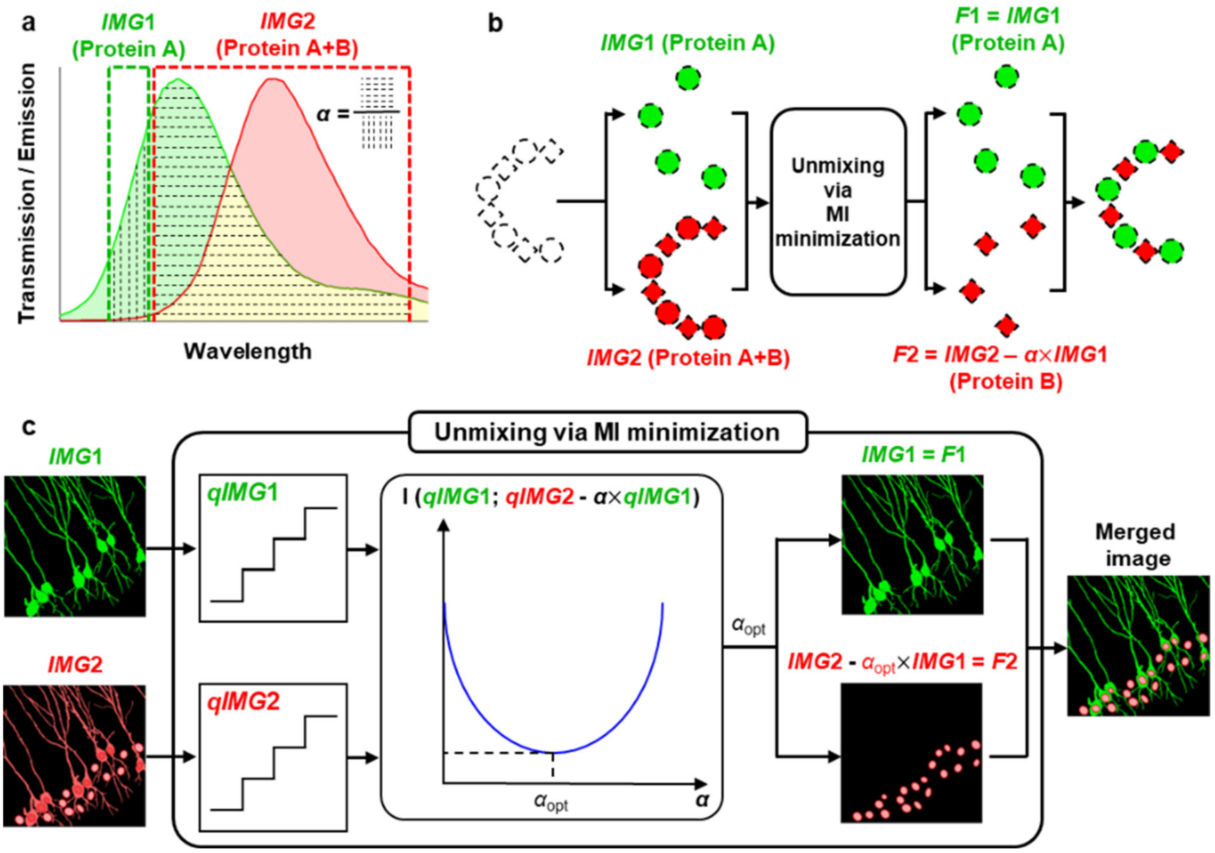

Supplementary Figure 4. **Experimental design to validate the use of MI minimization as a means of unmixing**. (**a**) Emission spectra of two spectrally overlapping fluorophores (green and red-colored regions) and detection channels (green and red dotted boxes) used in this validation study. Here, α is the ratio of the area with horizontal dotted lines to that with vertical dotted lines. IMG1, 2 images acquired at the first (green dotted box) and second (red dotted box) detection channels. (**b**) Example of PICASSO images before and after unmixing. Dotted circles and squares: two target proteins to be imaged in a specimen. Colored circles and squares: structures shown in images. $\mathrm{F1, F2}$: images after unmixing. (**c**) Unmixing via MI minimization. qIMG1,2: quantized IMG1,2. $\mathrm{I(qIMG1; qIMG2} - \alpha \times \mathrm{qIMG1)}$: MI between qIMG1 and $\mathrm{qIMG2} - \alpha \times \mathrm{qIMG}$. α_{opt}: α minimizing $\mathrm{I(qIMG1; qIMG2} - \alpha \times \mathrm{qIMG1)}$.

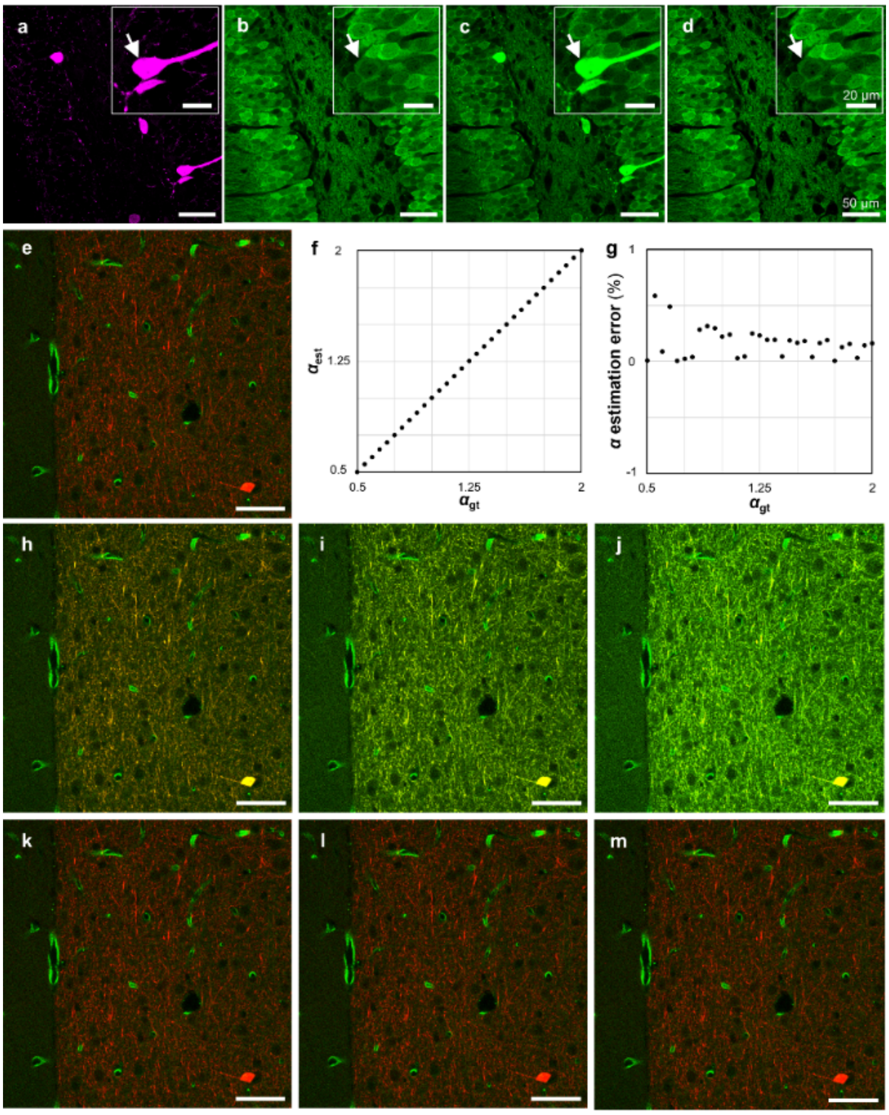

Supplementary Figure 5. **Validation of the accuracy of the unmixing via MI minimization**. (**a**–**d**) Validation of unmixing via MI minimization. (**a**, **b**) Immunofluorescence image of parvalbumin (**a**) and calbindin (**b**). (**c**) Synthetic mixed image (SynIMG) of **a** (F1) and **b** (F2) with an α value of 2 (SynIMG = $2 \times$ F1 + F2). Two mixed images, which are F1 and SynIMG, were unmixed via MI minimization. (**d**) The resulting unmixed image, showing only calbindin. Note that the image in **d** (after unmixing) is highly consistent with the image in **b** (ground-truth image). (**e**–**m**) Quantitative analysis of the accuracy of unmixing via MI minimization. (**e**) Two-color image of a mouse brain slice stained with antibodies against calbindin and GluT1. Red (F1), calbindin; green (F2), GluT1. (**f**) Ground-truth α_{gt} vs. estimated α (= α_{est}). The two channels shown in **e** were used to generate a synthetic mixed image with a known α (= α_{gt}).

The synthetic mixed images were then unmixed via MI minimization, and the resulting estimated α_{est} was compared with the ground-truth α_{gt}. (**g**) α estimation error. (**h**–**j**) Synthetic mixed images used for the simulation. Red, same as the calbindin channel (red) of the image shown in **e**; green, mixed image obtained by the linear addition of the calbindin channel (red) of the image shown in **e** multiplied by α_{gt} and the GluT1 channel (green) of the image shown in **e**. α_{gt} was (**h**) 0.5, (**i**) 1.25, (**j**) 2. (**k**–**m**) Unmixed two-channel images obtained by unmixing via MI minimization. The image shown in **k**, **l**, and **m** was obtained by unmixing the image shown in **h**, **i**, and **j**, respectively. Scale bars: 50 μm. Note that unmixed images shown in **k**, **l**, and **m** are highly consistent with the ground-truth image shown in **e**.

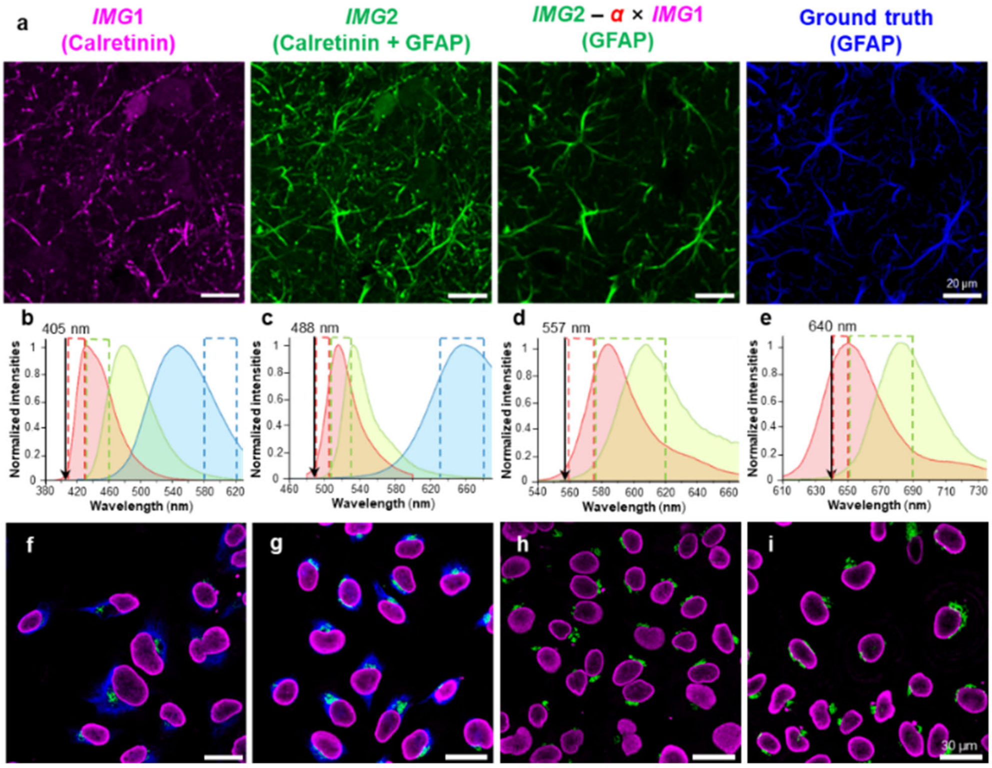

Supplementary Figure 6. **Experimental validation and demonstrations of unmixing via MI minimization**. (**a**) Experimental validation. A mouse brain slice was stained with two preformed antibody complexes against calretinin (CF568) and GFAP (ATTORho101) along with one regular antibody against GFAP (CF633). The regular antibody staining was used as the ground-truth image of GFAP. From the left to the right, IMG1, IMG2, unmixed image (IMG2 – α × IMG1), and the ground-truth image are shown respectively. Only the calretinin signal is visible in IMG1. Calretinin and GFAP signals are visible in IMG*2*. After unmixing, the calretinin signal was subtracted from IMG2. The resulting unmixed GFAP image was highly consistent with the ground-truth image. (**b**–**i**) Demonstrations of two- or three-color multiplexed imaging with a single excitation laser. (**b**–**e**) Normalized emission spectra of fluorophores (colored regions) and detection channels (dotted boxes) for each excitation laser (black arrow). (**b**) CF488A (red), ATTO514 (green), and ATTO490LS (blue) excited by a 488-nm laser. (**c**) CF405S (red), ATTO390 (green), and CF405L (blue) excited by a 405-nm laser. (**d**) CF568 (red) and ATTORho101 (green) excited by a 557-nm laser. (**e**) CF633 (red) and CF660R (green) excited by a 640-nm laser. (**f**–**i**) Two- or three-color multiplexed images of HeLa cells after unmixing via MI minimization. Magenta, lamin A/C; green, GM130; blue, vimentin. The fluorophores and detection channels shown in **b**–**e** were used.

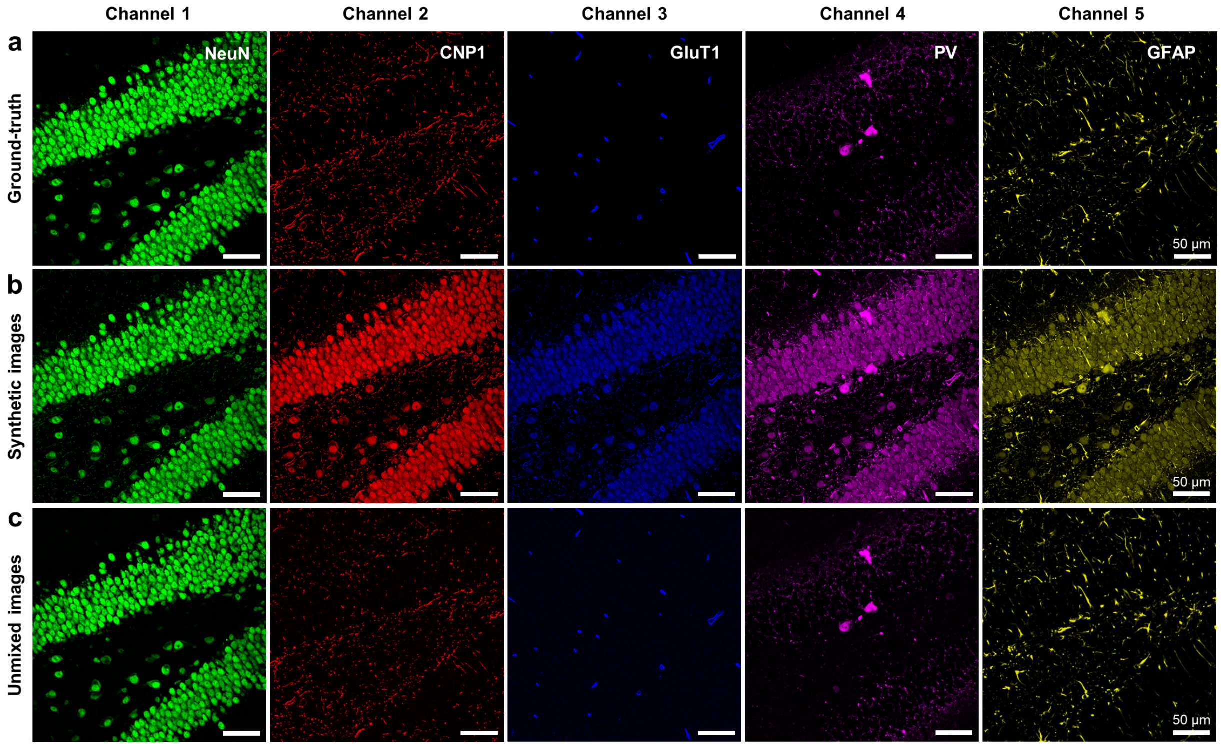

Supplementary Figure 7. **Single-channel images of Fig**. **2h**, **i**. (**a**) Five protein images of a mouse brain slice used for synthesizing mixed images. Green: NeuN, red: CNP1, blue: GluT1, magenta: PV, and yellow: GFAP. (**b**) Synthetic images. (**c**) Unmixed images via PICASSO.

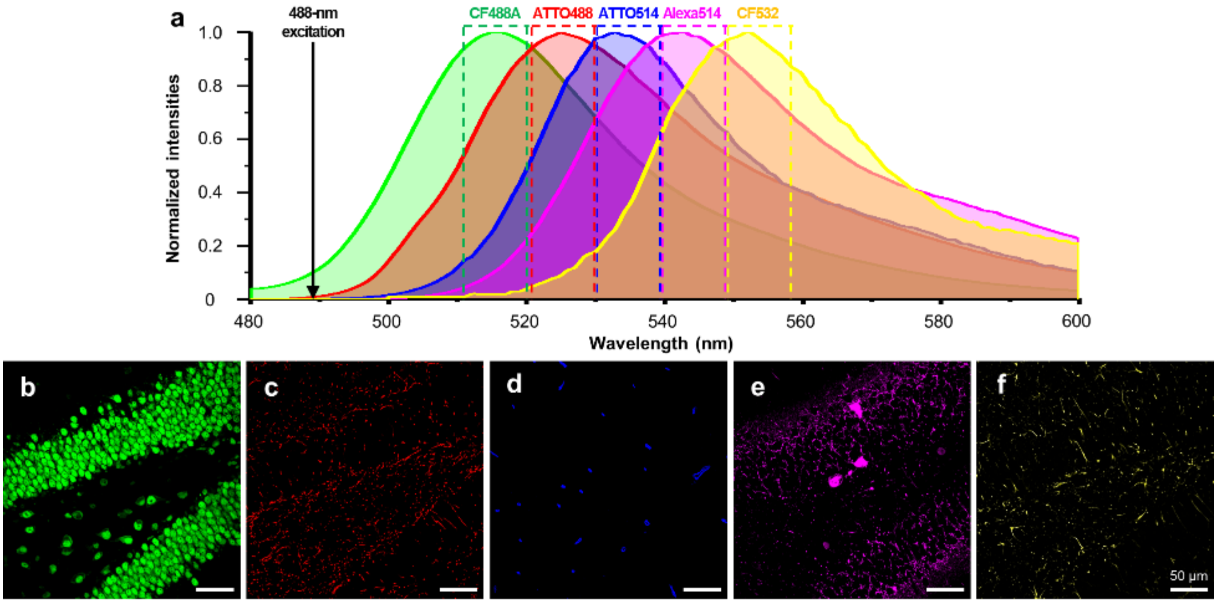

Supplementary Figure 8. **Unmixing of five highly overlapping fluorophores**. The ground-truth images shown in **Supplementary Fig**. **7a** were used to generate synthetic mixed images and then unmixed via PICASSO. (**a**) Emission spectra of the five fluorophores used in the simulation. Emission peaks of the fluorophores were separated by less than 10 nm (the emission peak of CF488A: 515.6 nm, ATTO488: 525 nm, ATTO514: 533 nm, Alexa Fluor 514: 543 nm, and CF532: 552 nm). The detection channels (dotted boxes) were defined from -5 to +5 nm of the emission peaks of the fluorophores. (**b**–**f**) Unmixed images. (**b**) NeuN (CF488A). (**c**) CNP1 (ATTO488). (**d**) GluT1 (ATTO514). (**e**) PV (Alexa Fluor 514). (**f**) GFAP (CF532). The structural similarity (SSIM) of unmixing was 0.9887.

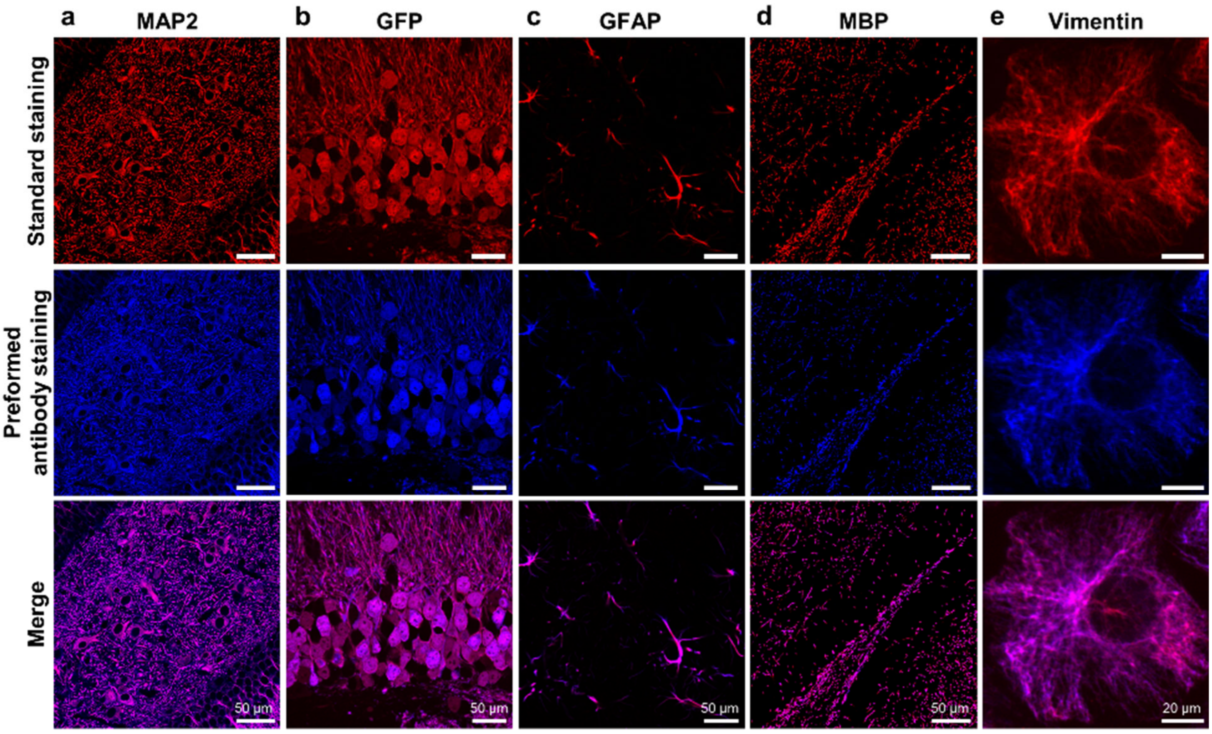

Supplementary Figure 9. **Validation of the staining with preformed antibody complexes**. Confocal microscopy images of mouse brain slices or cultured cells, each of which was stained with a conventional antibody and a preformed antibody complex against the same target proteins. The target proteins were (**a**) MAP2, (**b**) GFP, (**c**) GFAP, (**d**) MBP, and (**e**) vimentin. For **a**, **c**, **d**, wild-type mouse brain slices were used. For **b**, a Thy1-YFP mouse brain slice was used. For **e**, cultured BS-C-1 cells were used. For all tested targets, the conventional antibody staining and preformed antibody staining showed identical staining patterns, as shown in the 3rd row.

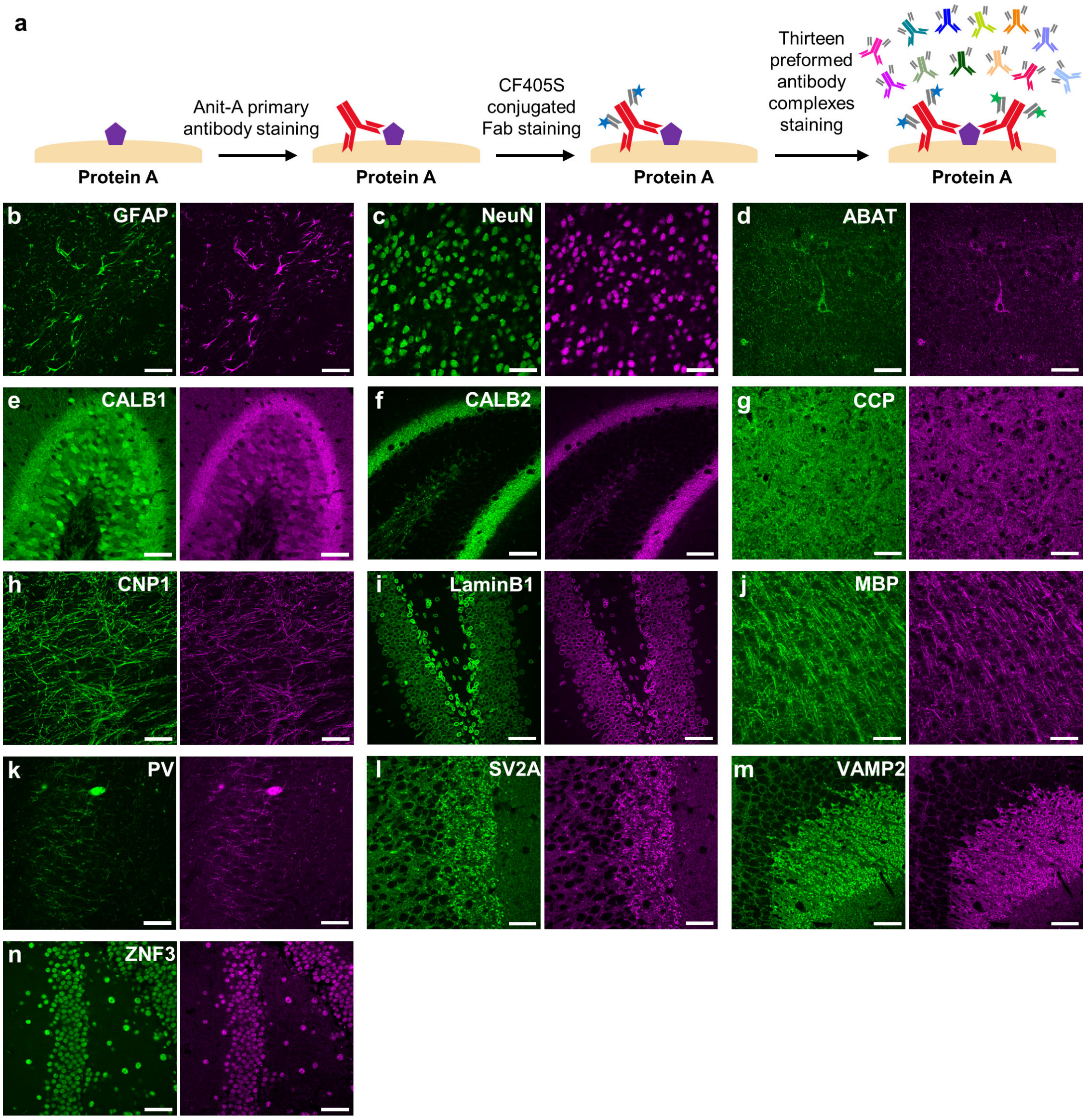

Supplementary Figure 10. **Validation of the absence of crosstalk among 13 preformed antibody complexes**. (**a**) Schematics of the crosstalk validation experiments. For generating a ground-truth channel, 13 mouse brain slices were stained against GFAP, NeuN, ABAT, CALB1, CALB2, CCP, CNP1, laminB1, MBP, PV, SV2A, VAMP2 and ZNF3 primary antibodies, respectively, and labeled with anti-rabbit CF405S conjugated Fab secondary antibody. Then, each brain slice was simultaneously stained with 13 preformed rabbit antibody complexes. (**b**–**n**) Green; ground-truth channel (CF405S fluorescence), magenta; signals of preformed antibody complexes (CF488A fluorescence). (**b**) GFAP. (**c**) NeuN. (**d**) ABAT. (**e**) CALB1. (**f**) CALB2. (**g**) CCP. (**h**) CNP1. (**i**) LaminB1. (**j**) MBP. (**k**) PV. (**l**) SV2A. (**m**) VAMP2. (**n**) ZNF3. No crosstalk among those preformed antibody complexes was visible. All scale bars: 50 μm.

Mouse brain

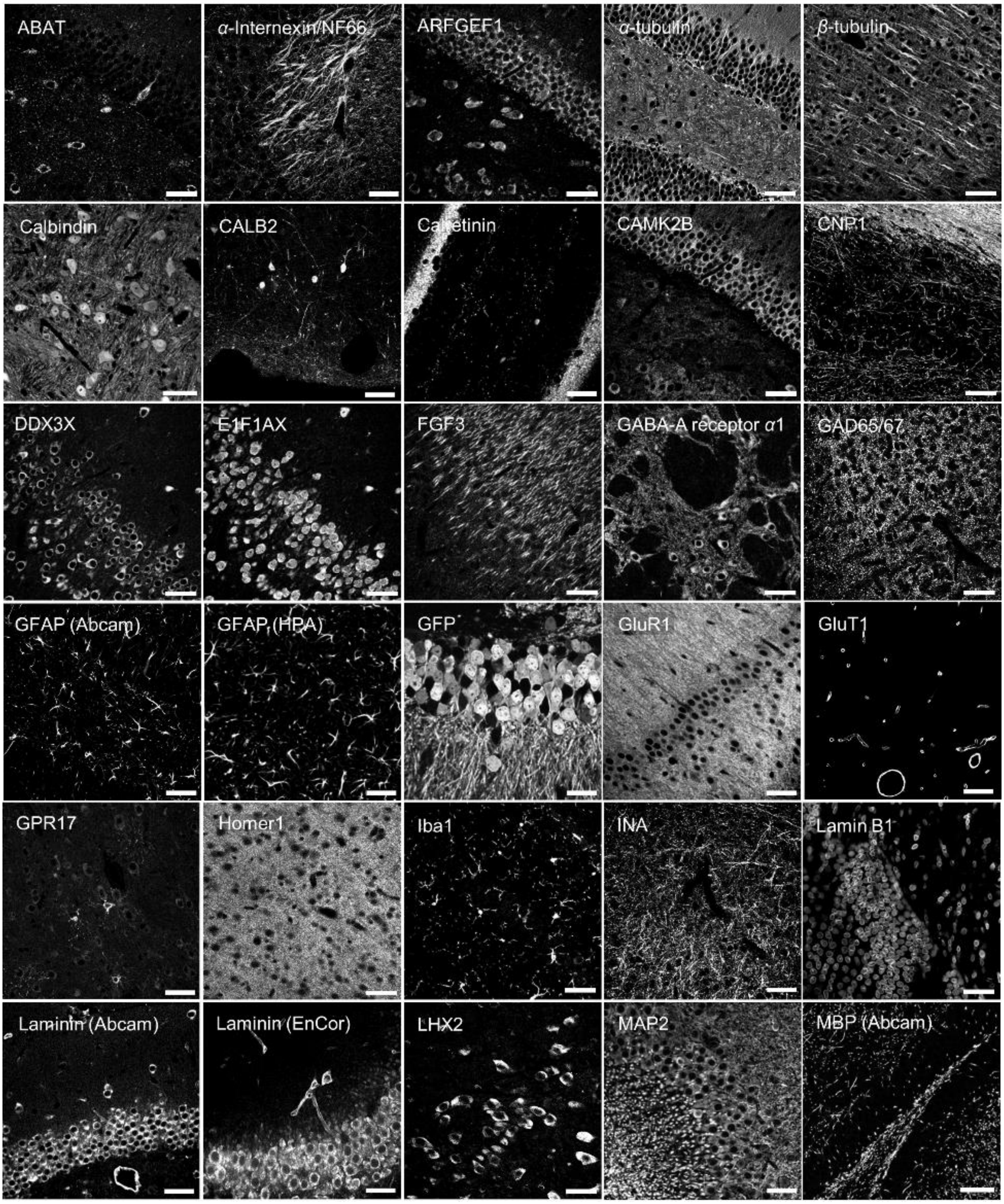

ABAT
α-Internexin/NF66
ARFGEF1
α-tubulin
β-tubulin
Calbindin
CALB2
Calretinin
CAMK2B
CNP1
DDX3X
E1F1AX
FGF3
GABA-A receptor α1
GAD65/67
GFAP (Abcam)
GFAP (HPA)
GFP
GluR1
GluT1
GPR17
Homer1
Iba1
INA
Lamin B1
Laminin (Abcam)
Laminin (EnCor)
LHX2
MAP2
MBP (Abcam)

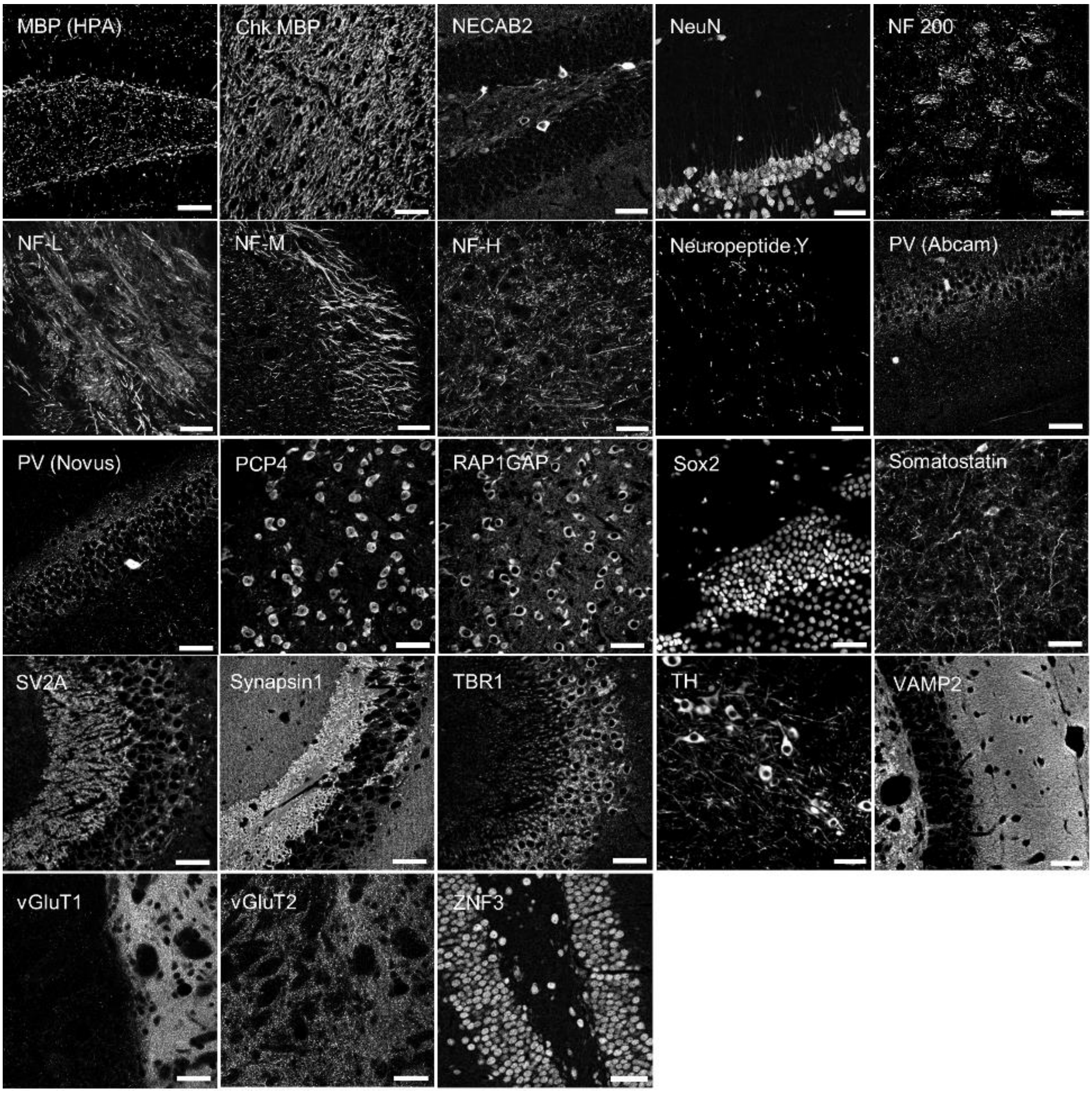
MBP (HPA)
Chk MBP
NECAB2
NeuN
NF 200
NF-L
NF-M
NF-H
Neuropeptide Y
PV (Abcam)
PV (Novus)
PCP4
RAP1GAP
Sox2
Somatostatin
SV2A
Synapsin1
TBR1
TH
VAMP2
vGluT1
vGluT2
ZNF3

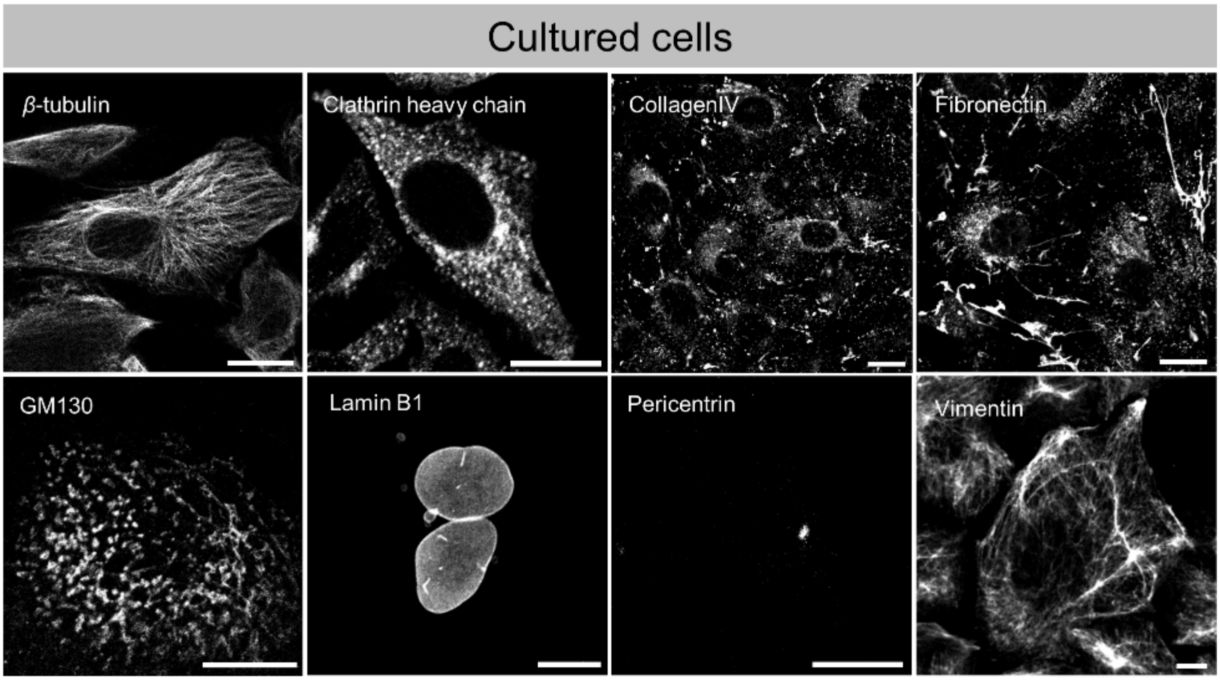

Supplementary Figure 11. **Confocal microscopy images of various proteins labeled with preformed antibody complexes**. Fifty-nine different antibodies were used, targeting diverse types of proteins, such as cell type-specific proteins, receptor proteins, transcription factor proteins, neurofilament-related proteins, and synaptic vesicle proteins. Detailed information about the antibodies is given in **Supplementary Data 1**. Scale bars: brain, 50 μm; cell, 20 μm.

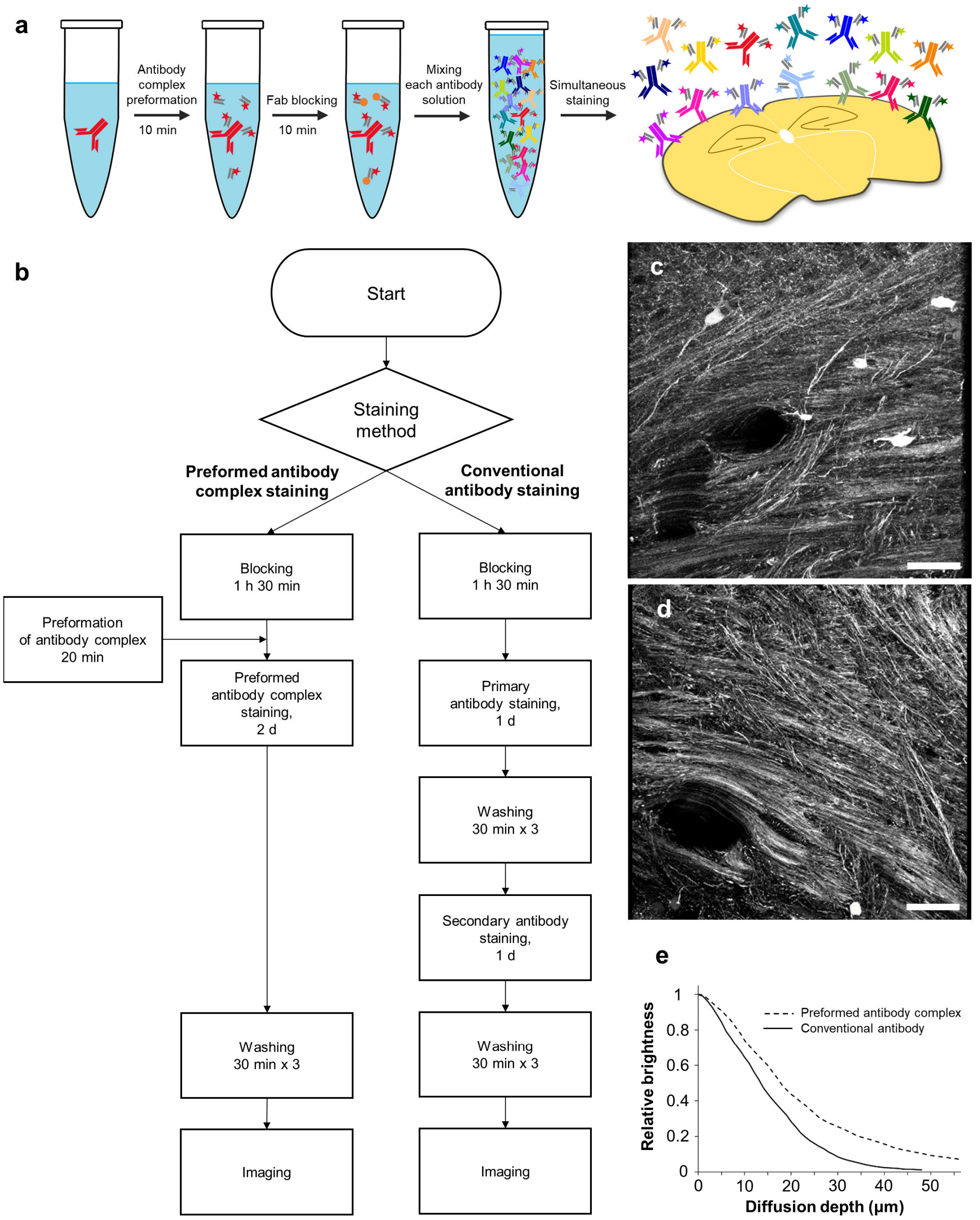

Supplementary Figure 12. Comparison of the diffusion depths between staining with a preformed antibody complex and a conventional antibody. (**a**) Schematics of antibody preformation process. (**b**) Experiment workflow. (**c**, **d**) Maximum intensity projections of z-stack images of the thalamus from mouse brain slices stained with either a preformed antibody complex or a regular antibody against calbindin. (**c**) Result of a preformed antibody complex. (**d**) Result of a conventional antibody. (**e**) Diffusion depth profiles of the specimens shown in **c** and **d**. Scale bars: 50 μm.

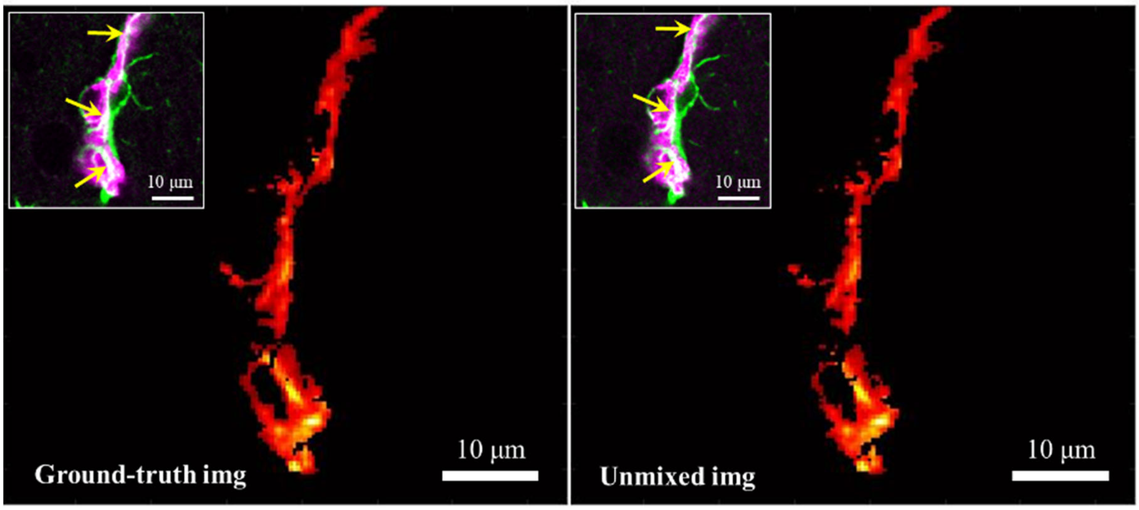

Supplementary Figure 13. **Comparison of the colocalized pixels between the ground-truth image and the unmixed image**. To measure the unmixing accuracy of PICASSO for images of spatially overlapping proteins, pixels where both GluT1 and GFAP were highly expressed were selected from the inset of the ground-truth (**Fig**. **3e**) and unmixed images (**Fig**. **3f**). SSIM between the two images was 0.9846, indicating that PICASSO successfully unmixed two mixed images of two spatially overlapping proteins.

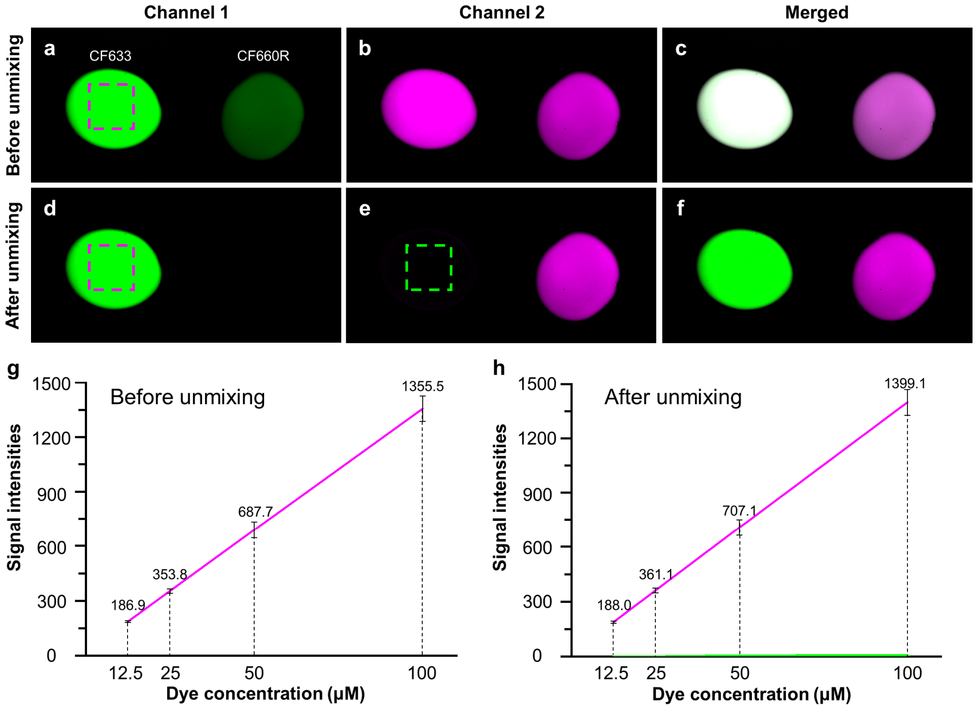

Supplementary Figure 14. **Quantitative analysis of PICASSO unmixing**. (**a**–**f**) CF633 and CF660R fluorophore solutions were placed on coverglass, imaged at two different spectral ranges and unmixed via PICASSO. The concentration of CF633 was manually adjusted to figure out whether PICASSO unmixing can retain signal intensities of fluorophores based on the concentration. Left droplet: CF633, and right droplet: CF660R. (**a**) Mixed image acquired at the first channel. (**b**) Mixed image acquired at the second channel. (**c**) Merged image of **a** and **b**. (**d**) Unmixed image of CF633. (**e**) Unmixed image of CF660R. (**f**) Merged image of **d** and **e**. (**g**, **h**) Linear regression plots of the signal intensities according to the concentrations of CF633. (**g**) The signal intensities of CF633 before unmixing. The intensities were measured in mixed images acquired at the first channel, as shown in **a** (magenta box). R^2 = 0.99. (**h**) The signal intensities of CF633 after unmixing. The intensities were measured in unmixed images, as shown in **d** and **e** (magenta and green boxes). Magenta line: intensities measured at the first channel after unmixing. Green line: intensities measured at the second channel after unmixing. R^2 = 0.99.

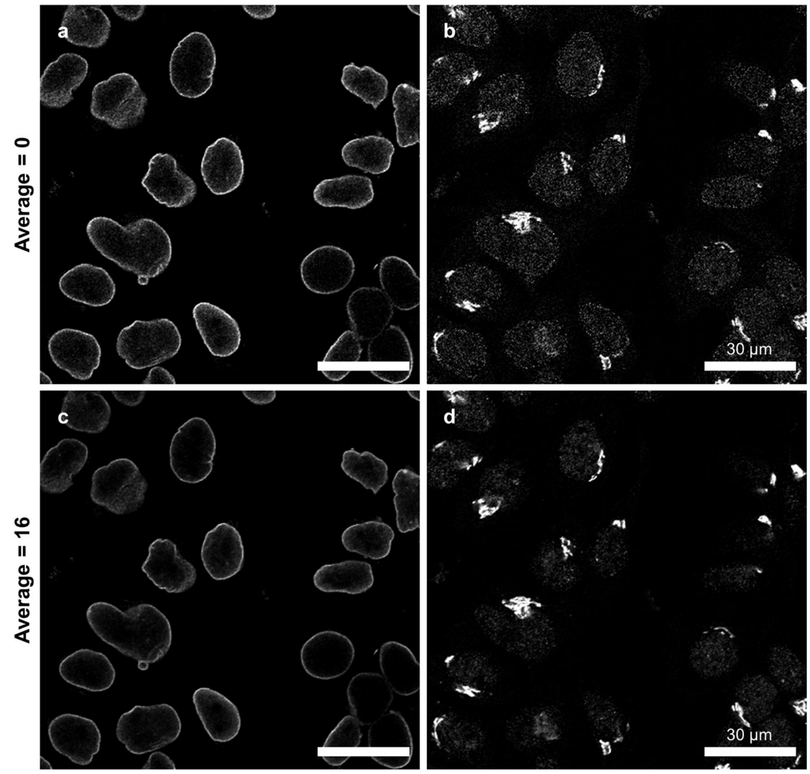

Supplementary Figure 15. **Reduction in the Poisson noise via image averaging**. Study on the Poisson noise level in the unmixed images depending on the number of averagings during the image acquisition step. Lamin A/C and GM130 of cultured cells were stained with two spectrally overlapping fluorophores and then imaged at two detection channels twice. At the first image acquisition, images were acquired without any averaging. At the second image acquisition, images were acquired 16 times and then averaged. The images were then unmixed via MI minimization. (**a**, **b**) Result of unmixing via PICASSO without image averaging. Considerably high noise values were observed in the unmixed images. (**c**, **d**) Result of unmixing via PICASSO with image averaging. A significant reduction in the noise was observed in the unmixed images.

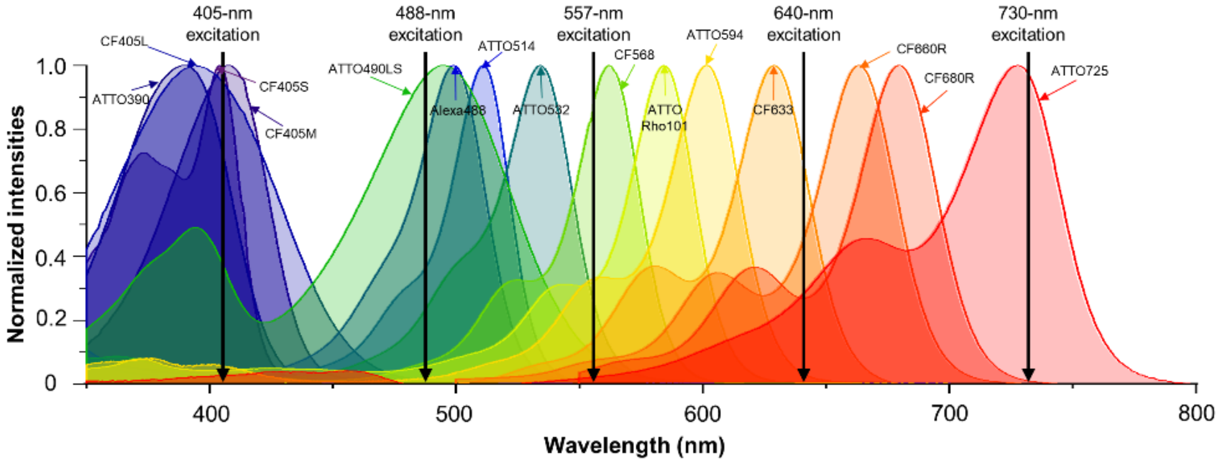

Supplementary Figure 16. **Excitation spectra of the 15 fluorophores used in Fig. 4b**. Black arrows indicate the wavelengths of the five standard excitation lasers.

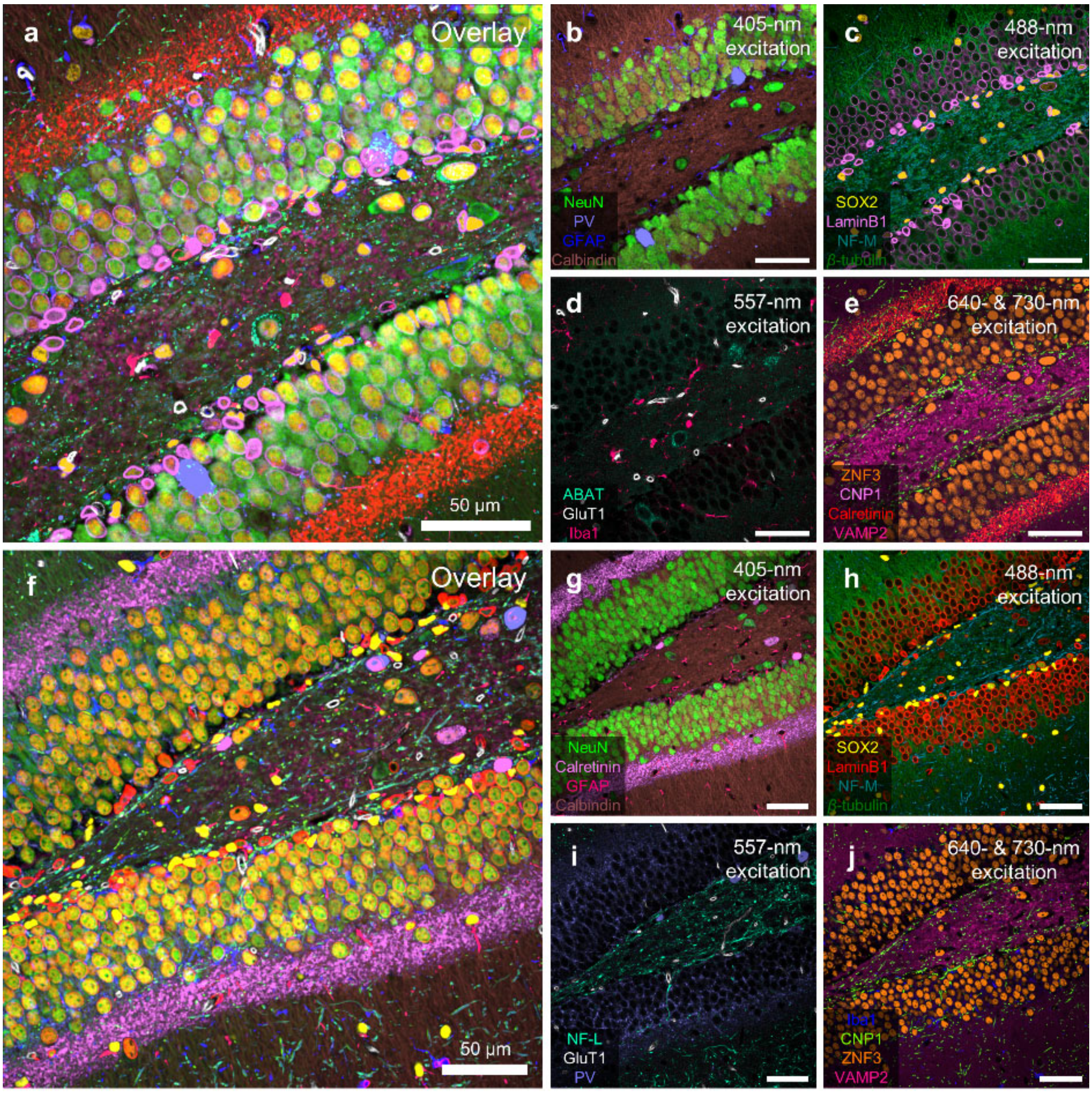

Supplementary Figure 17. **Additional demonstrations of 15-color multiplexed imaging**. Fifteen-color multiplexed images acquired from the dentate gyrus of the mouse hippocampus of different mouse brain slices. To confirm that the 15-color multiplexed imaging works reproducibly, different antibody combinations were used in this experiment from those used in **Fig. 4b**. Detailed information about the antibodies is shown in **Supplementary Data 2**. (**a**) Overlaid image. (**b–e**) Unmixed images acquired from each excitation laser. (**f–j**) Another 15-color multiplexed imaging of a different antibody combination. (**f**) Overlaid image. (**g–j**) Unmixed images acquired from each excitation laser.

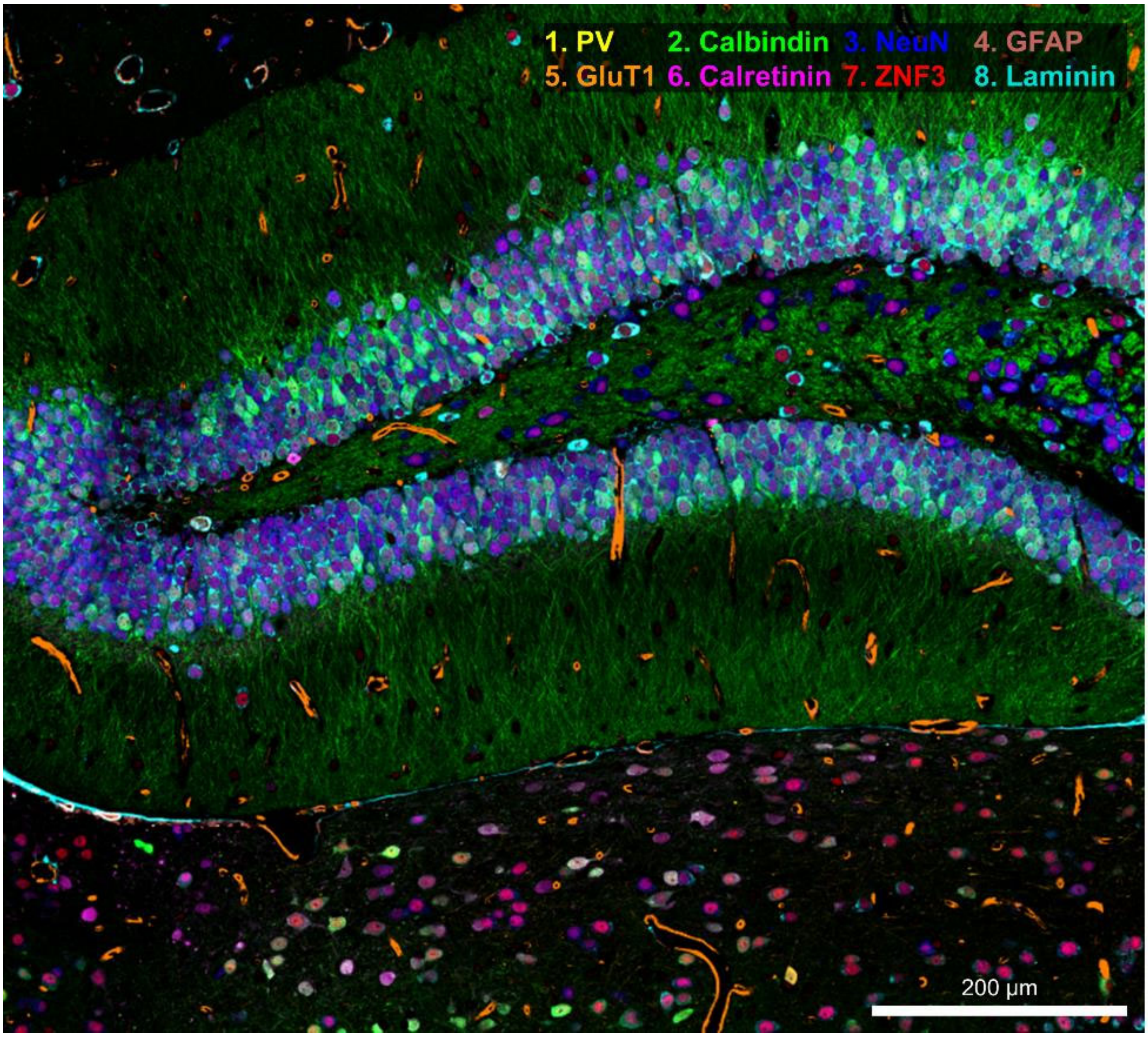

Supplementary Figure 18. **An enlarged image of Figure 5a**.

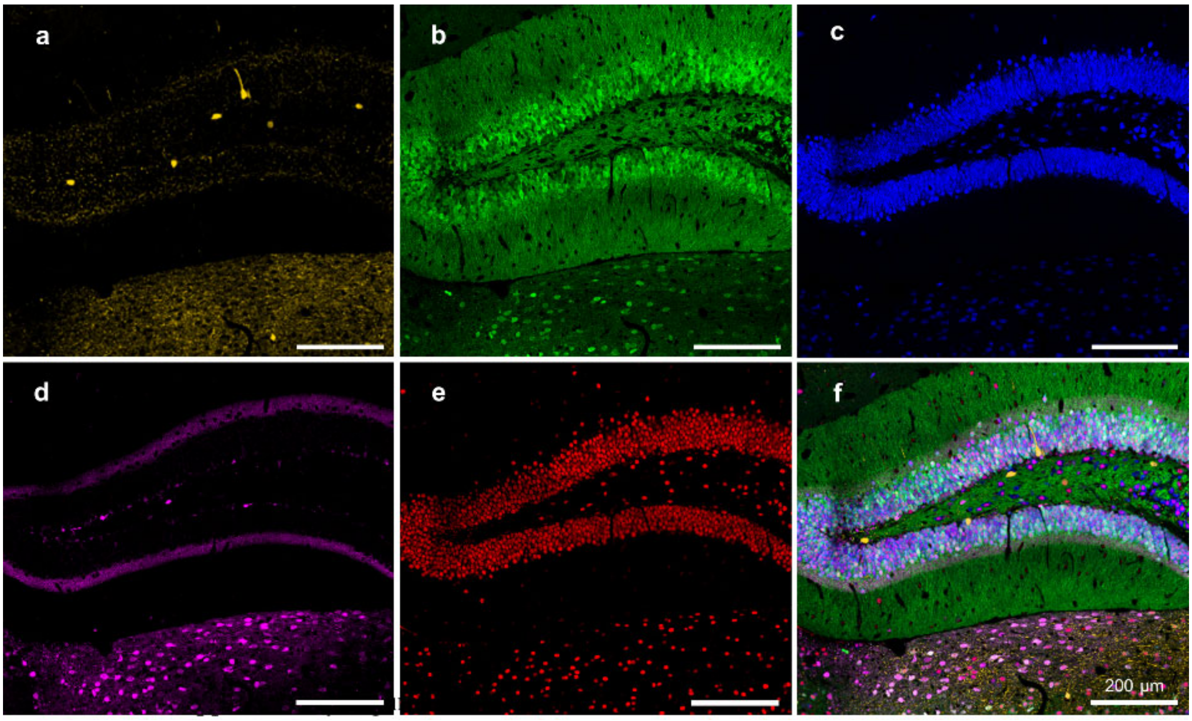

Supplementary Figure 19. **Five single-channel images of Figure 5a**. (**a**–**e**) Single-channel counterparts of the 8-color multiplexed image shown in **Fig**. **5a** clearly showing the spatial distribution of each protein in the mouse dentate gyrus and thalamus. (**a**) Yellow: parvalbumin (PV). (**b**) Green: calbindin. (**c**) Blue: NeuN. (**d**) Magenta: calretinin. (**e**) Red: zinc-finger protein 3 (ZNF3). (**f**) Merged image.

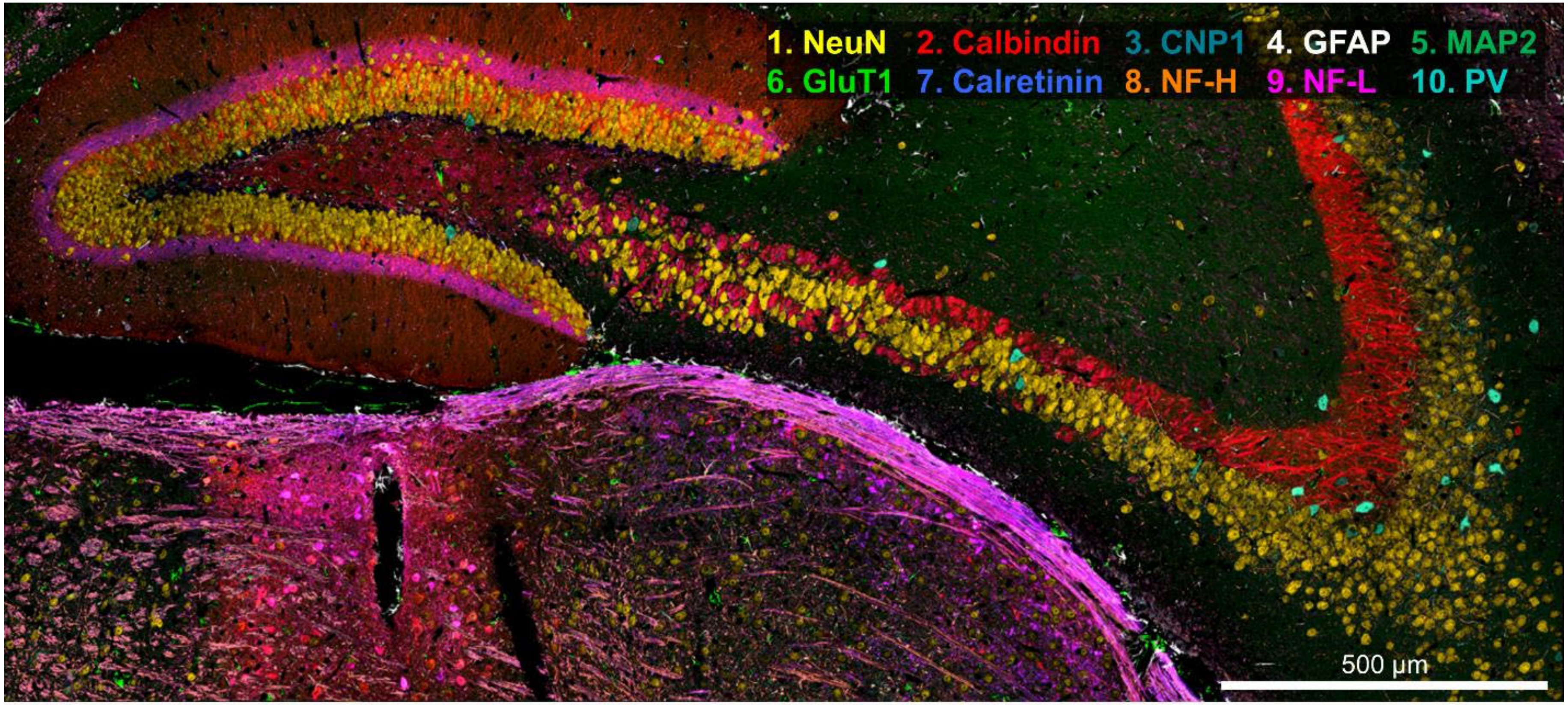

Supplementary Figure 20. **An enlarged image of Figure 5n**.

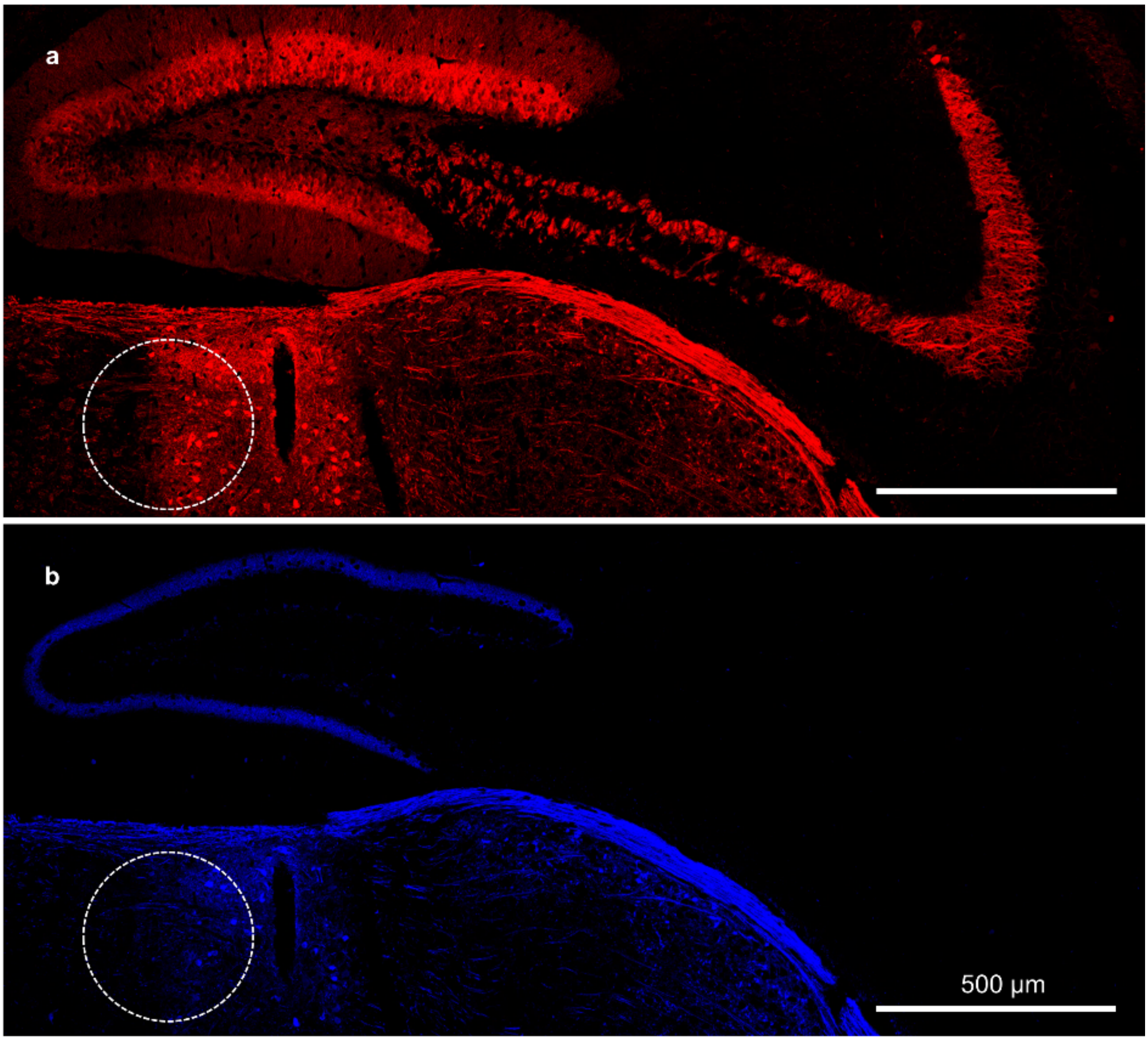

Supplementary Figure 21. **Two single-channel images of Figure 5n, showing calbindin and calretinin**. Dotted circles indicate the boundary between the lateral posterior nucleus of the thalamus and the anterior pretectal nucleus of the midbrain. Calbindin and calretinin were highly expressed in the lateral posterior nucleus. (**a**) Red: calbindin. (**b**) Blue: calretinin.

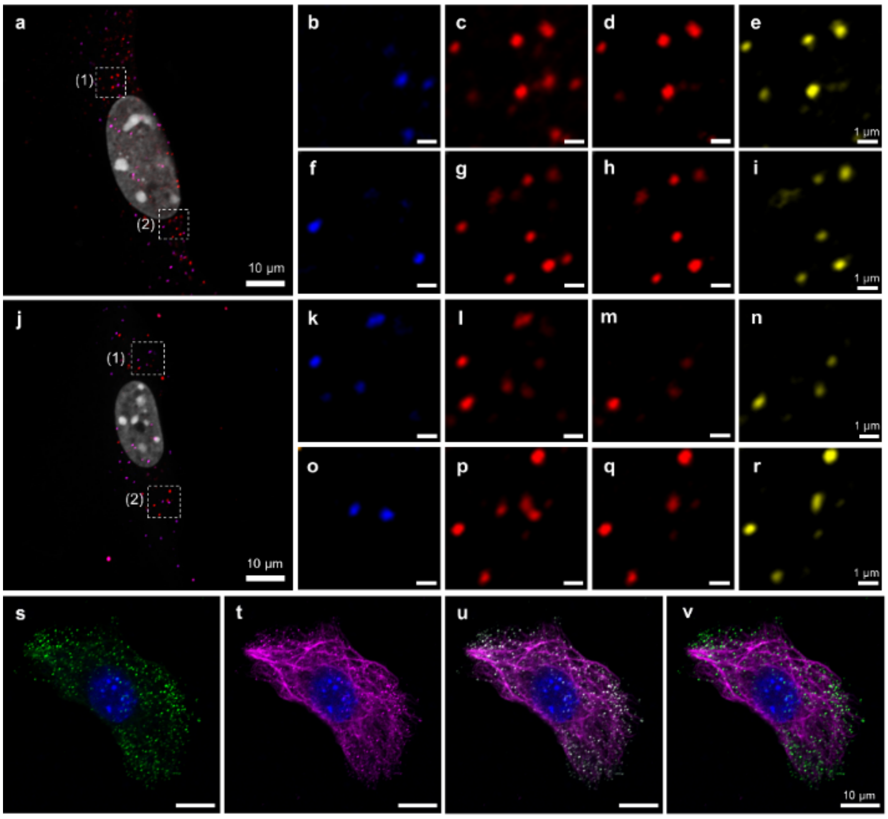

Supplementary Figure 22. **Application of PICASSO to multiplexed mRNA imaging and simultaneous imaging of mRNA and protein**. (**a**–**r**) Multiplexed imaging of two mRNAs with a single excitation laser via PICASSO and its validation. *Eif1a* and *Polr2a* mRNAs were labeled with two spectrally overlapping fluorophores. In addition, *Polr2a* mRNA was labeled with a spectrally distinct fluorophore for acquiring the ground-truth image. (**a**) A merged image containing three channels, including two mixed channels (blue and red) and DAPI (white). (**b**–**e**) Magnified views of the white dotted box (1) in **a**. (**b**) Image acquired at the first detection channel, showing only *EiF1a* mRNA. (**c**) Image acquired at the second detection channel, showing both *Eif1a* and *Polr2a* mRNA. (**d**) Second channel after unmixing via GS orthogonalization, showing only *Polr2a* mRNA. (**e**) Ground-truth image of *Polr2a* mRNA. Note that the *Polr2a* mRNA puncta shown in **d** and **e** coincide. (**f**–**i**) As in **b**–**e** but for the white dotted box (2). (**j**–**r**) As in **a**–**i** but showing a different cell. (**s**–**v**) Simultaneous imaging of mRNA and protein with a single excitation laser via PICASSO. The anti-vimentin antibody and the *Gapdh* mRNA bore spectrally overlapping fluorophores. (**s**) Image acquired at the first detection channel, showing only *Gapdh* mRNA. (**t**) Image acquired at the second detection channel, showing both *Gapdh* mRNA and vimentin. (**u**) A merged image combining **s** and **t**. (**v**) Merged image after unmixing via PICASSO. Magenta, vimentin; green, *Gapdh* mRNA; blue, DAPI.

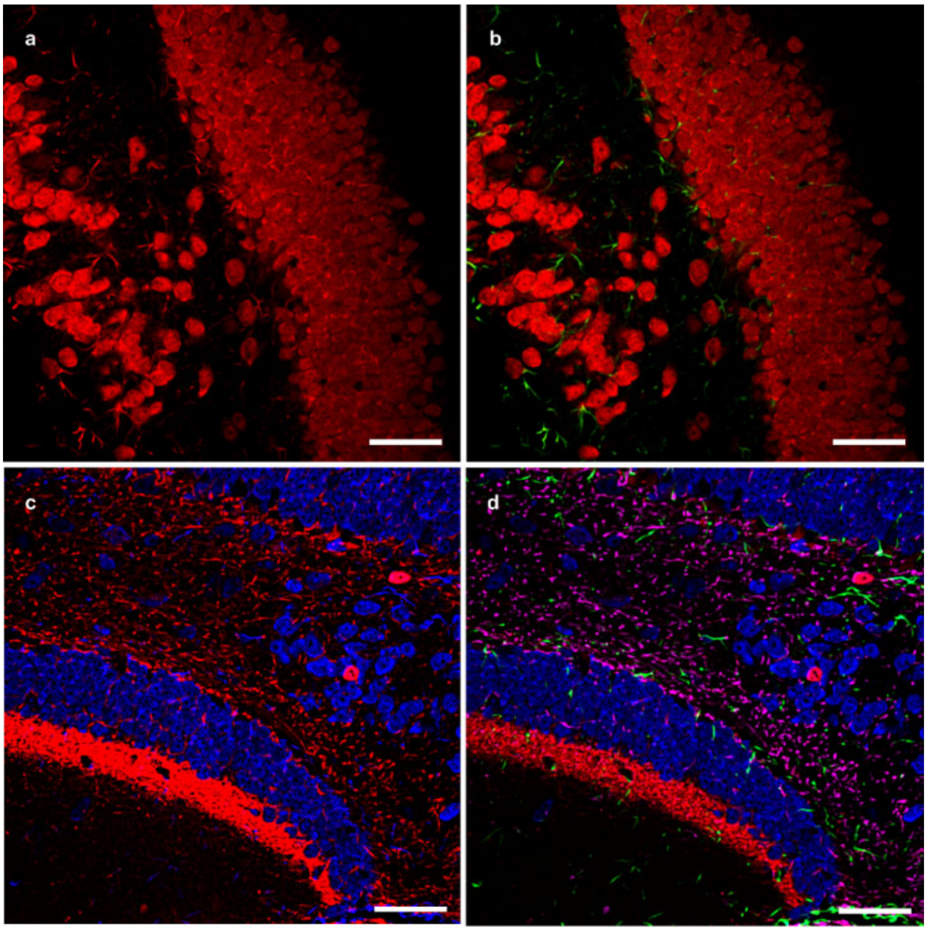

Supplementary Figure 23. **Application of PICASSO to expansion microscopy (ExM) and SHIELD**. (**a**, **b**) Two-color multiplexed imaging of an ExM-processed mouse brain slice with one excitation laser via PICASSO. (**a**) Image acquired at the second detection channel, showing both NeuN and GFAP before unmixing. (**b**) Merged two-channel image after unmixing via PICASSO. Red, NeuN; green, GFAP. (**c**, **d**) Four-color multiplexed imaging of a SHIELD-processed mouse brain slice imaged with two excitation lasers via PICASSO. (**c**) Merged image of two channels, each of which was acquired at the second detection channel under the illumination of different excitation lasers before unmixing. Blue, NeuN and GFAP, acquired with a 488-nm excitation laser; Red, calretinin and NF-H, acquired with a 557-nm laser. The images acquired at the first detection channel are not shown here. (**d**) After unmixing. Blue, NeuN; green, GFAP; red, calretinin; magenta, NF-H. Scale bars: 50 μm.

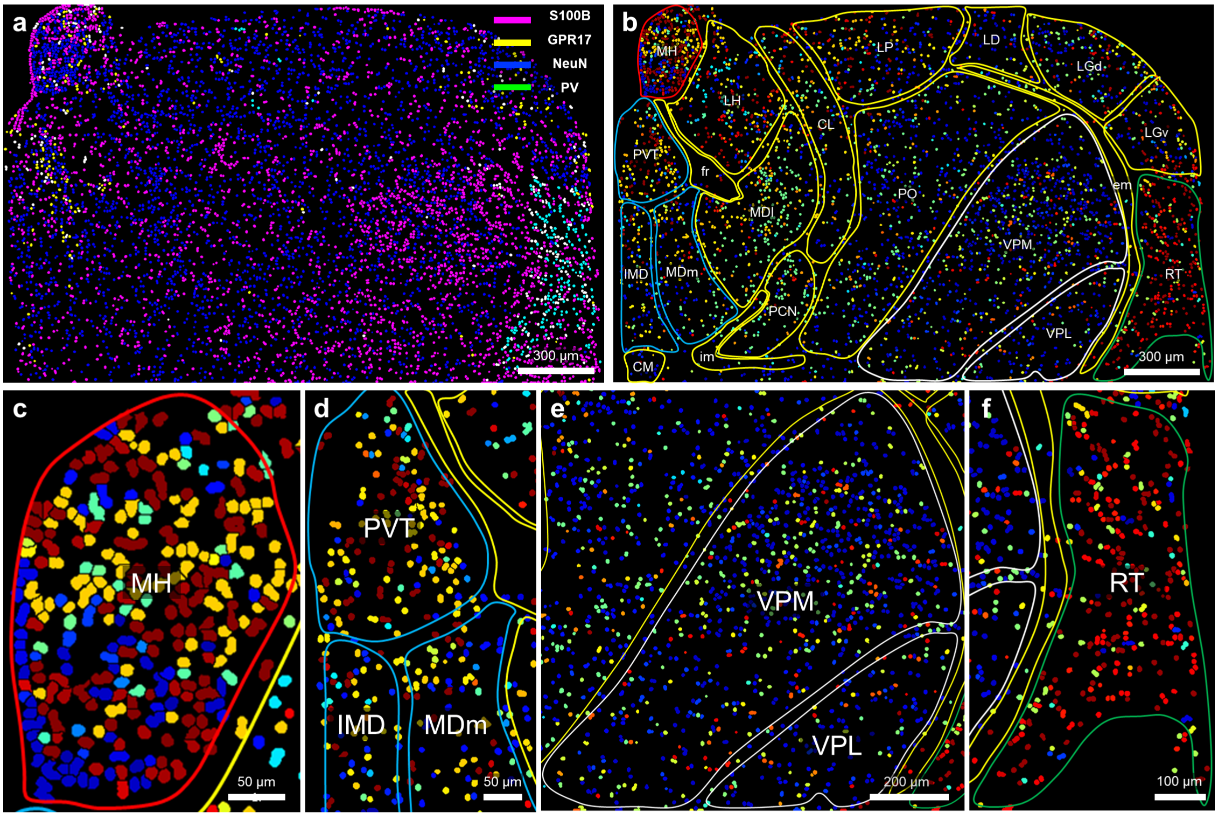

g

Region	Whole region			MH			PVT, IMD, Mdm			VPM & VPL			RT		
Rank	Top 1	Top 2	Top 3	Top 1	Top 2	Top 3	Top 1	Top 2	Top 3	Top 1	Top 2	Top 3	Top 1	Top 2	Top 3
Cell type	#1	#2	#3	#4	#5	#6	#4	#5	#7	#1	#8	#9	#10	#11	#12
# of cells	528	422	321	68	67	33	58	53	37	421	99	77	86	61	35
Ratio	13.7%	11.0%	8.3%	24.7%	24.4%	12.0 %	21.3%	19.5%	13.6%	44.5%	10.5%	8.1%	33.6%	23.8%	13.7%

h

Cell type	Cell color	Necab2	Sox2	Rap1gap	Calretinin	Calbinidn	ZNF3	ABAT
#1		+	+	+	-	-	+	+
#2		+	+	+	-	+	+	+
#3		+	-	+	-	+	+	+
#4		+	-	+	+	+	+	+
#5		+	+	+	+	+	+	+
#6		+	+	+	+	-	+	+
#7		+	+	-	+	+	+	+
#8		+	-	+	-	-	+	+
#9		-	+	+	-	-	+	+
#10		-	-	-	-	-	+	+
#11		+	-	-	-	-	+	+
#12		-	-	-	-	-	-	+

i

CL: Central lateral nucleus of the thalamus
CM: Central medial nucleus of the thalamus
Em: External medullary lamina of the thalamus
Fr: Fasciculus retroflexus
Im: Internal medullary lamina of the thalamus
IMD: Intermediodorsal nucleus of the thalamus
LCd: Dorsal part of the lateral geniculate complex
LCv: Ventrall part of the lateral geniculate complex
LD: : Lateral dorsal nucleus of the thalamus
LH: Lateral habenula
LP: Lateral posterior nucleus of the thalamus
MDl: Mediodorsal nucleus of the thalamus, lateral part
MDm: Mediodorsal nucleus of the thalamus, medial part
MH: Medial habenula
PCN: Paracentral nucleus
PO: Posterior complex of the thalamus
PVT: Paraventricular nucleus of the thalamus
RT: Reticular nucleus of the thalamus
VPL: Ventral posterolateral nucleus of the thalamus
VPM: Ventral posteromedial nucleus of the thalamus

Supplementary Figure 24. Analysis of the expression patterns of 11 proteins in the thalamus of a mouse brain slice through PICASSO. (**a**) Distribution of four cell marker proteins; S100B (magenta,

astrocyte marker), GPR17 (yellow, oligodendrocyte marker), NeuN (blue, neuron marker), and PV (green, GABAergic interneuron marker). Since PV-positive neurons also expressed NeuN, those cells were shown in cyan. (**b**–**f**) Color-coded neurons depending on their phenotypes. Cells were divided into 127 phenotypes (2^7-1) based on whether each of the seven proteins (necab2, sox2, rap1gap, calretinin, calbindin, ZNF3, and ABAT) was expressed or not. Each subtype was represented by a distinct color. Subregions of the thalamus (red, cyan, yellow, and green lines) were visually inspected and designated by referring to the 74th slice of the Atlas Viewer provided by the Allen Institute. (**c**) Enlarged image of MH in **b**. (**d**) Enlarged image of PVT, IMD, and MDm in **b**. (**e**) Enlarged image of VPM and VPL in **b**. (**f**) Enlarged image of RT in **b**. (**g**) Neuron phenotypes and distribution analysis results for four subregions shown in **c**–**f**. (**h**) Phenotypes and their colors shown in **b**–**g**. (**i**) Abbreviation of the subregions shown in **b**–**f**.

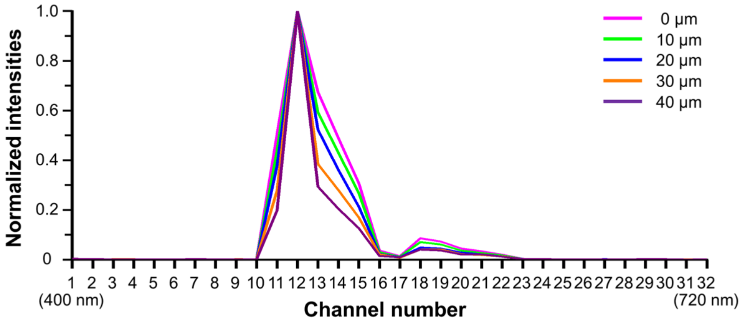

Supplementary Figure 25. **Spectral variation depending on the imaging depth**. A mouse brain slice was stained with a CF488A-conjugated antibody against NeuN and imaged at the surface of the slice at 10 μm, 20 μm, 30 μm, and 40 μm from the surface of the slice using confocal microscopy equipped with a spectral detector. Then, the normalized emission spectra were measured from the images acquired at different depths from the surface of the slice.

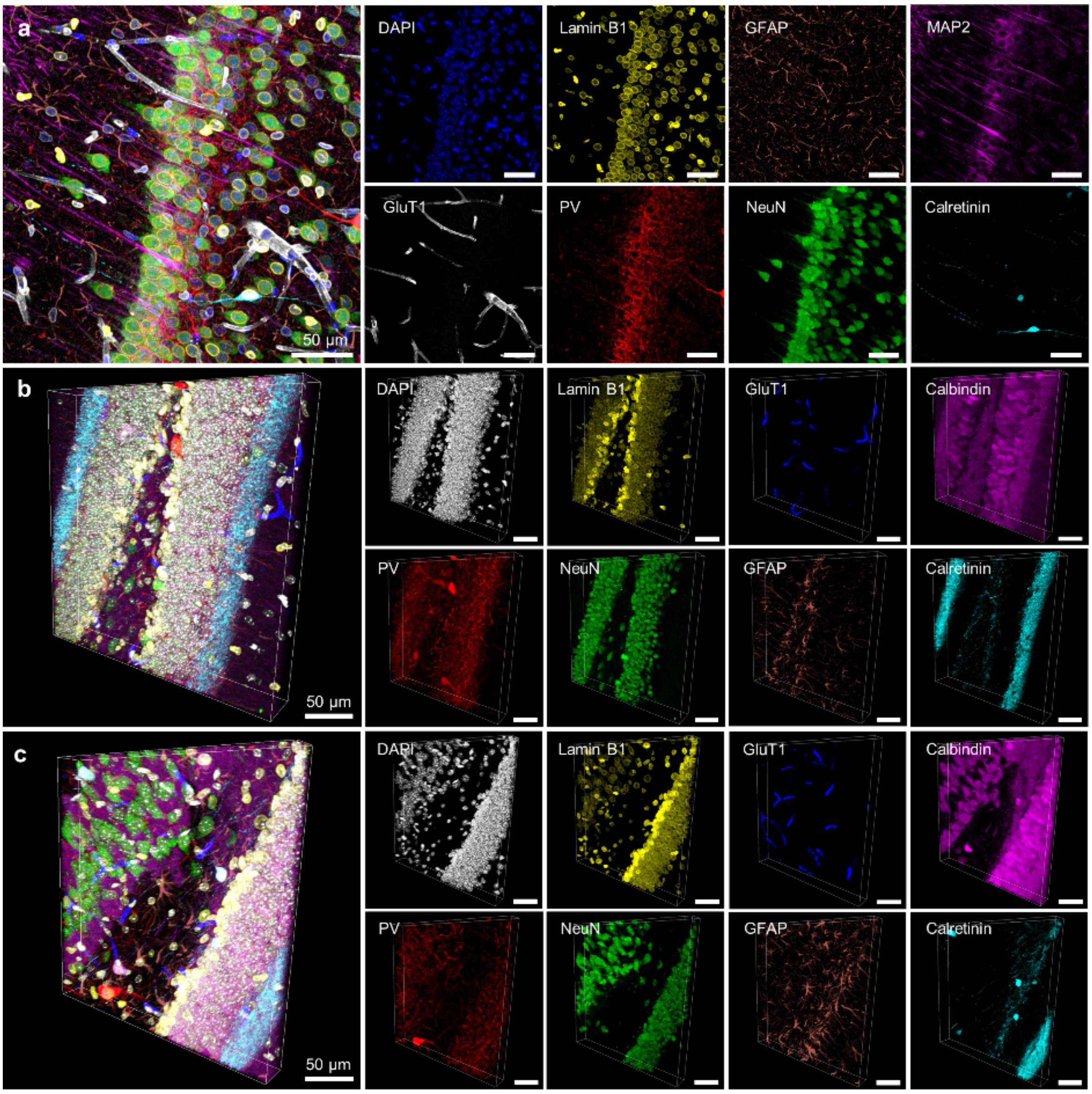

Supplementary Figure 26. **3D multiplexed imaging via PICASSO**. (**a**–**c**) Eight-color 3D multiplexed imaging of the mouse hippocampus. Seven preformed rabbit antibody complexes were used along with DAPI. (**a**) Maximum intensity projection (MIP) of the 8-color multiplexed z-stack image from **Fig**. **6a**. Individual MIPs are presented on the right side. (**b**–**c**) The 8-color 3D images acquired from the dentate gyrus in a different mouse brain slice. The z-stack images were acquired over a thickness of 40 μm with a step size of 0.5 μm.

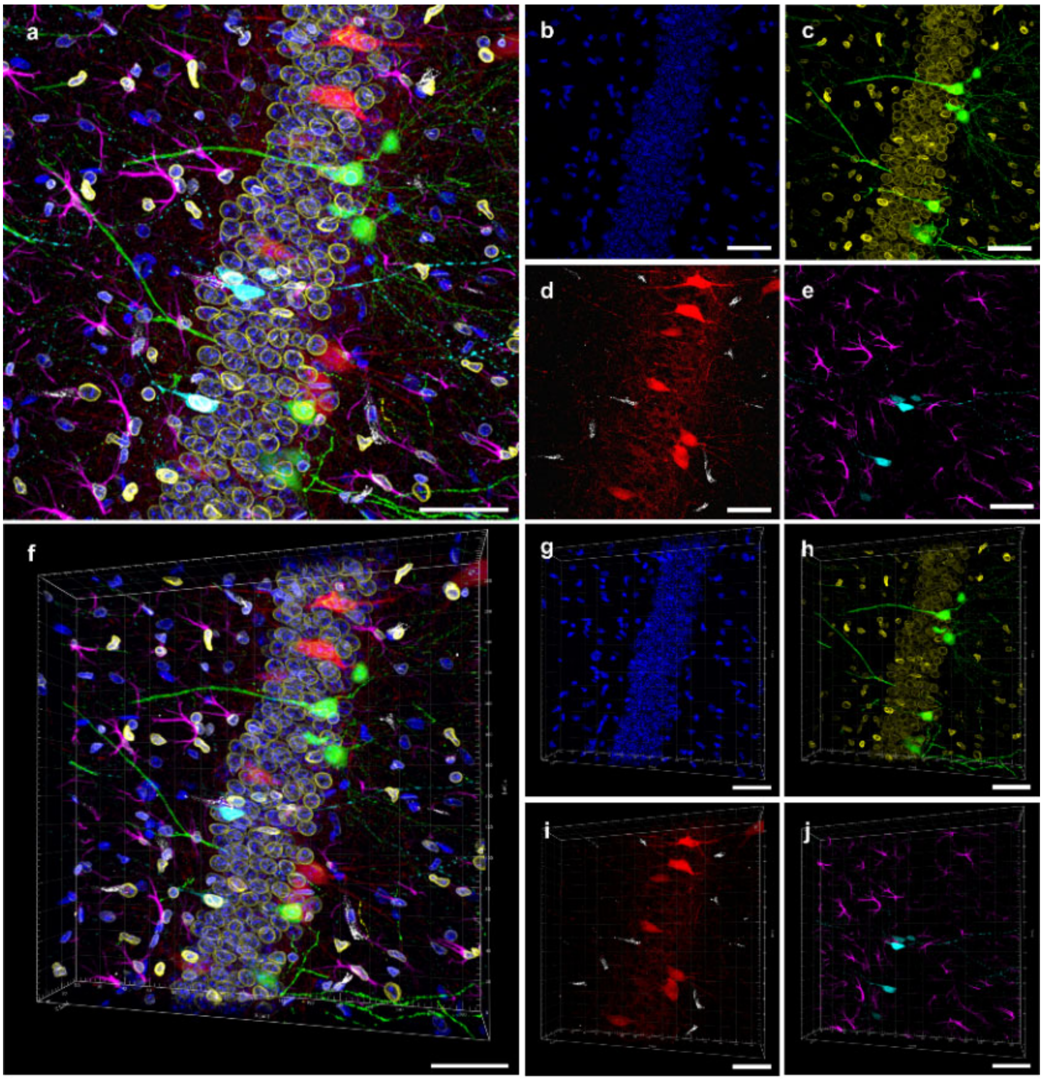

Supplementary Figure 27. **3D multiplexed imaging of a transgenic mouse brain slice via PICASSO**. Six proteins (including an endogenous YFP) and DAPI were imaged from a Thy1-YFP mouse brain slice at seven detection channels and unmixed via PICASSO. (**a**–**e**) Maximum intensity projection of a z-stack image. (**a**) Merged image. (**b**) DAPI (blue), acquired using a 405-nm laser. (**c**) Lamin B1 (yellow) and YFP (green), acquired using a 488-nm laser. (**d**) PV (red) and GluT1 (white), acquired using a 557-nm laser. (**e**) GFAP (magenta) and calretinin (cyan), acquired using a 640-nm laser. (**f**–**j**) A 3D view of the z-stack image shown in **a**–**e**. Scale bars: 50 µm.

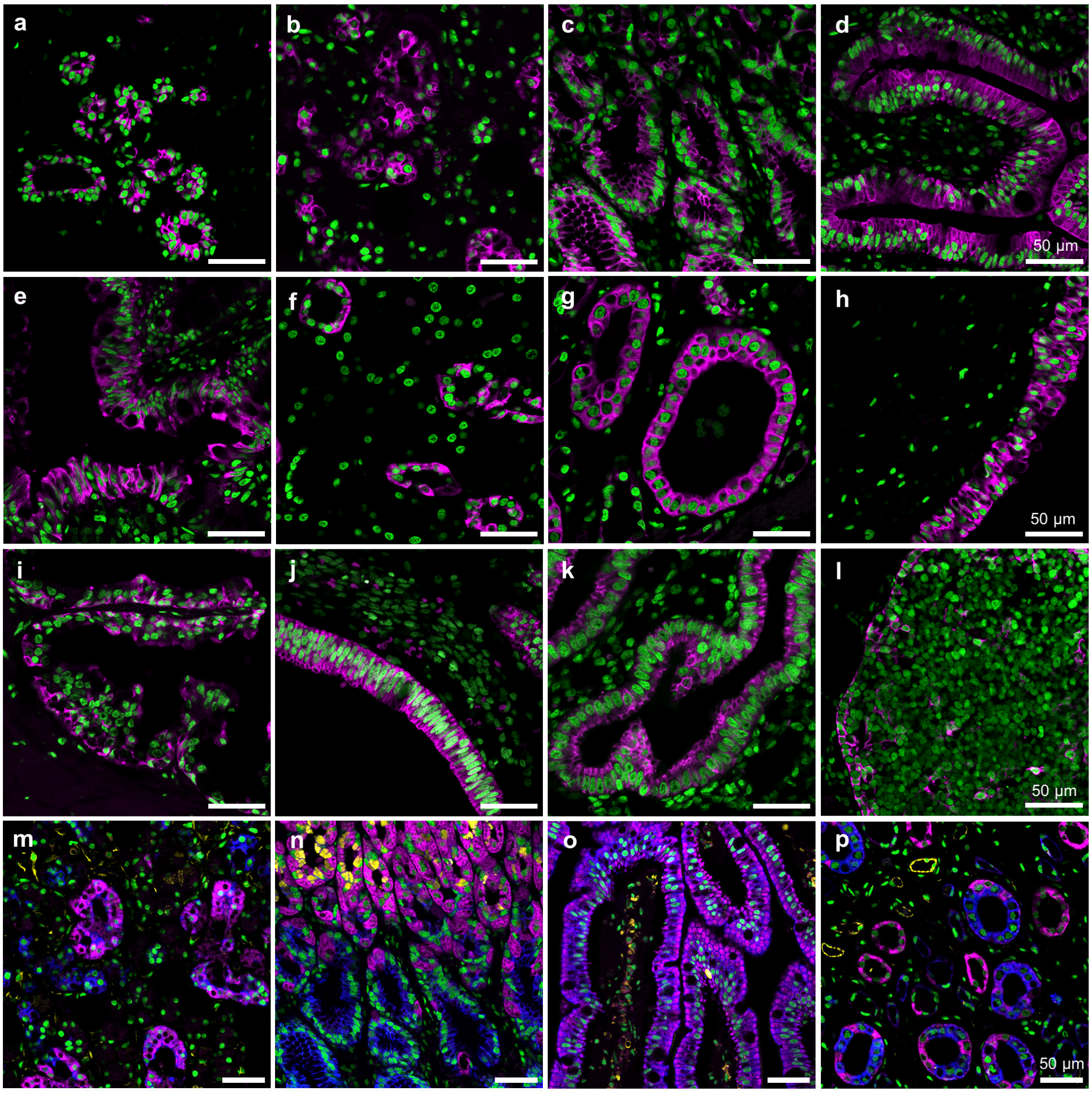

Supplementary Figure 28. **Multiplexed imaging of FFPE clinical samples via PICASSO**. (**a**–**l**) Two-color multiplexed imaging of 12 different human tissue types using one excitation laser enabled via PICASSO. Magenta, keratin 19 (CF488A); green, histone H3 (ATTO514). (**a**) Breast. (**b**) Salivary gland. (**c**) Stomach, body. (**d**) Small intestine, jejunum. (**e**) Colon. (**f**) Kidney, cortex. (**g**) Kidney, medulla. (**h**) Bladder. (**i**) Prostate. (**j**) Endometrium, proliferative. (**k**) Endometrium, secretory. (**l**) Thymus. (**m**–**p**) Four-color multiplexed imaging of four of the same human tissue types using two excitation lasers enabled via PICASSO. Blue, keratin 19 (CF488A); green, histone H3 (ATTO514); magenta, COX IV (CF568); yellow, vimentin (ATTORho101). (**m**) Salivary gland. (**n**) Stomach, body. (**o**) Small intestine, jejunum. (**p**) Kidney, medulla. (**a**–**p**) All primary antibodies were rabbit antibodies.

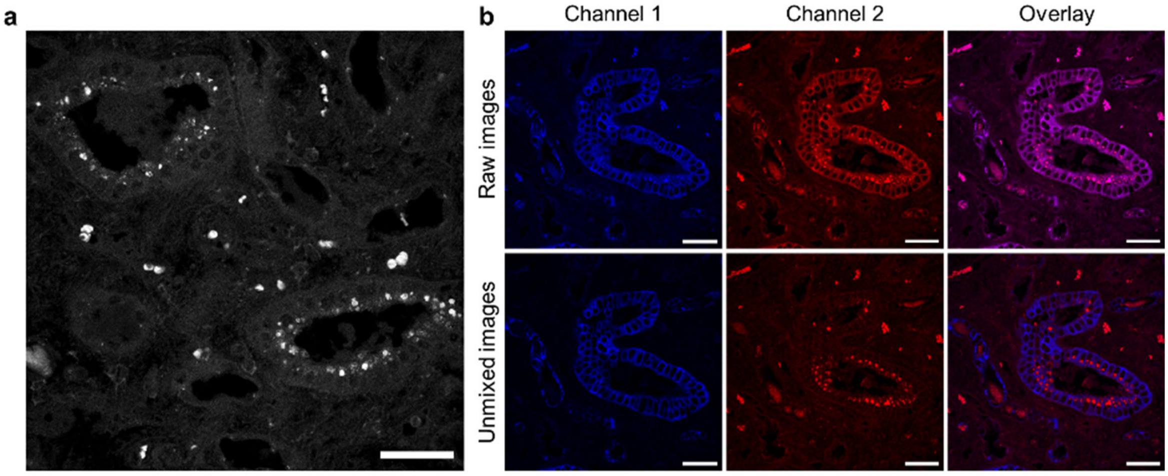

Supplementary Figure 29. **Removal of the autofluorescence from FFPE human kidney medulla via PICASSO**. (**a**) Image showing the autofluorescence of the specimen we used. (**b**) Removal of the autofluorescence via PICASSO. (**b**) FFPE sample was labeled with a preformed antibody complex against keratin (CF488A). Autofluorescence was considered as a separate fluorophore to remove it from the CF488A signal. Two images were acquired at two detection channels (490–525 nm and 525–560 nm) and then unmixed via PICASSO. Raw images were shown in the first row, and unmixed images were shown in the second row. In the second row, the blue signal is keratin (CF488A), and the red signal is autofluorescence. Note that the unmixed autofluorescence signal is similar to the autofluorescence signal acquired in a separate specimen shown in **a**.

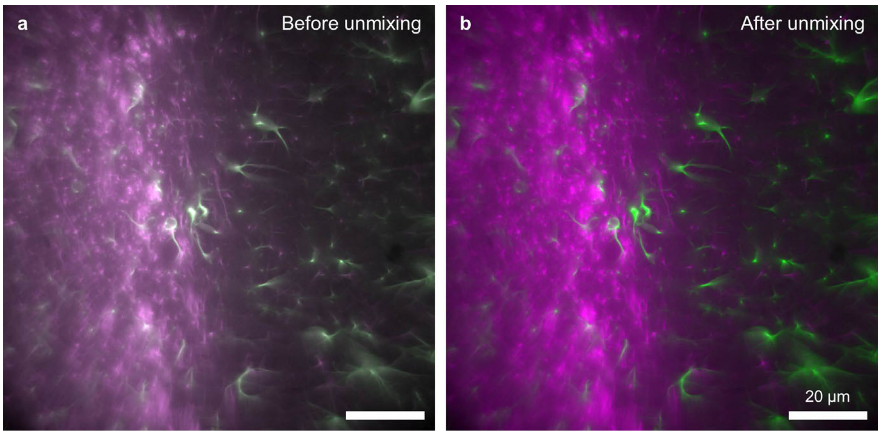

Supplementary Figure 30. **PICASSO unmixing result using a widefield microscope with a lamp light source**. A mouse brain slice was stained with GFAP (Alexa Fluor 488) and neurofilament-L (Alexa Fluor 532), and then, two mixed images were acquired using a widefield microscope equipped with a mercury lamp that used two emission filters (i.e., filter 1: 512–550 nm; filter 2: 542–582 nm). (**a**) Before unmixing. (**b**) After unmixing. Green; GFAP, magenta; neurofilament-L.

Supplementary Note 1. **Dependence of the emission spectra of fluorophores on optical**, **chemical**, **and environmental factors**.

The internal optics of microscopes[1], such as the presence of a notch filter[2] and wavelength-dependent quantum efficiency of a photon detector[3], strongly affect the measured emission spectra of fluorophores. In addition, some optical components are affected by environmental conditions; for example, the cut-off spectra of bandpass filters are sensitive to temperature changes due to the expansion or contraction of the coating materials[4]. Photomultiplier tube (PMT) detectors are also sensitive to temperature due to changes in the cathode sensitivity, especially at long wavelengths[5]. The chemical properties of solvents also affect emission spectra, such as the solvent polarity[6,7], pH[8,9], temperature[6], viscosity[6], internal charge transfer[6], and hydrogen bonding between fluorophore molecules and solvents[6]. Such solvent effects have become more important as diverse solvents such as dibenzyl ether, ethanol benzyl, iohexol, nicotinamide, antipyrine, sorbitol, N-methylacetamide, urea, DMSO, diatrizoic acid, n-methyl-d-glucamine, and deionized water have been introduced to recently developed tissue clearing, expansion, and shrinking techniques[10]. In addition, the emission spectra of fluorophores also depend on the local micro-environment inside cells or tissue slices[11–14]. The emission spectra also change when the fluorophores are conjugated to probes (e.g., antibodies)[15]. The mixing matrix, M, is the result of all of the above-mentioned optical, chemical, and environmental effects combined.

Supplementary Note 2. **PICASSO unmixing algorithm**.

Algorithm 1: PICASSO unmixing algorithm

1: **Input:** $\mathrm{D} \in \mathbb{R}^{m\times n}$

2: **Output:** $X \in \mathbb{R}^{m\times n}$

3: **initialize** $\mathrm{X} = D$

4: **while** *not converged* **do**

5: **construct** P as $n \times n$ identity matrix

6: **for all** (i, j) such that $0 \le i < N, 0 \le j < N, i \ne j$ **do**

7: **calculate** $\alpha_{i,j} = arg \min_{\alpha} I\left(q\left(bin(X_j)\right); q\left(bin(X_i - \alpha X_j)\right)\right)$

```
        P[i,j] = α_{i,j}
    end
    update X ← PX
end
return X
```

bin denotes image binning function
q denotes image quantization function

Here, we find the condition to guarantee the strict increase of the relative portion of the dominant channel in each iteration. For the sake of simplicity, we will consider the first iteration of the unmixing process when $N = 2$. The image formation (linear mixing) process can be expressed as follows:

$$\begin{bmatrix} D_1 \\ D_2 \end{bmatrix} = \begin{bmatrix} 1 & \alpha_{1,2} \\ \alpha_{2,1} & 1 \end{bmatrix} \begin{bmatrix} F_1 \\ F_2 \end{bmatrix} = \mathrm{M} \begin{bmatrix} F_1 \\ F_2 \end{bmatrix},$$

where $\alpha_{i,j}$ refers to the relative leakage from the j_{th} fluorophore to the i_{th} image; D_i and F_i are the i_{th} channels of the acquired and fluorophore images, respectively.

Without loss of generality, let's consider $i = 1$ and write the unmixing equation:

$$X_{1(1)} = D_1 - \gamma\alpha_{1,2(0)}D_2.$$

By substitution ($\varepsilon = \gamma\alpha_{1,2(0)}$), we obtain the following equation, where ε is a positive real number:

$$X_{1(1)} = D_1 - \varepsilon D_2.$$

Writing $X_{1(1)}$ as a linear summation of F_i gives:

$$X_{1(1)} = \left(F_1 + \alpha_{1,2}F_2\right) - \varepsilon\left(F_2 + \alpha_{2,1}F_1\right).$$

We can rearrange the equation as:

$$X_{1(1)} = \left(1 - \varepsilon\alpha_{2,1}\right)F_1 + \left(\alpha_{1,2} - \varepsilon\right)F_2.$$

The condition for the strict increase of the relative portion of the dominant channel can be written as:

$$\frac{1}{\alpha_{1,2}} < \frac{1-\varepsilon\alpha_{2,1}}{\alpha_{1,2}-\varepsilon}.$$

As the update parameter γ can be chosen to be arbitrarily small (and hence ε can be arbitrarily small), all terms are positive, and the inequality becomes as follows:

$$\alpha_{1,2} - \varepsilon < \alpha_{1,2}\left(1 - \varepsilon\alpha_{2,1}\right)$$

$$\therefore \varepsilon\left(1 - \alpha_{1,2}\alpha_{2,1}\right) > 0$$

$$\therefore 1 - \alpha_{1,2}\alpha_{2,1} > 0$$

$$\therefore \det(M) > 0.$$

Therefore, during the first iteration, the strict increase of the relative portion of the dominant channel is guaranteed if the determinant of the mixing matrix is positive. After the first iteration, $\alpha_{1,2}$ and $\alpha_{2,1}$ become smaller, so $1 - \alpha_{1,2}\alpha_{2,1} > 0$ is still met.

The same analogy can be extended to $N = 3$ and further:

$$\begin{bmatrix} D_1 \\ D_2 \\ D_3 \end{bmatrix} = \begin{bmatrix} 1 & \alpha_{1,2} & \alpha_{1,3} \\ \alpha_{2,1} & 1 & \alpha_{2,3} \\ \alpha_{3,1} & \alpha_{3,2} & 1 \end{bmatrix} \begin{bmatrix} F_1 \\ F_2 \\ F_3 \end{bmatrix} = \mathrm{M} \begin{bmatrix} F_1 \\ F_2 \\ F_3 \end{bmatrix}.$$

Again, without loss of generality, let's write the unmixing equation for $i = 1$:

$$X_{1(1)} = X_{1(0)} - \sum_{i \neq j} \gamma\alpha_{1,j(0)} X_{j(0)} = D_1 - \sum_{i \neq j} \gamma\alpha_{1,j(0)} D_j = D_1 - \gamma\alpha_{1,2(0)} D_2 - \gamma\alpha_{1,3(0)} D_3.$$

By substitution ($\varepsilon = \gamma\alpha_{1,2(0)}$ and $\delta = \gamma\alpha_{1,3(0)}$), we can re-write as follows, where ε and δ are both positive real numbers:

$$X_{1(1)} = D_1 - \varepsilon D_2 - \delta D_3.$$

If each subtraction increases the relative portion of the dominant channel, then performing all subtractions will also strictly increase the relative portion of the dominant channel. Therefore, it suffices to consider two cases separately: 1) $\varepsilon > 0$ and $\delta = 0$ and 2) $\varepsilon = 0$ and $\delta > 0$.

From 1), writing $X_{1(1)}$ as a linear summation of F_i gives:

$$X_{1(1)} = (F_1 + \alpha_{1,2}F_2 + \alpha_{1,3}F_3) - \varepsilon(F_2 + \alpha_{2,1}F_1 + \alpha_{2,3}F_3)$$

$$\therefore X_{1(1)} = (1 - \varepsilon\alpha_{2,1})F_1 + (\alpha_{1,2} - \varepsilon)F_2 + (\alpha_{1,3} - \varepsilon\alpha_{2,3})F_3.$$

The condition for the strict increase of the relative portion of the dominant channel can be written as:

$$\frac{1}{\alpha_{1,2}} < \frac{1-\varepsilon\alpha_{2,1}}{\alpha_{1,2}-\varepsilon} \text{ and } \frac{1}{\alpha_{1,3}} < \frac{1-\varepsilon\alpha_{2,1}}{\alpha_{1,3}-\varepsilon\alpha_{2,3}}$$

$$\therefore \varepsilon(1 - \alpha_{1,2}\alpha_{2,1}) > 0 \text{ and } \varepsilon(\alpha_{2,3} - \alpha_{1,3}\alpha_{2,1}) > 0$$

$$\therefore (1 - \alpha_{1,2}\alpha_{2,1}) > 0 \text{ and } (\alpha_{2,3} - \alpha_{1,3}\alpha_{2,1}) > 0.$$

We find that both terms of the left-hand side are the determinants of the 2×2 sub-matrices of the original mixing matrix M.

From 2), similarly, we also obtain that the determinants of the other 2×2 sub-matrices of the original mixing matrix M need to be positive. In extension, the determinants of all possible 2×2 sub-matrices of the mixing matrix, which includes diagonal entities, need to be positive to guarantee the strict increase of the relative portion of the dominant channel.

Supplementary Note 3. **Biological contexts of Fig**. **5a**. Calbindin is a calcium-binding protein, and its spatial expression level in the mouse brain was found to be high in the dentate gyrus (DG) along the mossy fiber projections and relatively low in the thalamus (TH) (**Fig**. **5a** and **Supplementary Fig**. **19b**), consistent with the literature[16]. Calretinin is a calcium-binding protein that is highly expressed in the mammalian brain[17]. Calretinin expression was found to be high in a thin layer of the molecular layer (DG-mo) and granule cell layer (DG-sg) of the DG and TH (**Fig**. **5a** and **Supplementary Fig**. **19d**) and matched well with the database (row 1 of **Supplementary Table 3**). ZNF3 is a transcription factor that is highly expressed in the mouse brain (row 2 of **Supplementary Table 3**). Consistent with the database, ZNF3 was found to be highly expressed in the DG-sg and polymorph layer (DG-po) of the DG and TH below the DG, as shown in **Fig**. **5a** and **Supplementary Fig**. **19e**. NeuN is a neuron marker that is highly expressed exclusively only in the brain (row 3 of **Supplementary Table 3**). As shown in **Fig**. **5a** and **Supplementary Fig**. **19c**, NeuN was found to be expressed in DG-sg, DG-po, and TH, but its expression was higher in DG than in TH, consistent with the literature[18]. Parvalbumin (PV) is a calcium-binding albumin protein that is highly expressed in the mouse brain (row 4 of **Supplementary Table 3**). PV was found to be widely expressed in DG-po, DG-sg, DG-mo, and TH (**Fig**. **5a** and **Supplementary Fig**. **19a**), again consistent with the

literature[19].

Channel #	Fluorophore	Emission peak (nm)	Abbreviation
Ch 1 (645–655 nm)	CF633	650	CF633
	Alexa Fluor 633	647	AF633
Ch 2 (663–673 nm)	Alexa Fluor 647	668	AF647
	ATTO647N	669	AT647N
Ch 3 (678–688 nm)	CF660R	683	CF660R
	ATTO665	685	AT665
Ch 4 (696–706 nm)	CF680R	701	CF680R
	Alexa Fluor 680	702	AF680
Ch 5 (714–724 nm)	ATTO700	719	AT700
	Alexa Fluor 700	719	AF770

#	Ch 1	Ch 2	Ch 3	Ch 4	Ch 5	Correlation w/ GT	Determinants of all 2×2 matrices
1	CF633	AF647	CF660R	CF680R	AT700	0.984	Positive
2	CF633	AF647	CF660R	CF680R	AF770	0.985	Positive
3	CF633	AF647	CF660R	AF680	AT700	0.984	Positive
4	CF633	AF647	CF660R	AF680	AF770	0.984	Positive
5	CF633	AF647	AT665	CF680R	AT700	0.984	Positive
6	CF633	AF647	AT665	CF680R	AF770	0.984	Positive
7	CF633	AF647	AT665	AF680	AT700	0.985	Positive
8	CF633	AF647	AT665	AF680	AF770	0.985	Positive
9	CF633	AT647N	CF660R	CF680R	AT700	0.983	Positive
10	CF633	AT647N	CF660R	CF680R	AF770	0.985	Positive
11	CF633	AT647N	CF660R	AF680	AT700	0.985	Positive
12	CF633	AT647N	CF660R	AF680	AF770	0.985	Positive
13	CF633	AT647N	AT665	CF680R	AT700	0.984	Positive
14	CF633	AT647N	AT665	CF680R	AF770	0.985	Positive
15	CF633	AT647N	AT665	AF680	AT700	0.985	Positive
16	CF633	AT647N	AT665	AF680	AF770	0.984	Positive
17	AF633	AF647	CF660R	CF680R	AT700	0.983	Positive
18	AF633	AF647	CF660R	CF680R	AF770	0.985	Positive
19	AF633	AF647	CF660R	AF680	AT700	0.984	Positive
20	AF633	AF647	CF660R	AF680	AF770	0.985	Positive
21	AF633	AF647	AT665	CF680R	AT700	0.985	Positive
22	AF633	AF647	AT665	CF680R	AF770	0.985	Positive
23	AF633	AF647	AT665	AF680	AT700	0.985	Positive
24	AF633	AF647	AT665	AF680	AF770	0.985	Positive
25	AF633	AT647N	CF660R	CF680R	AT700	0.981	Positive
26	AF633	AT647N	CF660R	CF680R	AF770	0.985	Positive
27	AF633	AT647N	CF660R	AF680	AT700	0.985	Positive
28	AF633	AT647N	CF660R	AF680	AF770	0.985	Positive
29	AF633	AT647N	AT665	CF680R	AT700	0.985	Positive
30	AF633	AT647N	AT665	CF680R	AF770	0.986	Positive
31	AF633	AT647N	AT665	AF680	AT700	0.985	Positive
32	AF633	AT647N	AT665	AF680	AF770	0.985	Positive

Supplementary Table 1. **Unmixing of 32 different fluorophore combinations**. We tested whether PICASSO could unmix various fluorophore combinations even when the same detection channels are used. For each of the five fluorophores we used in **Fig**. **2g–i** (CF633, Alexa Fluor 647, CF660R, CF680R, and ATTO700), we chose an additional fluorophore with a similar emission spectrum. We chose Alexa Fluor 633 for CF633, ATTO647N for Alexa Fluor 647, ATTO665 for CF660R, Alexa Fluor 680 for CF680R, and Alexa Fluor 700 for ATTO700 (first table). For these five pairs of fluorophores, we generated 32 (=2^5) different 5-color combinations and calculated mixing matrices of these 32 combinations based on the reference emission spectra of the fluorophores. When calculating the mixing matrices, the five detection channels shown in **Fig**. **2g** were used. We then synthesized mixed imaging by using the calculated mixing matrices and single-channel images shown in **Fig**. **2h** and unmixed them via PICASSO. The structural similarity (SSIM) of unmixing of all 32 fluorophore combinations were above 0.98, as shown in the second table. In addition, the determinants of all 2 × 2 sub-matrices of the mixing matrices of the 32 combinations met the non-negativity condition, as shown in the second table. This result indicates that PICASSO can unmix various fluorophore combinations with fixed detection channels. This result also suggests that PICASSO works for a given fluorophore set without changing or optimizing detection channels, even their emission spectra shift few nanometers due to the chemical or environmental factors shown in **Supplementary Note 1**.

#	Dye	Vendor	Catalog#
1	ATTO390	ATTO-TEC	AD 390-31
2	CF405S	Biotium	#92110
3	CF405M	Biotium	#92111
4	CF405L	Biotium	#92112
5	Dylight405	Life technologies	46400
6	ATTO430LS	ATTO-TEC	AD 430LS-31
7	CF488A	Biotium	#92120
8	ATTO488	ATTO-TEC	AD 488-31
9	Alexa Fluor 488	Life technologies	A20000
10	ATTO490LS	ATTO-TEC	AD 490LS-31
11	ATTO514	ATTO-TEC	AD 514-31
12	CF514	Biotium	#92103
13	ATTO532	ATTO-TEC	AD 532-31
14	Alexa Fluor 546	Life technologies	A20002
15	ATTO565	ATTO-TEC	AD 565-31
16	CF568	Biotium	#92131
17	ATTO Rho101	ATTO-TEC	AD Rho101-31
18	Alexa Fluor 594	JacksonImmunoResearch	111-587-008
19	ATTO594	ATTO-TEC	AD 594-31
20	ATTO633	ATTO-TEC	AD 633-31
21	CF633	Biotium	#92133
22	Alexa Fluor 647	JacksonImmunoResearch	111-607-008
23	ATTO647N	ATTO-TEC	AD 647N-31
24	Alexa Fluor 680	JacksonImmunoResearch	111-627-008
25	CF660R	Biotium	#92134
26	CF680R	Biotium	#92107
27	ATTO725	ATTO-TEC	AD 725-31
28	Alexa Fluor 790	JacksonImmunoResearch	111-657-008

Supplementary Table 2. **List of the fluorophores that worked with the primary antibody–Fab preformation technique**.

#	Name	Database	Link
1	Calretinin	The Human Protein Atlas	https://www.proteinatlas.org/ENSG00000172137-CALB2/brain
2	ZNF3	The Human Protein Atlas	https://www.proteinatlas.org/ENSG00000166526-ZNF3
3	NeuN	The Human Protein Atlas	https://www.proteinatlas.org/ENSG00000167281-RBFOX3
4	PV	Allen Brain Atlas	http://mouse.brain-map.org/gene/show/19056
5	Keratin 19	The Human Protein Atlas	https://www.proteinatlas.org/ENSG00000171345-KRT19/tissue
6	H3F3A	The Human Protein Atlas	https://www.proteinatlas.org/ENSG00000163041-H3F3A/tissue

Supplementary Table 3. **Databases used to cross-validate the protein expression patterns observed by PICASSO**.

Filter number	CWL (nm)	FWHM (nm)	Vendor	Product number	Fluorophore (wavelength of the used excitation laser)
1	425	25	Edmund	#87-787	CF405S (Exc: 405 nm)
2	466	45.3	Semrock	FF01-466/40-25	ATTO390 (Exc: 405 nm)
3	504	17	Semrock	FF01-504/12-25	CF488A (Exc: 488 nm)
4	540	55.6	Semrock	FF01-540/50-25	CF405L (Exc: 405 nm)<br>ATTO514 (Exc: 488 nm)
5	575	20.1	Semrock	FF01-575/15-25	CF568 (Exc: 561 nm)
6	607	42	Edmund	#84-102	ATTORho101 (Exc: 561 nm)
7	656	10	Andover	656HC10-25	CF633 (Exc: 637 nm)
8	680	47	Semrock	FF01-680/42-25	ATTO490LS (Exc: 488 nm)<br>CF660R (Exc: 637 nm)

Supplementary Table 4. **List of the bandpass filters used with the confocal microscopy system**. Exc: wavelength of an excitation laser.

References

1. Zimmermann, T., Marrison, J., Hogg, K. & O'Toole, P. Clearing Up the Signal: Spectral Imaging and Linear Unmixing in Fluorescence Microscopy. in *Confocal Microscopy: Methods and Protocols* (ed. Paddock, S. W.) 129–148 (Springer New York, 2014). doi:10.1007/978-1-60761-847-8_5
2. Valm, A. M. *et al.* Applying systems-level spectral imaging and analysis to reveal the organelle interactome. *Nature* **546**, 162–167 (2017).
3. Flyckt, S. & Marmonier, C. Photomultiplier tubes: Principles and applications. *Photonis, Brive, Fr.* (2002). doi:10.1117/3.682726.p35
4. William Ross McCluney. *Introduction to Radiometry and Photometry 2nd ed* (Artech House Publishers, Norwood, 2014).
5. Tony Wright, A. G. W. *The Photomultiplier Handbook* (Oxford Univ. Press, Oxford, 2017).
6. Joseph R. Lakowicz. *Principles of Fluorescence Spectroscopy Ch 6* (Springer, Boston, 2006). doi:10.1007/978-0-387-46312-4_6
7. Zehentbauer, F. M. *et al.* Fluorescence spectroscopy of Rhodamine 6G: Concentration and solvent effects. *Spectrochim. Acta Part A Mol. Biomol. Spectrosc.* **121**, 147–151 (2014).
8. Doughty, M. J. PH dependent spectral properties of sodium fluorescein ophthalmic solutions revisited. *Ophthalmic Physiol. Opt.* (2010). doi:10.1111/j.1475-1313.2009.00703.x
9. Martin, M. M. & Lindqvist, L. The pH dependence of fluorescein fluorescence. *J. Lumin.* **10**, 381–390 (1975).
10. Richardson, D. S. & Lichtman, J. W. SnapShot: Tissue Clearing. *Cell* **171**, 496-496.e1 (2017).
11. Tsurui, H. *et al.* Hyperspectral imaging of pathology samples. in *Proc.SPIE* **3605**, (1999).
12. Barash, E., Dinn, S., Sevinsky, C. & Ginty, F. Multiplexed analysis of proteins in tissue using multispectral fluorescence imaging. *IEEE Trans. Med. Imaging* (2010). doi:10.1109/TMI.2010.2045005
13. Cohen, S., Valm, A. M. & Lippincott-Schwartz, J. Multispectral Live-Cell Imaging. *Curr. Protoc. Cell Biol.* **79**, e46 (2018).
14. Tsurui, H. *et al.* Seven-color Fluorescence Imaging of Tissue Samples Based on Fourier Spectroscopy and Singular Value Decomposition. *J. Histochem. Cytochem.* **48**, 653–662 (2000).
15. Wazawa, T., Ishii, Y., Funatsu, T. & Yanagida, T. Spectral fluctuation of a single fluorophore conjugated to a protein molecule. *Biophys. J.* (2000). doi:10.1016/S0006-3495(00)76708-7
16. Ramírez-Rodríguez, B. G., Olvera-Hernández, S., Vega-Rivera, M. N. & Ortiz-López, L. Melatonin

Influences Structural Plasticity in the Axons of Granule Cells in the Dentate Gyrus of Balb/C Mice. *Int. J. Mol. Sci.* **20**, 73 (2018).

17. Résibois, A. & Rogers, J. H. Calretinin in rat brain: An immunohistochemical study. *Neuroscience* (1992). doi:10.1016/0306-4522(92)90012-Q
18. Wang, H.-Y. *et al.* RBFOX3/NeuN is Required for Hippocampal Circuit Balance and Function. *Sci. Rep.* **5**, 17383 (2015).
19. Milenkovic, I. *et al.* The parvalbumin-positive interneurons in the mouse dentate gyrus express GABAA receptor subunits alpha1, beta2, and delta along their extrasynaptic cell membrane. *Neuroscience* **254**, 80–96 (2013).
