# Supplementary material for: PICASSO allows ultra-multiplexed fluorescence imaging of spatially overlapping proteins without reference spectra measurements
Source: Nat Commun. 2022 May 5;13:2475. doi: 10.1038/s41467-022-30168-z (PMC9072354; doi:10.1038/s41467-022-30168-z)
Supplement: Supplementary file 2 — Reporting Summary [file 41467_2022_30168_MOESM2_ESM.pdf]

nature research

Corresponding author(s): Jae-Byum Chang, Young-Gyu Yoon

Last updated by author(s): Apr 14, 2022

Reporting Summary

Nature Research wishes to improve the reproducibility of the work that we publish. This form provides structure for consistency and transparency in reporting. For further information on Nature Research policies, see Authors & Referees and the Editorial Policy Checklist .

Statistics

For all statistical analyses, confirm that the following items are present in the figure legend, table legend, main text, or Methods section.

n/a	Confirmed	
[ ]	[x]	The exact sample size (*n*) for each experimental group/condition, given as a discrete number and unit of measurement
[ ]	[x]	A statement on whether measurements were taken from distinct samples or whether the same sample was measured repeatedly
[x]	[ ]	The statistical test(s) used AND whether they are one- or two-sided<br>*Only common tests should be described solely by name; describe more complex techniques in the Methods section.*
[x]	[ ]	A description of all covariates tested
[x]	[ ]	A description of any assumptions or corrections, such as tests of normality and adjustment for multiple comparisons
[ ]	[x]	A full description of the statistical parameters including central tendency (e.g. means) or other basic estimates (e.g. regression coefficient) AND variation (e.g. standard deviation) or associated estimates of uncertainty (e.g. confidence intervals)
[x]	[ ]	For null hypothesis testing, the test statistic (e.g. *F*, *t*, *r*) with confidence intervals, effect sizes, degrees of freedom and *P* value noted<br>*Give P values as exact values whenever suitable.*
[x]	[ ]	For Bayesian analysis, information on the choice of priors and Markov chain Monte Carlo settings
[x]	[ ]	For hierarchical and complex designs, identification of the appropriate level for tests and full reporting of outcomes
[x]	[ ]	Estimates of effect sizes (e.g. Cohen's *d*, Pearson's *r*), indicating how they were calculated

Our web collection on statistics for biologists contains articles on many of the points above.

Software and code

Policy information about availability of computer code

Data collection	Leica LAS X v4.12.0 (for Leica SP8), Fusion v2.1.0.34 (for Andor spinning disk confocal), NIS-Elements AR v5.11.01 (for Nikon C2 plus)
Data analysis	Microsoft Excel 2019, Microsoft Powerpoint 2019, Imaris v9.2, ImageJ/Fiji v1.53p, MATLAB R2019b v9.7.0, OriginPro 2019 v9.6.0.172 (academic) and BaSiC Plugin in ImageJ/Fiji v1.53p.

For manuscripts utilizing custom algorithms or software that are central to the research but not yet described in published literature, software must be made available to editors/reviewers. We strongly encourage code deposition in a community repository (e.g. GitHub). See the Nature Research guidelines for submitting code & software for further information.

Data

Policy information about availability of data

All manuscripts must include a data availability statement. This statement should provide the following information, where applicable:

- Accession codes, unique identifiers, or web links for publicly available datasets
- A list of figures that have associated raw data
- A description of any restrictions on data availability

The unmixing data generated in this study have been deposited in the Figshare database under accession code https://doi.org/10.6084/m9.figshare.19596682.v1. Source data are provided with this paper.

Field-specific reporting

Please select the one below that is the best fit for your research. If you are not sure, read the appropriate sections before making your selection.

Life sciences study design

All studies must disclose on these points even when the disclosure is negative.

Sample size	We tested our imaging method with more than five separate cell cultures, more than ten mouse brains, and more than ten clinical specimens from a single tissue microarray. We thought the sample size was sufficient for proving the efficacy of our multiplexed imaging technique.
Data exclusions	None of data were excluded.
Replication	The method demonstrated in this work was applied on various samples (mouse brain slices, BS-C-1 cells, HeLa cells, NIH-3T3 cells, and various FFPE human tissue samples) and successfully reproduced the results without any significant deviation. For reproduction, anyone can access the codes enclosed in Supplementary Software.
Randomization	Not applicable because the study is not investigating more than two populations.
Blinding	Not applicable because the study is not investigating more than two populations.

Reporting for specific materials, systems and methods

We require information from authors about some types of materials, experimental systems and methods used in many studies. Here, indicate whether each material, system or method listed is relevant to your study. If you are not sure if a list item applies to your research, read the appropriate section before selecting a response.

Materials & experimental systems

n/a	Involved in the study	
☐	☒	Antibodies
☐	☒	Eukaryotic cell lines
☒	☐	Palaeontology
☐	☒	Animals and other organisms
☒	☐	Human research participants
☒	☐	Clinical data

Methods

n/a	Involved in the study	
☒	☐	ChIP-seq
☒	☐	Flow cytometry
☒	☐	MRI-based neuroimaging

Antibodies

Antibodies used	Detailed information about antibodies used in this study has been provided in Supplementary Data 1.
Validation	Antibodies purchased from the Human Protein Atlas (HPA) were validated by the Human Protein Atlas project (Website: https://www.proteinatlas.org/, References: Uhlén, M. et al. Tissue-based map of the human proteome. Science (80-.). (2015). doi:10.1126/science.1260419). Detailed specification and validation data of the antibodies purchased from other vendors (Abcam, novus biologicals, synaptic systems, merck millipore, encor biotechnology, sigma aldrich, immunostar, cell signaling technology, thermofisher scientific, and kerafast) were found in manufacturer's website. Also, vendor, catalog number, host, clonality, dilution, reactivity and application of each antibody have been provided in Supplementary Data 1.

Eukaryotic cell lines

Policy information about cell lines

Cell line source(s)	BS-C-1 cells , HeLa cells and NIH-3T3 cells were purchased from the Korean Cell Line Bank .
Authentication	Cell authentication test was not performed in this study.
Mycoplasma contamination	Mycoplasma contamination test was not conducted on this study.
Commonly misidentified lines (See ICLAC register)	No commonly misidentified lines were used in this study.

Animals and other organisms

Policy information about studies involving animals; ARRIVE guidelines recommended for reporting animal research

Laboratory animals	Thy1-YFP and C57BL/6J male mice ages 8-14 weeks were used for mouse brain imaging.
Wild animals	No wild animals were used in the study.

Field-collected samples	No field-collected samples were used in the study.
Ethics oversight	All the procedures involving animals were approved by the Sungkyunkwan University Institutional Animal Care and Use Committee (SKKU-IACUC, approved protocol number, SKKUIACUC-17-10-8-1), and the Korea Advanced Institute of Science and Technology Institutional Animal Care and Use Committee (KAIST-IACUC, approved protocol number, KA2020-48).

Note that full information on the approval of the study protocol must also be provided in the manuscript.
