# Supplementary material for: PICASSO allows ultra-multiplexed fluorescence imaging of spatially overlapping proteins without reference spectra measurements
Source: Nat Commun. 2022 May 5;13:2475. doi: 10.1038/s41467-022-30168-z (PMC9072354; doi:10.1038/s41467-022-30168-z)
Supplement: Supplementary file 3 — Description of Additional Supplementary Files [file 41467_2022_30168_MOESM3_ESM.pdf]

Title: Supplementary Movie 1.
Description: Sample preparation procedure for the 10-color multiplexed imaging of a mouse brain slice.

Title: Supplementary Movie 2.
Description: 3D 8-color multiplexed imaging of a mouse brain slice. See Fig. 6a for details. Blue, DAPI; yellow, lamin B1; brown, GFAP; magenta, MAP2; white, GluT1; red, PV; green, NeuN; cyan, calretinin.

Title: Supplementary Movie 3.
Description: Acquisition of the 10-color multiplexed image. A mouse brain slice stained with 10 preformed antibody complexes conjugated with 10 spectrally overlapping fluorophores was imaged by using a spinning-disk confocal microscopy system.

Title: Supplementary Data 1.
Description: List of the tested primary antibodies.

Title: Supplementary Data 2.
Description: Microscopy, antibodies, and fluorophores used for the PICASSO imaging.

Title: Supplementary Data 3.
Description: List of the materials used in this study.

Title: Supplementary Software.
Description: Unmixing code of PICASSO. Custom MATLAB code unmixing three images of a specimen labeled with three spectrally overlapping fluorophores via MI minimization.
