# Supplementary material for: PICASSO allows ultra-multiplexed fluorescence imaging of spatially overlapping proteins without reference spectra measurements
Source: Nat Commun. 2022 May 5;13:2475. doi: 10.1038/s41467-022-30168-z (PMC9072354; doi:10.1038/s41467-022-30168-z)

# <Instruction>

Journal : Nature communications

Title : PICASSO : Ultra-multiplexed fluorescence imaging of spatially overlapping proteins without reference spectra measurements

Authors : Junyoung Seo, Yeonbo Sim, Jeewon Kim, Hyunwoo Kim, In Cho, Hoyeon Nam, Young-Gyu Yoon, and Jae-Byum Chang

Affiliation : Korea Advanced Institute of Science and Technology

- **Minimum requirements of the code**
  - ✓ To run this code, MATLAB (Mathworks) should be installed. In addition, MATLAB Image Processing Toolbox and Wavelet Toolbox are required to run unmixing code.
  - ✓ We have tested the code with MATLAB R2020b running on Windows 10.

- **Demo image**
  - ✓ A demo input image is in the 'Data' folder, named '3color_data.tif'.
  - ✓ The demo input image was acquired by a single excitation laser but within three different spectral ranges from a mouse brain slice labelled with three preformed rabbit antibody complexes (PV-CF488A ,Neun-ATTO514, GFAP-ATTO532 ).
  - ✓ Simply running the code ('three_color_unmixing.m') will generate an unmixed image, named '3color_data_unmixed.tif' in the 'Results' folder.

- **How to modify the code to unmix other images**
  - ✓ Input files should be a tif format images, having three channels where the spectral detection range includes the corresponding fluorophore's emission peak (input $IMG1$, input $IMG\,2$, and input $IMG\,3$ shown in Fig. 3a).

    1. **Copy your input image files to the 'Data' folder**

    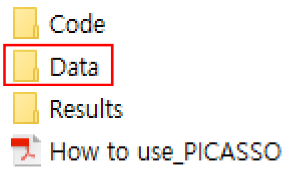
    

    2. **Open the code in the 'Code' folder**

    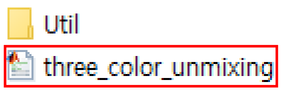
    

    3. **Change the 'filename' as the name of your input image file**

```
imgPath = '../Data/';
filename = '3color_data.tif';
```

4. **Run the unmixing code**

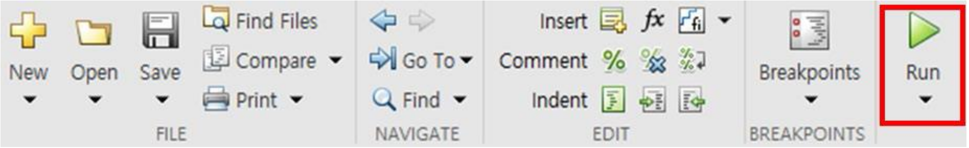


5. **Resultant unmixed images are saved in the 'Results' folder, named '[filename]_unmixed.tif'.**

6. **Input mixed images and unmixed images both can be displayed in individual channel mode and composite mode through the imageJ software program, as shown below.**

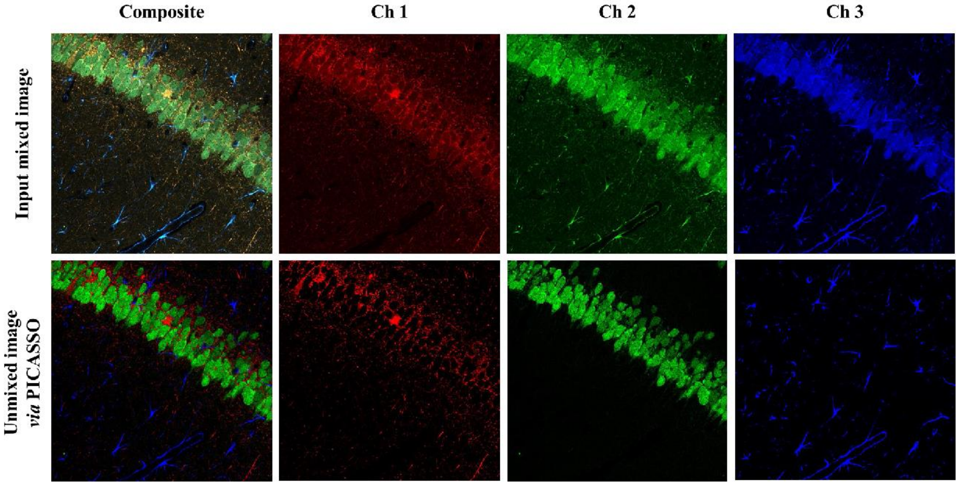

Supplement: Supplementary file 10 — Supplementary Software [file 41467_2022_30168_MOESM10_ESM.zip › Supplementary Software/Supplementary Software. Unmixing code of PICASSO/How to use_PICASSO.pdf]
