# Supplementary figures and images for: PICASSO allows ultra-multiplexed fluorescence imaging of spatially overlapping proteins without reference spectra measurements
Source: Nat Commun. 2022 May 5;13:2475. doi: 10.1038/s41467-022-30168-z (PMC9072354; doi:10.1038/s41467-022-30168-z)

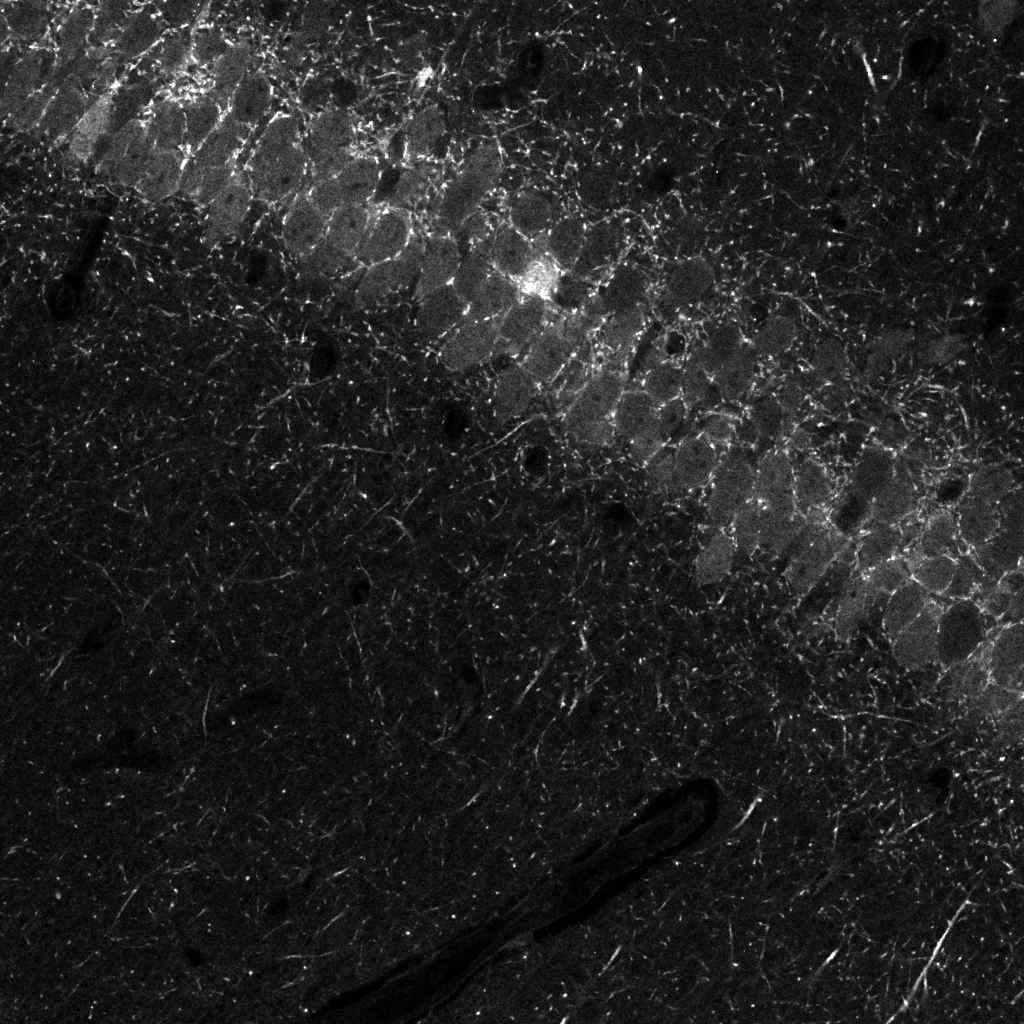

Supplement: Supplementary file 10 — Supplementary Software [file 41467_2022_30168_MOESM10_ESM.zip › Supplementary Software/Supplementary Software. Unmixing code of PICASSO/Data/3color_data.tif]

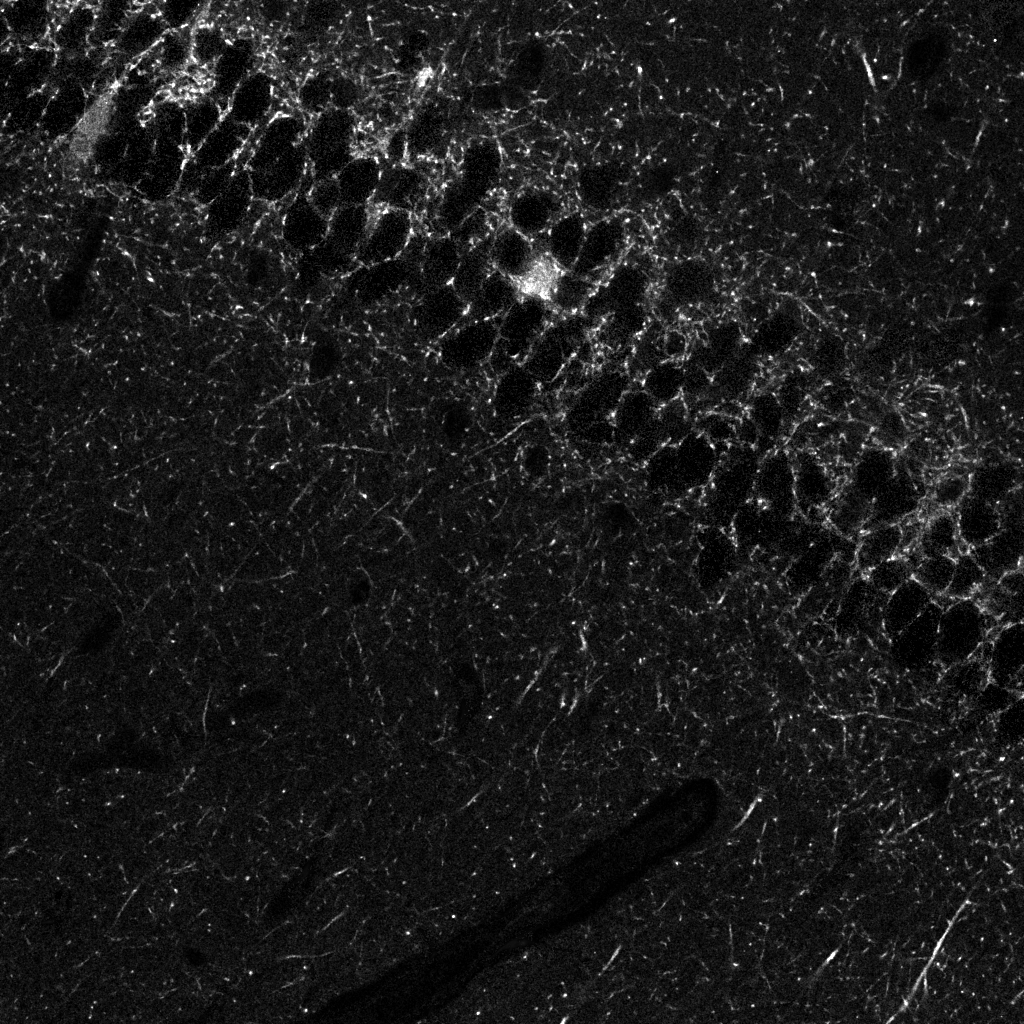

Supplement: Supplementary file 10 — Supplementary Software [file 41467_2022_30168_MOESM10_ESM.zip › Supplementary Software/Supplementary Software. Unmixing code of PICASSO/Results/3color_data_unmixed.tif]
